# Randomised treatment of acute pancreatitis with infliximab: protocol for a double-blind, placebo-controlled, multi-centre, adaptive, phase 2 superiority trial (RAPID-I)

**DOI:** 10.1136/bmjopen-2026-118729

**Published:** 2026-09-18

**Authors:** Juan Lin, Matt Smyth, Catherine Spowart, Michaela Brown, Ian Powell, Diane Latawiec, Di Wu, Wei Huang, Rajarshi Mukherjee, Peter Szatmary, Ryan Baron, Declan Dunne, Jonathan Evans, Sidharth Srinivas, Giles Bond-Smith, Jamie Cooper, Andrew Smith, Antonio Manzelli, Samer Elkhodair, David O’Reilly, Vikramjit Mitra, Richard Body, Ajith Kumar Siriwardena, Sreedhar Subramanian, Catrin Plumpton, Dyfrig A Hughes, Tor Mclaren, Amy Lucas, Carrol Gamble, Thomas Jaki, Robert Sutton

**Affiliations:** 1 Liverpool Pancreatitis Research Group, University of Liverpool, Liverpool, UK; 2 West China Centre of Excellence for Pancreatitis, West China Hospital of Medicine, Chengdu, Sichuan, China; 3 Liverpool Clinical Trials Research Centre, University of Liverpool, Liverpool, UK; 4 Post-Doctoral Research Center, The First Affiliated Hospital With Nanjing Medical University, Nanjing, Jiangsu, China; 5 West China Centre of Excellence for Pancreatitis, West China Hospital of Sichuan University, Chengdu, Sichuan, China; 6 Department of Emergency General and Trauma Surgery, Liverpool University Hospitals NHS Foundation Trust, Liverpool, UK; 7 Department of Pancreatobiliary Surgery, Liverpool University Hospitals NHS Foundation Trust, Liverpool, UK; 8 Department of Radiology, Liverpool University Hospitals NHS Foundation Trust, Liverpool, UK; 9 Department of Emergency General and Hepatopancreatobiliary Surgery, Oxford University Hospitals NHS Foundation Trust, Oxford, UK; 10 Emergency Department, Aberdeen Royal Infirmary, Aberdeen, UK; 11 Department of Pancreatobiliary and General Surgery, Leeds Teaching Hospitals NHS Trust, Leeds, UK; 12 Upper GI Surgery, Royal Devon University Healthcare NHS Foundation Trust, Exeter, UK; 13 Emergency Department, University College London Hospitals NHS Foundation Trust, London, UK; 14 Department of Hepatobiliary Surgery, University Hospital of Wales, Cardiff, UK; 15 Department of Gastroenterology, North Tees and Hartlepool NHS Foundation Trust, Hartlepool, UK; 16 Division of Cardiovascular Sciences, The University of Manchester, Manchester, UK; 17 Department of General Surgery, Central Manchester University Hospitals NHS Foundation Trust, Manchester, UK; 18 Department of Gastroenterology, Cambridge University Hospitals NHS Foundation Trust, Cambridge, UK; 19 Centre for Health Economics and Medicines Evaluation, Bangor University, Bangor, Wales, UK; 20 Pancreas Patient Advisory Group, Liverpool University Hospitals NHS Foundation Trust, Liverpool, UK; 21 Liverpool Clinical Trials Research Centre, University of Liverpool, Liverpool, England, UK; 22 Department of Health Data Science, University of Liverpool, Liverpool, UK; 23 Medical Research Council Biostatistics Unit, University of Cambridge, Cambridge, UK; 24 Department of Machine Learning and Data Science, University of Regensburg, Regensburg, Germany

**Keywords:** Pancreatic disease, Inflammation, Randomized Controlled Trial, CLINICAL PHARMACOLOGY

## Abstract

**Introduction:**

Acute pancreatitis (AP) is an increasingly common cause of substantial morbidity and risk of mortality without licensed specific drug therapy. Because tumour necrosis factor α (TNF-α) contributes to AP, anti-TNF therapy may improve outcome from AP. This trial aims to test the efficacy, safety, cost-effectiveness and mechanism of action of the anti-TNF antibody infliximab in patients with AP.

**Methods and analysis:**

Randomised treatment of Acute Pancreatitis with Infliximab: Double-blind, placebo-controlled, multi-centre trial (RAPID-I) is a phase 2b trial designed to randomise 240 patients with AP to receive a single 5 mg/kg or 10 mg/kg infliximab or placebo infusion in a 1:1:1 ratio, initiated within 36 hours of hospital admission. The primary outcome is mean serum C-reactive protein (CRP) on trial days 2, 4 (±1 day) and 14 (±2 days) summated as area under the curve (AUC), with 25% reduction in either active arm compared with placebo considered clinically meaningful. Secondary outcomes include cumulative pain scores, opiate requirements, nutritional deficit (days without solid food), decline in serum albumin (negative AUC), rise in neutrophils (AUC), cumulative selective serial organ failure assessment (SOFA-2) scores, local pancreatic injury on contrast-enhanced CT scan (CECT day 14±7 days), infections, length of stay, mortality and patient reported outcome on days 4 (±1 day), 14 (±2 days) and 90 (±7 days). Cost-effectiveness will be based on costs and quality-adjusted life years over 90 (±7) days. Potential safety signals will be adverse events related to infliximab. Transcriptomic, cytokine and leucocyte profiles will be assessed at baseline and the same time points as CRP. Two adaptive interim analyses are planned.

**Ethics and dissemination:**

The protocol has received Research Ethics Committee approval (South Central – Oxford C Research Ethics Committee 18/SC/0262). The findings will be disseminated through peer reviewed publication, national and international conferences and meetings.

**Trial registration number:**

NCT03684278.

**STRENGTHS AND LIMITATIONS OF THIS STUDY**

- This randomised, double-blind, placebo-controlled, multi-centre trial will test a single infusion of standard or high dose infliximab in the treatment of acute pancreatitis.
- Consenting patients will be stratified by site for randomisation in a 1:1:1 ratio to receive either 5 mg/kg or 10 mg/kg infliximab or an equal volume of 0.9% saline placebo.
- Trial treatment will be started within 36 hours of presentation at, with subsequent admission to hospital, allowing up to 120 min for the preparation of trial medication.
- The primary outcome will be C-reactive protein area under the curve (AUC) on days 2, 4 (±1 day) and 14 (±2 days), with ≥25% smaller AUC indicative of impact.
- The trial includes two adaptive interim analyses that will enable early cessation of one treatment arm or all arms should there be sufficient safety concern or lack of efficacy.

## Introduction

 Acute pancreatitis (AP) typically causes excruciating pain, gastrointestinal dysfunction and systemic inflammatory responses that can induce respiratory, cardiovascular and renal dysfunction, carrying a significant risk of organ failure and death.^1^ As one of the the most common gastrointestinal causes of emergency admission to hospital, the number of patients developing AP has increased in incidence in almost all countries surveyed over the last five decades.^2 3^ Despite multiple randomised clinical trials and systematic reviews, there is no licensed specific therapy with international consensus that ameliorates, let alone aborts, the condition.^4 5^ This absence is likely due to the choice of agent and mechanism targeted, as well as speed of treatment delivery in randomised trials, coupled with a lack of an agreed core outcome set for drug trials in AP.^4 6^ Trial randomisation has often been up to 72 or more hours of admission, late in the treatment of a medical emergency, complicated by inaccurate prognostic indices to select or stratify for predicted severity.^6–8^

Tumour necrosis factor alpha (TNF-α) has a major role in the pathogenesis and progression of AP.^9–11^ TNF-α is released by injured pancreatic acinar cells to initiate immune responses in which it is pivotal,^12^ driving many deleterious inflammatory cascades. The mechanistic basis of TNF-α as a key contributor and potential benefits of anti-TNF treatment in AP have been shown in animal^13–17^ and ex-vivo human^18^ models, with marked amelioration following both genetic and pharmacological inhibition of TNF-α. TNF-α expression-enhancing −1031C and −863A alleles significantly increase the risk of organ dysfunction in patients with AP.^10^ TNF-α rises in human AP for a week or more, proportional to disease severity,^19–22^ and anti-TNF treatment used for other indications has been found to reduce the incidence of AP to less than one tenth of that without anti-TNF treatment.^23^ A single-centre trial of pentoxifylline, which reduces cytokine production, did not show benefit in AP,^24^ suggesting that to test the efficacy of anti-TNF therapy in AP, more potent and specific anti-TNF medication is required. Infliximab, a chimeric, monoclonal, anti-TNF antibody that has proven therapeutic effectiveness in inflammatory bowel disease^25^ and other inflammatory diseases,^26^ binds to TNF-α with high affinity, and following intravenous (i.v.) administration, has a circulating concentration in excess of that of circulating TNF-α concentrations in inflammatory diseases. There is therefore a mechanistic rationale for a trial in AP of infliximab, which can be given to ensure rapid bioavailability. Since there has been no previous randomised trial of infliximab in AP, however, and the size of the TNF-α antigen load in AP is unknown, both a standard dose as used in inflammatory bowel disease (5 mg/kg) and a high dose (10 mg/kg) are proposed.^27–30^

A principal risk to participants is infliximab-related i.v. infusion reactions,^31^ the majority of which occur after repeated administration and are mild, although these can be severe, so this risk should be mitigated. The other significant risk is an increased likelihood of infection,^31^ including reactivation of tuberculosis or chronic viral infection and exacerbation of any bacterial infection consequent on AP. In the context of a disease that can be complicated by infection, and in the absence of prior randomised controlled trials of infliximab in AP, mitigation is appropriate. Further risks from infliximab^31^ can be minimised by exclusion criteria for trial participation.

**Figure 1:**
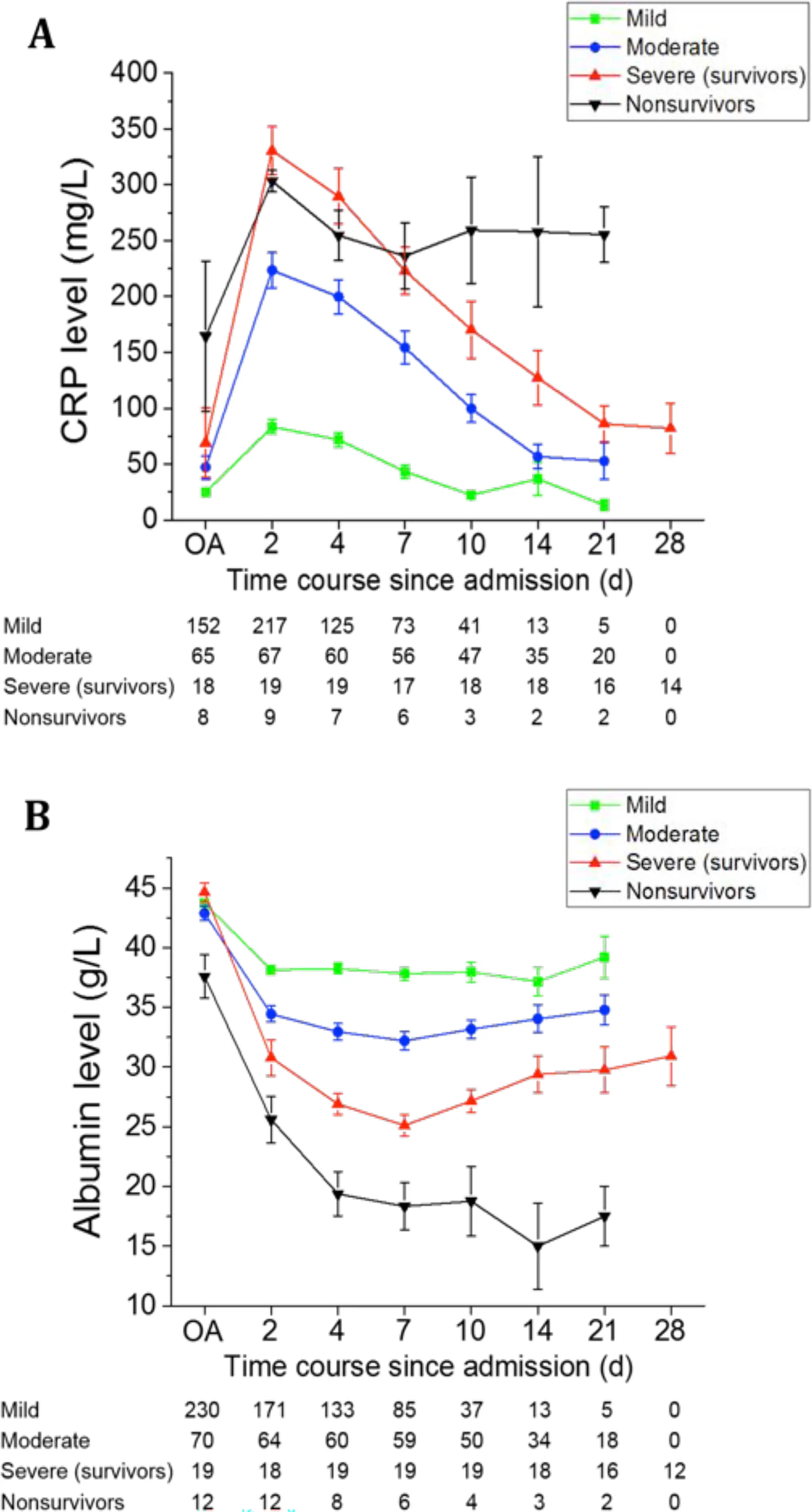
Changes in serum (**A**) C-reactive protein (CRP) and (**B**) albumin levels in 333 patients with either mild, moderate, severe or fatal (12 patients) acute pancreatitis (Revised Atlanta Classification of severity),^60^ showing the number of inpatients with data for each time point. Patients were admitted to the Royal Liverpool University Hospital from 2010 to 2015 with clinical, laboratory, radiological and interventional data recorded in a prospective database. The abbreviation d denotes days.

Whether to restrict enrolment to patients with AP predicted to be more severe and the choice of the primary outcome measure are inter-related trial design considerations.^4 6–8^ Restriction of recruitment to more severe AP is more likely to allow use of major clinical complications in the primary outcome, for example, as a composite of potential events.^32^ Such restriction, however, excludes many patients who may benefit from the drug and is more difficult to undertake, as the potential patient pool is reduced in size. Easing restrictions on participation allows for greater generalisability and wider applicability of results but must have a meaningful primary outcome measure. Elevation of circulating C-reactive protein (CRP) levels occurs in the vast majority if not all patients with AP, with amplitude and period of elevation directly proportional to actual disease severity, including the presence and duration of organ dysfunction (see figure 1). The area under the curve (AUC) of these changes over time captures the clinical course more comprehensively and accurately than a single measure, whether a maximum value or at a specific time.^33^ Furthermore, CRP falls in response to supportive intervention in AP, for example, effective drainage of an infected collection.^34^ CRP is widely used as a robust tool to determine clinical management in AP^1 35^ and other inflammatory diseases,^36^ including in composite scores that have been used for regulatory drug approval.^37–39^ Although CRP AUC would be a surrogate measure of clinical outcome, it has wide applicability to the full range of severities of AP, provided that the level of change required from a trial treatment is large enough to indicate a high likelihood of meaningful clinical impact, and secondary outcome measures are supportive of such an effect. As a primary endpoint surrogate marker, CRP AUC aligns with the SPIRIT-Surrogate criteria in biological plausibility, prognostic value, responsiveness to intervention and consistency across severity strata.^40^ A fall in serum albumin is also reflective of disease course (see figure 1) and may also be used as a surrogate marker.^1 41^

We propose a randomised controlled trial in AP with the primary objective of determining the efficacy of a single infusion of either 5 mg/kg or 10 mg/kg infliximab compared with placebo, reducing door-to-needle time compared with previous drug trials in AP.^6–8^ A single infusion is anticipated to be sufficient as the elimination half-life of infliximab is 7–12 days with a mean residence time of 12–17 days prior to full clearance by the reticulo-endothelial system.^27 42^ Secondary objectives will be to determine the safety and cost-effectiveness of the single infusion of infliximab, to evaluate differential gene expression in trial participants, to develop a clinically meaningful outcome measure that might be used for drug regulatory purposes, and to develop national capacity to undertake trials in pancreatitis.

## Methods and analysis

### Trial design

RAPID-I is a randomised, double-blind, placebo-controlled, multi-centre, three-arm, adaptive, phase 2b efficacy trial of infliximab in patients with AP coordinated by Liverpool Clinical Trials Centre (LCTC). Patients will be stratified by site for randomisation in a 1:1:1 ratio to receive a blinded i.v. infusion of either 5 mg/kg or 10 mg/kg infliximab or an equal volume of 0.9% saline placebo, begun within 36 hours of admission to hospital, allowing up to 120 min for preparation of trial medication. Treatment allocation will only be revealed to pharmacy or unblinded staff preparing trial treatment, to ensure the research teams administering the treatment and assessing participants remain blinded. Standard Protocol Items: Recommendations for Interventional Trials (SPIRIT)^43^ and SPIRIT-Surrogate,^40^ Consolidated Standards of Reporting Trials (CONSORT)^44^ and CONSORT-Surrogate(46),^45^ and Consolidated Health Economic Evaluation Reporting Standards (CHEERS)^46^ guidelines from the EQUATOR Network will be followed throughout.

**Figure 2:**
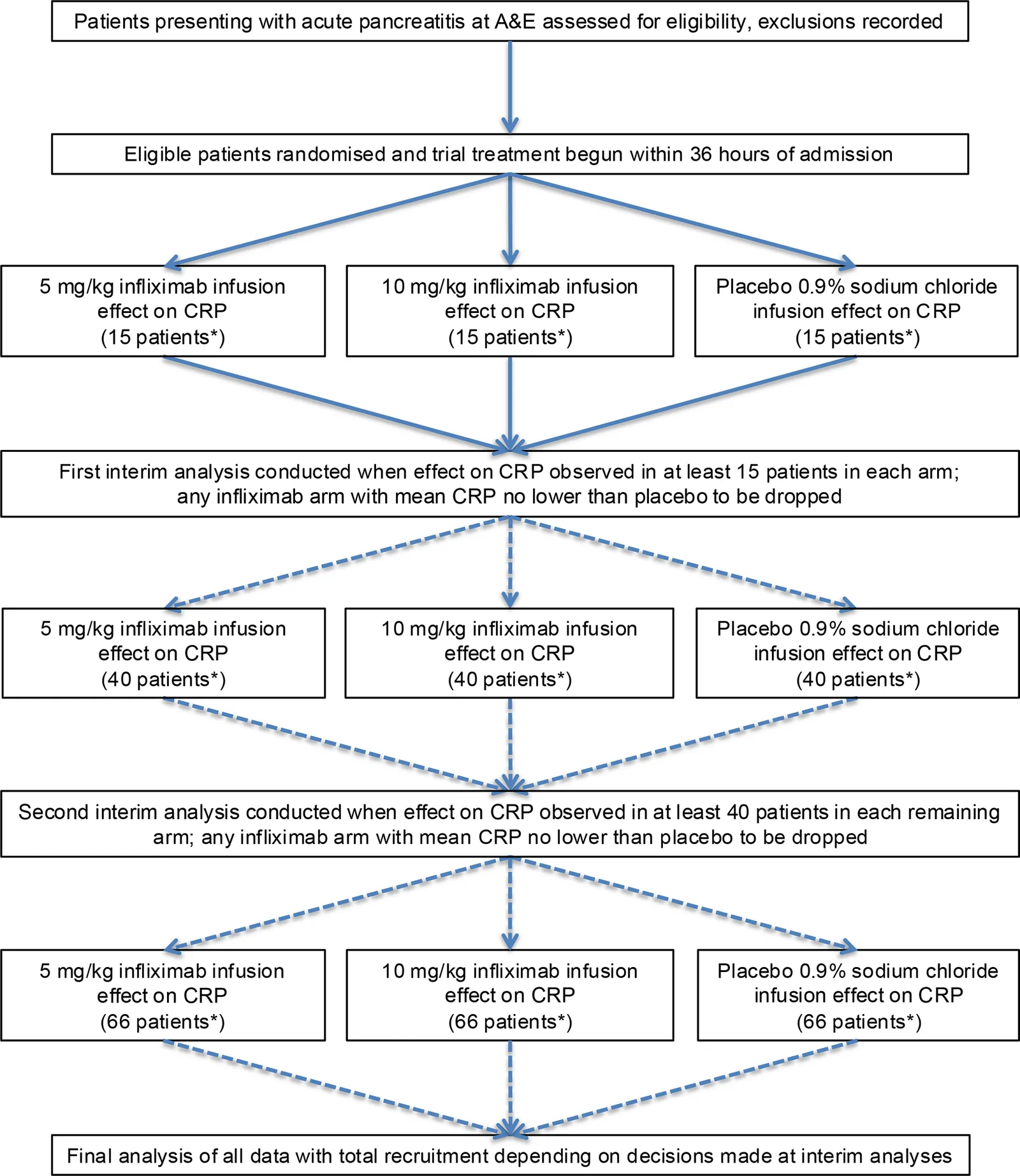
Flow diagram of RAPID-I. *Number of patients where primary outcome has been observed, not number of patients recruited. A&E, Accident and Emerency; CRP, C-reactive protein; RAPID-I, Randomised treatment of Acute Pancreatitis with Infliximab: Double-blind, placebo-controlled, multi-centre trial.

The primary outcome will be CRP AUC from measurements on days 2, 4 (±1 day) and 14 (±2 days), with detection of 25% smaller CRP AUC in either treatment arm compared with placebo considered indicative of a meaningful impact on clinical outcomes in AP.^1 34–36 47^ Patients discharged before these times will be asked to reattend for the measurements (considered likely often for day 14). A last value carried forward strategy will be used to compute the CRP AUC for patients with a missing CRP measurement for day 14 (taken from that from day 4), but not for day 4 (from day 2), as we consider day 4 CRP essential to assessment of the impact of the trial treatment. We calculate a total sample size of 198 patients with an equal number in each trial arm (n=66 with evaluable primary outcome data in each arm) will provide a one-sided overall family-wise type 1 error rate of 2.5% and power of 80% to demonstrate a 25% reduction in CRP AUC. This specification corresponds to a difference between groups of 0.545σ, where σ is the SD of CRP AUC, a difference with the advantage that it is not necessary to pre-specify the SD to obtain the required sample size. RAPID-I will seek to recruit up to an overall total of 240 patients, to account for a 20% drop-out (including missing CRP measurements) rate from each arm, allowing for the relative paucity of drug trials in AP and consequent lack of experience of drop-out rates. Up to three analyses of the efficacy data (two interim analyses and a final analysis) are planned (see figure 2), as a smaller expected sample size may be mandated by using interim analyses, depending on findings. Any treatment arm found at either interim analysis to have a mean CRP AUC value larger or equal to the placebo arm will be dropped, and should both treatment arms be dropped, the trial will stop. To account for (potentially) dropping a dose arm for other reasons, the conditional error principle will be used to adjust the design.^48^ Application of this principle will ensure that the overall type I error rate of the study is still controlled, resulting in an increased power compared to not accounting for an unplanned modification. At the final analysis, comparative t-statistics will be computed using an analysis of covariance (ANCOVA) model. If the resulting test statistic for either (or both) doses falls below a critical value of −2.196, the corresponding null hypothesis of no difference between the active group and placebo can be rejected and superiority of the active dose can be claimed. To assess the sensitivity of this approach, we will also conduct a complete case analysis; a longitudinal model will be used as a core secondary analysis. Sub-group analyses will examine outcomes in mild AP using the Harmless Acute Pancreatitis Score^49^ and in severe AP in those presenting with organ failure; if necessary, outcomes will be analysed in a standardised period prior to cholecystectomy in mild AP.

Secondary efficacy outcomes include cumulative pain scores (numerical rating scale from 0 to 10 daily for the first 14 days),^50^ opiate requirements (morphine equivalents daily for the first 14 days),^51 52^ nutritional deficit (number of days without solid food for the first 14 days),^53^ decline in serum albumin (negative AUC from admission value for the first 14 days),^41^ rise in circulating neutrophils (AUC for the first 14 days)^54^ and cumulative selective serial organ failure assessment (SOFA for the first 14 days, using the updated SOFA-2 score),^55 56^ all while an inpatient but not after discharge; local pancreatic injury on contrast-enhanced computerised tomography (CT on day 14±7 days as an inpatient or outpatient, with blinded modified CT severity index determined by a central radiology panel)^57^; infective complications (within the first 90 days)^1 5 58^; length of hospital stay (within the first 90 days)^1 5^; mortality (within the first 90 days)^3 4^ and patient reported outcome using the EuroQol EQ-5D-5L questionnaire including the visual analogue scale EQ-VAS on day 4 (±1 day), 14 (±2 days) and 90 (±7 days).^59^ Similarly to the primary outcome CRP AUC, serum albumin negative AUC and circulating neutrophils AUC parallel disease severity (Revised Atlanta Classification),^60^ seen in Spearman coefficients of 0.61 (CRP), 0.59 (albumin) and 0.59 (neutrophils) (all p<0.001) over the first 4 weeks in the same study population (see figure 1). Potential safety signals will be adverse events related to infliximab including infusion reactions and delayed serum sickness reactions (within the first 90 days)^31 61^; a further safety signal will be a significant increase in the incidence of infective complications associated with the use of infliximab compared with placebo; assay for antibodies to infliximab will be undertaken on day 14 (±2 days).^27–30^ Exploratory analyses will include transcriptomics, measuring absolute and/or relative expression of 100 selected transcripts (for severity prediction, efficacy and safety assessment) in samples taken at baseline and on days 2, 4 (±1 day) and 14 (±2 days)^62 63^; cytokine profiles (TNF-α, interleukin 2 and 6, interferon-α and -γ) will be assessed at the same timepoints^16 64^; peripheral blood mononuclear subsets will be evaluated (by CyTOF) at baseline and on days 4 (±1 day) and 14 (±2 days)^65^; infliximab levels will be measured on days 4 (±1 day) and day 14 (±2 days).^27–30^

For continuous secondary outcomes, data will be presented as means and SD, medians and IQRs, minima and maxima, and comparative t-statistics will be computed using an ANCOVA model adjusted for recruiting hospital. For binary secondary outcomes, data will be reported in terms of ORs and analysed using a logistic regression model adjusting for recruiting hospital. Secondary count data will be analysed using Poisson regression, accounting for dispersion using a negative binomial model, and/or for zero-inflation if indicated. Ordinal data will be analysed using an appropriate ordinal regression model. All regression models will be adjusted for recruiting hospital. Bioinformatic analyses of transcriptomic, cytokine and leucocyte subset data will be undertaken and applied to differentially expressed genes, adopting an iterative approach informed by outcome, with sub-group efficacy and safety analyses of those positive or not for any differentially expressed gene transcript signature predictive of actual AP severity.^66^

To develop outcome measures for drug trials in AP, discriminant functions of efficacy measures will be assessed across clinical, laboratory, critical care, radiological, infection, length of stay and patient-reported outcome domains.^4 6^

The cost-effectiveness of infliximab will be assessed from the perspective of the NHS. Resource utilisation will be measured by questionnaire at days 14 (±2 days) and 90 (±7 days) and from routinely collected data from Patient-Level Information and Costing Systems. Responses to the EQ-5D-5L (measured on day 4 (1 day), 14 (±2 days) and 90 (±7 days)) will be converted to health utility scores to estimate mean QALYs (quality-adjusted life years), and the incremental cost per QALY gained estimated by trial treatment. The joint uncertainty in costs and QALYs will be addressed through application of bootstrapping and estimation of cost-effectiveness acceptability curves.^67^ Sensitivity analyses will be conducted to test the robustness of findings.

This trial is open to the participation of any UK NHS hospital providing adult Accident and Emergency, General Surgical and Gastroenterology services, the last with expertise in biologic therapies. Principal investigators will be either emergency medicine physicians, gastroenterologists with expertise in biologics, or surgeons or physicians responsible for the management of patients with AP. Centres are expected to work to the International Association of Pancreatology^5^ and NICE (National Institute for Health and Care Excellence) evidence-based guidelines^68^ for the management of AP and must have sufficient research capacity to undertake the trial.

### Study procedures

Patients will be eligible who are admitted to recruiting hospitals with a new diagnosis of AP established by two of the following:

1. Typical continuous upper abdominal pain.
2. Amylase and/or lipase three or more times the upper limit of normal.
3. Characteristic findings on abdominal imaging if undertaken urgently by CT or MRI^4^; and in whom trial treatment can be started within 36 hours of admission, allowing 120 min within the 36-hour period for preparation of trial medication.

Patients will be excluded (items 4 to 20 are for safety reasons relating to infliximab)^31^

1. Who are aged<18 or >85 years.
2. With a bodyweight over 200 kg.
3. Have known previous AP diagnosed within the last 30 days or known chronic pancreatitis (including that identified by CT on or shortly after admission).
4. Have known multiple sclerosis, systemic vasculitis, Guillain-Barré syndrome or other demyelinating disorder.
5. Have known epilepsy.
6. Have known moderate to severe heart failure and/or coronary disease.
7. Have known severe respiratory conditions including cystic fibrosis, severe asthma and severe chronic obstructive pulmonary disease, on home oxygen or home mechanical ventilation.
8. Have jaundice (serum bilirubin>50 µmol/L) or known advanced liver disease.
9. Have known cancer for which chemotherapy and/or radiotherapy has been ongoing or completed in last 6 months before admission.
10. Have known haematological malignancy.
11. Have known cancer requiring palliative care.
12. Have known established infection prior to or suspected infection, including COVID-19, at the time of AP onset.
13. Have a known history of tuberculosis, or household contact with those with tuberculosis or opportunistic infection.
14. Have a known history of infectious hepatitis (unless the patient has recovered from previous hepatitis A infection).
15. Have known rare diseases or inborn errors of metabolism that significantly increase the risk of infections, including severe combined immunodeficiency and homozygous sickle cell disease.
16. Have received a live vaccine, infectious agent or immunosuppressive therapy within the last 1 month.
17. Have known hypersensitivity to infliximab or to inactive components of infliximab or to any murine proteins.
18. Have a known pregnancy or are lactating at admission.
19. Are women of childbearing potential who do not agree to use adequate contraception up to 6 months after trial infusion treatment.
20. Or are a patient who has participated in an investigational medicinal product study within the last 3 months before admission.

Consenting patients will be randomised by a delegated member of the research team using a 24-hour web-based randomisation system maintained by LCTC, with blocks of variable length stratified by recruiting hospital. Treatment allocation will be emailed to the pharmacy or unblinded research team members who will prepare and place the trial medication in a blinding bag, appropriately labelled to maintain blinding, for administration by blinded research staff. In case of failure of the web-based randomisation system, backup randomisation envelopes will be stored in the pharmacy of each recruiting hospital. The weight of each participating patient will be recorded to calculate the appropriate dose needed for that participant. Infliximab (reference product or biosimilar approved by the UK Medicines and Healthcare products Regulatory Agency) will be diluted in 0.9% sodium chloride not to exceed the manufacturer’s maximum concentration for human administration at 4 mg/mL,^31^ requiring a volume of 250 mL for patients weighing up to 100 kg and 500 mL for patients weighing over 100 kg. In all three arms, the trial treatment infusion will be started within 36 hours of admission, and the full volume of the treatment infusion will normally be administered over a period of 2 and not more than 3 hours. All participating patients will receive 100 mg hydrocortisone and 10 mg chlorphenamine i.v. to prevent infusion reactions,^31^ as well as piperacillin/tazobactam 4.5 g i.v. three times a day (or other suitable antibiotic to prevent AP-related infection^1^ that might result from an increased susceptibility because of infliximab administration) for up to 7 days or until discharge, whichever is earlier, with the first dose prior to the trial treatment infusion. All patients including those randomised to placebo will receive these prophylactic medications to ensure any benefit from active trial treatment can be attributed to the active trial treatment and not one or more of the concomitant medications. Although prophylactic antibiotics are not routine in the management of AP,^5^ selective administration only to those receiving active treatment and not placebo would have the further disadvantage of unmasking the blinding.

**Figure 3:**
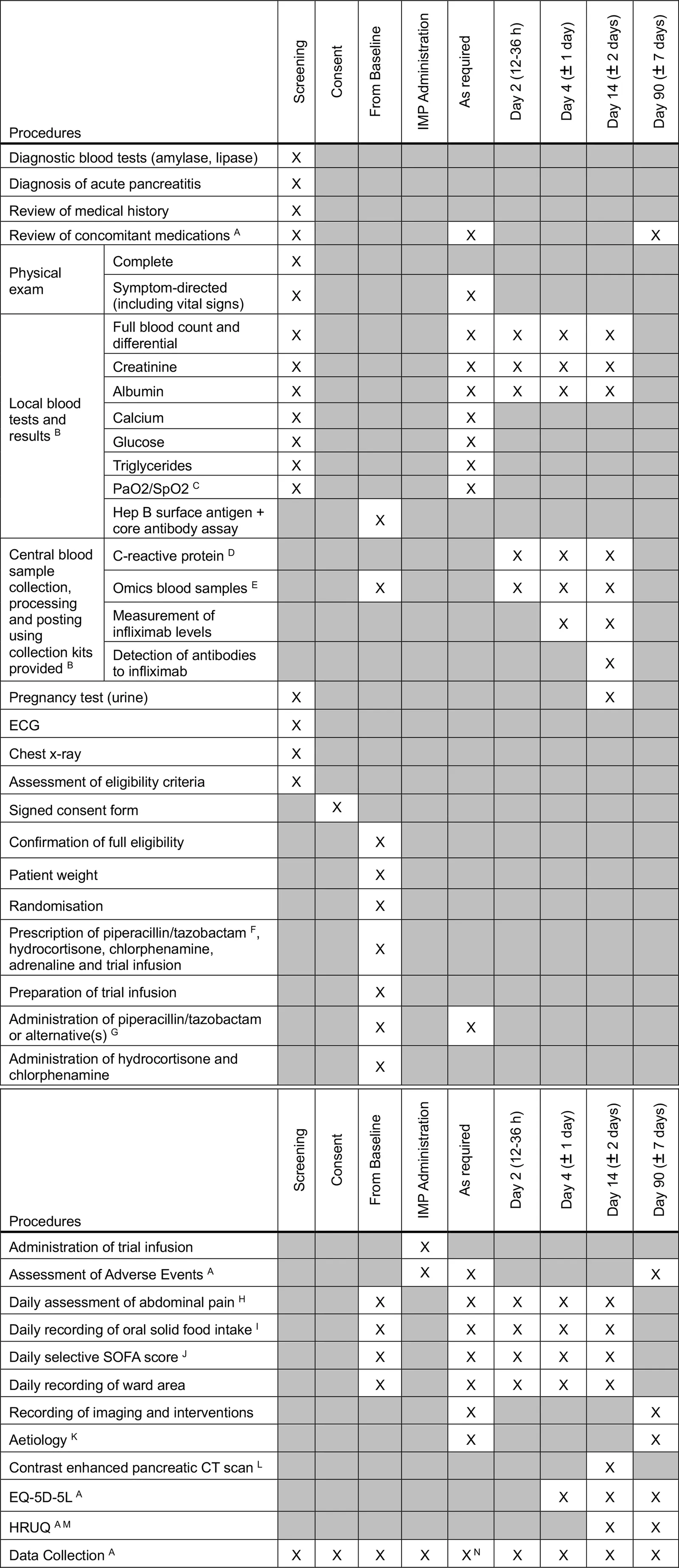
Schedule of assessments for RAPID-I. ^A^Day 90 follow-up can be completed remotely via telephone (including questionnaires). ^B^Consent is mandatory before collection of any blood samples not part of standard care. ^C^ Either pulse oximetry oxygen saturation or arterial blood gases recorded at admission on air. ^D^5 mL clotted blood in SST tube (centrifuged) for CRP ^E^2.5 mL in x 2 PAXgene tubes for RNA; 5 mL in SST tube (centrifuged) for cytokines; at baseline: 8.5 mL in PAXgene tube for DNA; at baseline, Day 4 and 14: 6 mL in ACD tube for PBMCs; volumes subject to availability of tubes of these volumes. ^F^Alternative antibiotics (eg, if penicillin allergy) for intra-abdominal infection as defined in local written antibiotic policy. ^G^Administered for up to 7 days or until discharge, whichever time point is earlier; alternatives given in Section 6.6. ^H^Daily numerical rating scale of abdominal pain and daily administration of opiates during inpatient stay for up to 14 days. ^I^For nutritional assessment see Section 7.1.1, recorded during inpatient stay for up to 14 days. ^J^Cardiac, respiratory and renal only; if no lab test, most recent value from previous day to be used; up to 14 days as^H, I^. ^K^Transabdominal ultrasound, alcohol (AUDIT) and drug history, serum triglycerides, calcium, ±endoscopic ultrasound±magnetic resonance cholangiopancreatography (MRCP). ^L^Contrast enhanced CT scan (CECT) on Day 14±7 days. ^M^Health Resource Use questionnaire (HRUQ, to be completed if patient has been discharged from index (trial) admission). ^N^Data collection on inpatients up to discharge or Day 14, whichever is soonest as for^H, I, J^.

All participants are to be observed for at least 1–2 hours post-infusion for acute infusion-related reactions.^31^ Symptom directed physical examination, nutrition and its route as well as selective SOFA scores will be recorded daily until day 14 or discharge, whichever is sooner. Imaging, therapeutic interventions and ward area will be recorded until day 90 or discharge, whichever occurs sooner. Aetiology will be recorded when available either during the inpatient stay or follow-up period until day 90. Severity classification (Revised Atlanta Classification)^60^ will also be undertaken during this period. The total duration of the study is 90 days from commencement of trial infusion, with assessments as scheduled in figure 3. If patients are discharged before assessment time points are reached, they will be assessed as outpatients. Assessment on day 90 (±7 days) may be undertaken remotely by video link or telephone, if after discharge a participant is unable or would prefer not to return to the research site for their visit.

**Figure 4:**
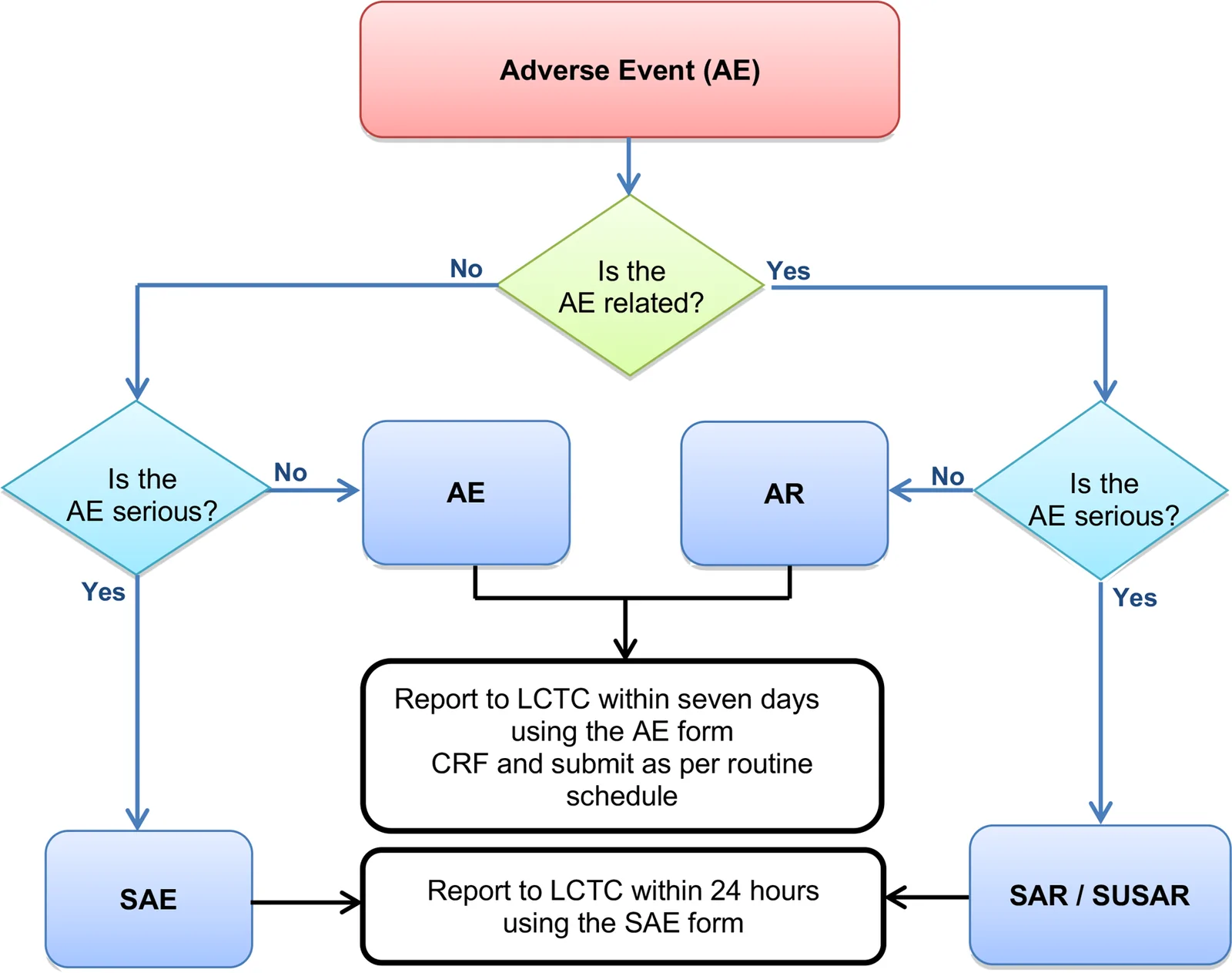
Flowchart for reporting requirements of adverse events (AEs). AE = adverse event; AR = adverse reaction; LCTC = Liverpool Clinical Trials Centre; SAE = serious adverse event; SAR = serious adverse reaction; SUSAR = suspected unexpected serious adverse reaction

The collection, verification and reporting of safety events are to comply with the Medicines for Human Use (Clinical Trials) Regulations 2004,^69^ and as amended by the Medicines for Human Use (Clinical Trials) (Amendment) Regulations 2025 that came into force on 28 April 2026.^70^ All serious adverse events, including serious adverse reactions and suspected unexpected serious adverse reactions, are to be reported to LCTC within 24 hours of the local site becoming aware of the event (see figure 4). Investigators and delegated members of the local research teams will actively monitor participants for all adverse events from randomisation until the end of their trial follow-up period (ie, day 90 time point), and report these to the LCTC. All non-serious adverse events, whether expected or not, are to be recorded and transmitted to the LCTC within 7 days of the local research team becoming aware of the event. Widely recognised and documented clinical features of AP that are defined outcome measures in this trial will not be recorded as adverse events but will be evaluated in comparisons between the three trial arms.

### Patient and public involvement

The aim of patient and public involvement (PPI) in the trial is to make the trial as relevant, meaningful and beneficial to patients as possible. PPI representatives from the Liverpool University Hospitals NHS Foundation Trust Pancreas Patient Advisory Group (whose members have or had pancreatic exocrine disease) contributed to writing the application for funding of the proposed trial from the Efficacy and Mechanism Evaluation (MRC and NIHR) Programme (awarded as 15/20/01 with a decision to fund on 20 February 2017), including as one of the applicants. PPI representatives contributed to trial design through joint strategic oversight of clinical AP data collection undertaken by the NIHR Liverpool Pancreas Biomedical Research Unit (see figure 1), as well as review of the protocol, trial objectives, patient consenting and outcome assessments. PPI representatives are standing members of the Trial Management Group and Steering Committee. RAPID-I endeavours to address, at least in part, 6 of the top 10 research priorities for pancreatitis set by patients, carers and healthcare professionals contributing to the James Lind Alliance Pancreatitis Priority Setting Partnership established in 2021.^71^

### Ethics and dissemination

A favourable opinion on the proposed trial was given by the South Central Oxford C Research Ethics Committee on 4 July 2018 (18/SC/0262) and approved by the Medicines and Healthcare products Regulatory Agency on 13 August 2018 and the Health Research Authority and Health and Care Research Wales on 15 August 2018. The trial is registered in EudraCT (2017-003 840-19), ClinicalTrials.gov (NCT03684278) and the International Standard Randomised Controlled Trial Number Registry (16935761). The current version of the approved protocol, including all amendments and their rationale, is V.16.0 (online supplemental material).

The trial is promoted by its stakeholders, including the UK Clinical Research Networks, investigators, and contributing parties in the Pancreatic Society of Great Britain and Ireland, the British Society of Gastroenterology, European Pancreatic Club, American Pancreatic Association and International Association of Pancreatology, through presentations, lectures, symposia and display stands. If RAPID-I shows meaningful clinical benefit from infliximab in patients with AP, and has promise of being cost-effective, a pivotal phase 3 trial will be planned, as the primary objective of RAPID-I is to develop an approved drug for this disease.

A further objective is to build capacity to undertake multi-centre drug trials in AP, with RAPID-I as the first trial of a UK Pancreatitis Study Group. Progress in opening centres, their recruitment, and the overall time to recruitment of the target sample size will help inform the development of national trial capacity in AP. In addition, the development of a core outcome set for such trials using data from RAPID-I is anticipated to facilitate advances in this field, currently lacking for pharmacological trials in AP.^4 6^ If RAPID-I validates a proposed transcriptomic signature predictive of disease severity or identifies a potential signature indicative of treatment response, these and other findings will be disseminated through presentations to learned societies, publications and patient networks.

All trial documents will be retained for a maximum period of 25 years from the end of the trial, with the principal investigators at each investigational site arranging to store essential trial documents until the LCTC determines that this is no longer necessary. All electronic case report forms and trial data will be archived onto appropriate media for long-term accessible storage. Hard copies of data will be boxed and transferred to secure premises where unique reference numbers will be applied for confidentiality, tracking and retrieval. All residual patient samples for which there is consent and not required for analyses specified in the protocol will be transferred from the UK Biocentre to the University of Liverpool Acute Pancreatitis Research Biobank and maintained at −80 °C for up to 10 years for future medical research.

## Discussion

Randomised controlled trials of drugs for AP date back to the 1960s,^72^ yet the need for an internationally approved first-line therapy for AP remains unmet. The hope underpinning RAPID-I is that anti-TNF therapy will meet this need, demonstrating efficacy that would justify subsequent definitive assessment in a pivotal phase 3 trial. Whatever the trial outcome, experience with RAPID-I will build capacity for future trials of other drugs in AP. The strategy to approach an intended market authorisation could either be to include all predicted severities, as in RAPID-I and potential follow-on trials, or to focus on some prediction of severity and conduct phase 4 trials aiming to expand any licence should marketing authorisation be obtained. The reasons for either approach may include mechanism of action, early trial experience, logistics (including required sample sizes and recruitment challenges), regulatory input and commercial considerations. In either case, convincing justification and acceptable design are mandatory, which this protocol seeks to provide, including through exploratory mechanistic evaluation. In RAPID-I, trial treatment will be administered as early as is feasible, unlike in many previous trials of drugs in AP.^7 8^ Since anti-TNF therapy may have wide applicability in AP, participation is not restricted to predicted severe disease, enhancing potential recruitment and the generalisability of results. For efficiency in trial design, a meaningful surrogate primary outcome measure (serum CRP AUC on days 2, 4 and 14) with read-out for every patient is proposed. The use of prophylactic antibiotics could limit generalisability, but we considered them necessary for safety in this first phase 2 trial of infliximab in AP. If subsequent trials of infliximab are conducted in AP, these could test or even drop prophylactic antibiotics, as in CATALYST and ACTIV-1 in COVID-19 pneumonia.^73 74^

Delivery of RAPID-I is critically dependent on UK Clinical Research Network research staff at each participating centre, with recruitment and administration of trial medication largely restricted to standard daytime working hours. Some centres have declined participation because of a lack of capacity, or having been initiated, have been unable to recruit because of inability to draw together and/or maintain sufficient research capacity, and closed. Initially the protocol (V.6.0 approved 18 December 2018) required infusion of trial medication to begin within 12 hours of hospital admission to minimise door to needle time, but informed by screening logs, this proved impractical for recruitment and was replaced by infusion of trial medication to begin within 36 hours of the onset of abdominal pain (V.7.0 approved 18 July 2019). This also was unsatisfactory, as some patients could not recall when their pain started, or complained of vague prodromes during which time amylase levels were normal, but subsequently developed acute, severe abdominal pain, accompanied by diagnostic amylase levels. Recruitment was suspended during the COVID-19 pandemic, after which the time of onset of abdominal pain was replaced by the requirement for trial medication to begin within 36 hours of admission to hospital, balancing door to needle time against deliverability (V.12.0 approved 14 August 2023). On 22 April 2026 the Independent Data and Safety Monitoring Committee reviewed the second interim analysis and recommended dropping the 10 mg/kg arm, owing to lack of efficacy. The Trial Steering Committee approved this on 11 May 2026, so this arm was dropped on 19 May 2026. As of 30 June 2026 (V.16.0 approved 14 April 2026), 215 patients have been recruited at nine UK centres, and is ongoing.

## Supplementary material

10.1136/bmjopen-2026-118729 online supplemental file 1
