# Supplementary material for: Randomised treatment of acute pancreatitis with infliximab: protocol for a double-blind, placebo-controlled, multi-centre, adaptive, phase 2 superiority trial (RAPID-I)
Source: BMJ Open. 2026 Sep 18;16(9):e118729. doi: 10.1136/bmjopen-2026-118729 (PMC13599857; doi:10.1136/bmjopen-2026-118729)
Supplement: Supplementary file 1 [file bmjopen-2026-118729.reviewer_comments.pdf]

PEER REVIEW HISTORY

BMJ Open publishes all reviews undertaken for accepted manuscripts. Reviewers are asked to complete a checklist review form and are provided with free text boxes to elaborate on their assessment. These free text comments are reproduced below.

ARTICLE DETAILS

Title (Provisional)

Randomised treatment of Acute Pancreatitis with Infliximab: protocol for a Double-blind, placebo-controlled, multi-centre, adaptive, phase 2 superiority trial (RAPID-I)

Authors

Lin, Juan; Smyth, Matt; Spowart, Catherine; Brown, Michaela; Powell, Ian; Latawiec, Diane; Wu, Di; Huang, Wei; Mukherjee, Rajarshi; Szatmary, Peter; Baron, Ryan; Dunne, Declan; Evans, Jonathan; Srinivas, Sidharth; Bond- Smith, Giles; Cooper, Jamie; smith, andrew; Manzelli, Antonio; Elkhodair, Samer; O'Reilly, David; Mitra, Vikramjit; Body, Richard; Siriwardena, Ajith Kumar; Subramanian, Sreedhar; Plumpton, Catrin; Hughes, Dyfrig A; Mclaren, Tor; Lucas, Amy; Gamble, Carrol; Jaki, T; Sutton, Robert

---

VERSION 1 - REVIEW

**Reviewer**	**1**
**Name**	**Sakai, Arata**
**Affiliation**	**Kobe University**
**Date**	**11-Jun-2026**
**COI**	**None**

This is a well-written and clinically relevant protocol for a multicentre, double-blind, placebo-controlled, adaptive phase 2b trial evaluating infliximab in acute pancreatitis. The rationale is clearly presented, the unmet clinical need is well described, and the trial design is generally appropriate for an early efficacy and mechanistic study. I do not identify any fundamental methodological concerns that would preclude publication as a study protocol. The choice of CRP AUC as a surrogate primary endpoint is justified in the manuscript, and the authors appropriately acknowledge its limitations. I have only minor comments for clarification:

1. The manuscript would benefit from a slightly clearer explanation of how missing CRP values will be handled in practice, particularly given the use of last observation carried forward and the possibility of early discharge.

2. The rationale for universal prophylactic antibiotic administration, including in the placebo arm, is understandable from the standpoint of blinding and safety. However, because

prophylactic antibiotics are not routine standard care in acute pancreatitis, the authors may wish to further clarify how this could affect generalisability and interpretation of infection-related secondary outcomes.

3. The discussion appropriately notes that the treatment window was amended during trial conduct. It would be helpful to ensure that the timing of protocol amendments, recruitment status, and the current approved protocol version are clearly and consistently reported.

VERSION 1 - AUTHOR RESPONSE

Reviewer 1: This is a well-written and clinically relevant protocol for a multicentre, double-blind, placebo-controlled, adaptive phase 2b trial evaluating infliximab in acute pancreatitis. The rationale is clearly presented, the unmet clinical need is well described, and the trial design is generally appropriate for an early efficacy and mechanistic study. I do not identify any fundamental methodological concerns that would preclude publication as a study protocol. The choice of CRP AUC as a surrogate primary endpoint is justified in the manuscript, and the authors appropriately acknowledge its limitations. I have only minor comments for clarification:

Response: we thank the reviewer for stating our protocol is well written and clinically relevant.

Reviewer 1: The manuscript would benefit from a slightly clearer explanation of how missing CRP values will be handled in practice, particularly given the use of last observation carried forward and the possibility of early discharge.

Response: how missing CRP values will be handled has been clarified in the methods, specifying which values will be carried forward and which will not.

Reviewer 1: The rationale for universal prophylactic antibiotic administration, including in the placebo arm, is understandable from the standpoint of blinding and safety. However, because prophylactic antibiotics are not routine standard care in acute pancreatitis, the authors may wish to further clarify how this could affect generalisability and interpretation of infection-related secondary outcomes.

Response: the rationale for prophylactic antibiotics in all trial patients has been elaborated in the discussion with clarification as to how this could affect generalisability and interpretation of infection-related secondary outcomes.

Reviewer 1: The discussion appropriately notes that the treatment window was amended during trial conduct. It would be helpful to ensure that the timing of protocol amendments, recruitment status, and the current approved protocol version are clearly and consistently reported.

Response: the sequence of protocol amendments, recruitment status and current approved protocol version are now clearly and consistently reported in the discussion.

In addition, three additional references support these amendments, and one author who contributed to trial design has been added, having approved the revised manuscript.

We hope our revised manuscript is now acceptable for BMJ Open.

VERSION 2 - REVIEW

**Reviewer**	**1**
**Name**	**Sakai, Arata**
**Affiliation**	**Kobe University**
**Date**	**31-Aug-2026**
**COI**	

The points I raised have been appropriately addressed.
