# Supplementary material for: Randomised treatment of acute pancreatitis with infliximab: protocol for a double-blind, placebo-controlled, multi-centre, adaptive, phase 2 superiority trial (RAPID-I)
Source: BMJ Open. 2026 Sep 18;16(9):e118729. doi: 10.1136/bmjopen-2026-118729 (PMC13599857; doi:10.1136/bmjopen-2026-118729)
Supplement: Supplementary file 2 [file bmjopen-16-9-s001.docx]

**Phase IIb, randomised, double-blind, placebo-controlled, multi-centre trial of infliximab with transcriptomic biomarker and mechanism evaluation in patients with acute pancreatitis.**

**Protocol 16.0 24/02/2026**

| **Study Sponsor:**  **University of Liverpool**  **Liverpool**  **L69 3BX**  **Tel: 0151 795 1780**  **Email:** [**sponsor@liverpool.ac.uk**](mailto:sponsor@liverpool.ac.uk) | **EudraCT number: 2017-003840-19**  **CTA Reference Number**: **04196/0049/001-0001**  **NCT Number: NCT03684278**    **Research Ethics Ref: 18/SC/0262**  **Sponsor Ref: UoL001326**  **Funder Ref: 15/20/01**  **IRAS Number:** **207163**  **ISRCTN: 16935761** |
| --- | --- |

This study (Ref: 15/20/01) is funded by the Efficacy and Mechanism Evaluation (EME) Programme, an MRC and NIHR partnership. The views expressed in this publication are those of the author(s) and not necessarily those of the MRC, NIHR or the Department of Health and Social Care.

Protocol Approval

I, the undersigned, hereby approve this clinical study protocol:

Protocol Approval

I, the undersigned, hereby approve this clinical study protocol:

Protocol Approval

I, the undersigned, hereby approve this clinical study protocol:

**General Information**

This document describes the RAPID-I trial including detailed information about procedures and recruitment. The protocol should not be used as an aide-memoir or guide for the treatment of other patients; every care was taken in its drafting, but corrections or amendments may be necessary. This will be circulated to the registered investigators in the trial, but centres entering patients for the first time are advised to contact the coordinating centre, the Liverpool Clinical Trials Centre (LCTC), to confirm they have the most up-to-date version. Clinical problems relating to this trial should be referred to the CI, Professor Robert Sutton, via the LCTC.

This protocol defines the participant characteristics required for study entry and the schedule of treatment and follow-up. Participant recruitment will be undertaken in compliance with this document and applicable regulatory and governance requirements; waivers to authorise non-compliance are not permitted.

Protocol non-compliance, whether reported prospectively (e.g. where an investigation cannot be undertaken on a scheduled date because of a clinical contraindication) or retrospectively noted (e.g. as a result of central monitoring) is recorded as protocol deviation, the incidence of which is monitored and reported to trial oversight committees.

The template content structure is consistent with Standard Protocol Items: Recommendations for Interventional Trials 2013 (SPIRIT) and has regard for the Health Research Authority (HRA) guidance. Regulatory and ethical compliance information is located in Section 11.

**Relationship Statements**

Roles and responsibilities are fully described in Section 14.

The University of Liverpool is the sponsoring organisation and will formally delegate specific sponsoring roles to the CI and Clinical Trials Unit, while remaining legally responsible for the trial. The clinical management and governance of all patients screened and/or entered into RAPID-I including identification of patients who lack capacity is the responsibility of each recruiting hospital NHS Trust.

Clinical Trials Unit (CTU): the LCTC at the University of Liverpool in collaboration with the CI, Professor Robert Sutton, will have overall management responsibility for the trial and will be responsible for the co-ordination of centres.

The Liverpool Clinical Trials Centre brings together a wealth of expertise built on the experience of the Liverpool Trials Collaborative which has held full registration status with the UK Clinical Research Collaboration Clinical Trials Unit (UKCRC CTU) network since its establishment in 2007 ([www.ukcrc.org](http://www.ukcrc.org)).

| **Contact Details: Institutions** | | |
| --- | --- | --- |
| **Sponsor (University of Liverpool):** | **Trial Management, Monitoring and Analysis:** | **Trial Contracts and Finance:** |
| Clinical Directorate  4th Floor Thompson Yates Building  Faculty of Health and Life Sciences  University of Liverpool  Liverpool L69 3GB  Telephone: 0151 795 1780  Email: [sponsor@liverpool.ac.uk](mailto:sponsor@liverpool.ac.uk) | Liverpool Clinical Trials Centre  University of Liverpool  2^nd^ Floor Institute in the Park  Alder Hey Children's NHS Foundation Trust  Liverpool  L12 2AP  Telephone: 0151 794 9774  E-mail: [rapid.one@liverpool.ac.uk](mailto:rapid.one@liverpool.ac.uk) | Liverpool Health Partners Joint Research Office  Liverpool Science Park  131 Mount Pleasant  Liverpool  L3 5TF  Telephone: 0151 282 6652  E-mail: [contact@lhpjro.nhs.uk](mailto:contact@lhpjro.nhs.uk) |
| **General Laboratory Contact:** | **Other Medical or Technical Departments and/or Institutions** (Trial sample processing & storage)**:** | **Health Economics:** |
| Institute of Systems, Molecular and Integrative Biology  2rd Floor Sherrington Building  University of Liverpool  Ashton Street  Liverpool  L69 3GE  Telephone: 0151 794 9951  E-mail: [latawiec@liverpool.ac.uk](mailto:latawiec@liverpool.ac.uk) | UK Biocentre  Units 2 and 3  Java Park  Bradbourne Drive  Tilbrook  MK7 8AT  Telephone: 0190 887 0800  E-mail: [jess.bailey@ukbiocentre.com](mailto:jess.bailey@ukbiocentre.com) | Centre for Health Economics and Medicines Evaluation  Bangor University  Holyhead Road  Bangor  Gwynedd  North Wales  LL57 2PZ  Telephone: 01248 382950  Email: [d.a.hughes@bangor.ac.uk](mailto:d.a.hughes@bangor.ac.uk) |

| **Contact Details: Individuals** | | |
| --- | --- | --- |
| **Individual Authorised to Sign the Protocol and Protocol Amendments on behalf of the Sponsor:** | | **Chief Investigator:** |
| Mrs Karen Jennings-Wilding  Senior Clinical Research Governance Manager  Clinical Directorate  4th Floor Thompson Yates Building  Faculty of Health and Life Sciences  University of Liverpool  Liverpool L69 3GL  Telephone: 0151 795 1780  E-mail: [sponsor@liverpool.ac.uk](mailto:sponsor@liverpool.ac.uk) | | Professor Robert Sutton  Institute of Systems, Molecular and Integrative Biology  University of Liverpool  2^nd^ Floor Sherrington Building  Ashton Street  Liverpool  L69 3GE  Telephone: 0151 795 8024  E-mail: [r.sutton@liverpool.ac.uk](mailto:r.sutton@liverpool.ac.uk) |
| **Medical Expert who will Advise on Protocol Related Clinical Queries (if other than CI):** | | **Medical Expert who will Evaluate SAE Reports (to be evaluated by CI above):** |
| Dr Sreedhar Subramanian  Consultant Gastroenterologist  Gastroenterology Department  Cambridge University Hospitals NHS Foundation Trust  Hills Road  Cambridge  CB2 0QQ  Telephone: 01223 217452  E-mail:  sreedhar.subramanian2@nhs.net | | Dr Sreedhar Subramanian  Consultant Gastroenterologist  Gastroenterology Department  Cambridge University Hospitals NHS Foundation Trust  Hills Road  Cambridge  CB2 0QQ  Telephone: 01223 217452  E-mail:  [sreedhar.subramanian2@nhs.net](mailto:sreedhar.subramanian2@nhs.net) |
|  |  |  |

The contact details of other individuals involved in the trial are detailed in documents supplementary to the protocol and stored in the Trial Master File:

| **Committee/Group** | **Document Title** |
| --- | --- |
| Trial Management Group (TMG)  Trial Steering Committee (TSC)  Independent Data and Safety Monitoring Committee (IDSMC) | RAPID-I Trial Oversight Committee Membership |

Table of Contents

[Protocol Approval	2](#_Toc231996543)

[Protocol Approval	3](#_Toc231996544)

[Protocol Approval	4](#_Toc231996545)

[Table of Contents	9](#_Toc231996546)

[Glossary	13](#_Toc231996547)

[1	Protocol Summary	15](#_Toc231996548)

[Schematic of Study Design	18](#_Toc231996549)

[2	Introduction	19](#_Toc231996550)

[2.1	Background	19](#_Toc231996551)

[2.2	Rationale	19](#_Toc231996552)

[2.2.1	Benefits and Risks	22](#_Toc231996554)

[2.3	Objectives	24](#_Toc231996553)

[2.3.1	Primary Objective	24](#_Toc231996554)

[2.3.2	Secondary Objectives	24](#_Toc231996555)

[2.4	Outcome Measures/Endpoints	24](#_Toc231996556)

[2.4.1	Primary Outcome/Endpoint	24](#_Toc231996557)

[2.4.2	Secondary Outcomes/Endpoints	24](#_Toc231996558)

[3	Trial Design	26](#_Toc231996559)

[4	Study Setting and Selection of Centres/Clinicians	27](#_Toc231996561)

[4.1	Selection of Centres/Clinicians	27](#_Toc231996562)

[4.1.1	Criteria for Patient Recruitment and Data Collection Centres	27](#_Toc231996563)

[4.1.2	Criteria for Data Collection Only Centres	28](#_Toc231996564)

[5	Study Population	29](#_Toc231996565)

[5.1	Inclusion Criteria	29](#_Toc231996566)

[5.2	Exclusion Criteria	29](#_Toc231996567)

[5.3	Co-enrolment Guidelines	31](#_Toc231996568)

[6	Recruitment and Randomisation	32](#_Toc231996569)

[6.1	Participant Identification and Screening	32](#_Toc231996570)

[6.2	Informed Consent	32](#_Toc231996571)

[6.2.1	Obtaining Informed Consent	33](#_Toc231996572)

[6.2.2	Patients Lacking Capacity	34](#_Toc231996573)

[6.2.3	Patients and/or Legal Representatives Unable to Read	35](#_Toc231996574)

[6.2.4	Ongoing Informed Consent	35](#_Toc231996575)

[6.3	Eligibility Confirmation	36](#_Toc231996576)

[6.4	Baseline	36](#_Toc231996577)

[6.5	Randomisation Procedures	37](#_Toc231996578)

[6.6	Who is Blinded to Allocations	38](#_Toc231996579)

[7	Participant Timeline, Procedures and Assessments	39](#_Toc231996580)

[7.1	Schedule for Follow-up	39](#_Toc231996581)

[7.1.1	Continuous Recordings	41](#_Toc231996582)

[7.1.2	Day 2 (12-36 h after commencement of trial infusion)	41](#_Toc231996583)

[7.1.3	Day 4 (+/- 1 day i.e. 48-120 h after commencement of trial infusion)	42](#_Toc231996584)

[7.1.4	Day 14 (+/-2 days)	42](#_Toc231996585)

[7.1.5	Discharge	42](#_Toc231996586)

[7.1.6	Day 90 (+/-7 days)	42](#_Toc231996587)

[7.1.7	Beyond Day 90	42](#_Toc231996588)

[7.2	Efficacy	42](#_Toc231996589)

[7.3	Procedures for Assessing Safety	43](#_Toc231996590)

[7.3.1	Safety Assessment 1	44](#_Toc231996591)

[7.3.2	Safety Assessment 2	44](#_Toc231996592)

[7.3.3	Safety Assessment 3	44](#_Toc231996593)

[7.3.4	Safety Assessment 4	44](#_Toc231996594)

[7.3.5	Safety Assessment 5	44](#_Toc231996595)

[7.3.6	Safety Assessment 6	44](#_Toc231996596)

[7.4	Other Assessments	45](#_Toc231996597)

[7.4.1	Health Economics	45](#_Toc231996598)

[7.4.2	Special Assays or Procedures	45](#_Toc231996599)

[7.5	Patient Transfers	46](#_Toc231996600)

[7.6	Withdrawal	46](#_Toc231996601)

[7.6.1	Premature Discontinuation of Trial Intervention	46](#_Toc231996602)

[7.6.2	Withdrawal from Trial Completely	47](#_Toc231996603)

[7.7	Loss to Follow-up	47](#_Toc231996604)

[7.8 Trial Closure	47](#_Toc231996605)

[7.8.1	Definition of End of Trial	47](#_Toc231996606)

[8	Trial Treatment	48](#_Toc231996607)

[8.1	Introduction	48](#_Toc231996608)

[8.2	Arm A - Infusion of 5 mg/kg Infliximab	48](#_Toc231996609)

[8.2.1	Formulation, Packaging, Labelling, Storage and Stability	48](#_Toc231996610)

[8.2.2	Dosage, Preparation and Administration of Study Treatment/s	49](#_Toc231996611)

[8.2.3	Specific Restrictions	49](#_Toc231996612)

[8.2.4	Overdose	49](#_Toc231996613)

[8.3	Arm B – Infusion of 10 mg/kg Infliximab	50](#_Toc231996614)

[8.3.1	Formulation, Packaging, Labelling, Storage and Stability	50](#_Toc231996615)

[8.3.2	Dosage, Preparation and Administration of Study Treatment/s	50](#_Toc231996616)

[8.3.3	Specific Restrictions	50](#_Toc231996617)

[8.3.4	Overdose	50](#_Toc231996618)

[8.4	Arm C – Placebo	50](#_Toc231996619)

[8.5	Dose Modifications/Interruption	50](#_Toc231996620)

[8.6	Blinding and Unblinding	51](#_Toc231996621)

[8.6.1	Blinding	51](#_Toc231996622)

[8.6.2	Unblinding	51](#_Toc231996623)

[8.7	Accountability Procedures for Study Treatment	52](#_Toc231996624)

[8.8	Assessment of Compliance with Study Treatment	52](#_Toc231996625)

[8.9	Concomitant Medications/Treatments	53](#_Toc231996626)

[8.9.1	Non-Investigational Medicinal Products (NIMPs)	53](#_Toc231996627)

[8.9.2	Medications Not Permitted/ Precautions Required	53](#_Toc231996628)

[8.9.3	Data on Concomitant Medication	53](#_Toc231996629)

[9	Safety Reporting	54](#_Toc231996630)

[9.1	Terms and Definitions	54](#_Toc231996631)

[9.2	Notes on Adverse Event Inclusions and Exclusions	55](#_Toc231996632)

[9.2.1	Include	55](#_Toc231996633)

[9.2.2	Do Not Include	55](#_Toc231996634)

[9.2.3	Notification of Deaths	56](#_Toc231996635)

[9.2.4	Reporting of Pregnancy / Lactation	56](#_Toc231996636)

[9.3	Notes on Severity / Grading of Adverse Events	57](#_Toc231996637)

[9.4	Relationship to Trial Treatment	57](#_Toc231996638)

[9.5	Expectedness	58](#_Toc231996639)

[9.6	Follow-up After Adverse Events	58](#_Toc231996640)

[9.7	Time Period for Adverse Event Reporting	58](#_Toc231996641)

[9.8	Reporting Procedures	58](#_Toc231996642)

[9.8.1	Non-serious Adverse Events (AEs/ARs)	59](#_Toc231996643)

[9.8.2	Serious Adverse Events (SAEs/SARs/SUSARs)	59](#_Toc231996644)

[9.9	Safety Reporting Responsibilities – Investigator	60](#_Toc231996645)

[9.9.1	Maintenance of Blinding at Sites	61](#_Toc231996646)

[9.10	Safety Reporting Responsibilities – LCTC	61](#_Toc231996647)

[9.10.1	Maintenance of Blinding at LCTC	62](#_Toc231996648)

[9.11	Safety Reports	62](#_Toc231996649)

[9.12	Urgent Safety Measures	62](#_Toc231996650)

[9.13	Contact Details and Out-of-hours Medical Cover	63](#_Toc231996651)

[10	Statistical Considerations	64](#_Toc231996652)

[10.1	Introduction	64](#_Toc231996653)

[10.2	Method of Randomisation	64](#_Toc231996654)

[10.3	Sample Size Calculation	64](#_Toc231996655)

[10.4	Interim Monitoring and Analyses	64](#_Toc231996656)

[10.5	Analysis Plan	65](#_Toc231996657)

[10.6	Health Economic Analysis	66](#_Toc231996658)

[11	Regulatory and Ethical Approvals	67](#_Toc231996659)

[11.1	Statement of Compliance	67](#_Toc231996660)

[11.2	Regulatory Approval	67](#_Toc231996661)

[11.3	Ethical Considerations	67](#_Toc231996662)

[11.4	Approvals	68](#_Toc231996663)

[11.5	Protocol Deviation and Serious Breaches	68](#_Toc231996664)

[12	Data Management and Trial Monitoring	70](#_Toc231996665)

[12.1	Source Documents	70](#_Toc231996666)

[12.2	Data Capture Methods	70](#_Toc231996667)

[12.3	Monitoring	71](#_Toc231996668)

[12.3.1	Central Monitoring	71](#_Toc231996669)

[12.3.2	Clinical Site Monitoring	71](#_Toc231996670)

[12.4	Confidentiality	71](#_Toc231996671)

[12.5	Quality Assurance (QA) and Quality Control (QC)	72](#_Toc231996672)

[12.6	Records Retention	72](#_Toc231996673)

[13	Indemnity	74](#_Toc231996674)

[14	Roles and Responsibilities	75](#_Toc231996675)

[14.1	Role of Study Sponsor and Study Funder	75](#_Toc231996676)

[14.2	Funding and Support in Kind	75](#_Toc231996677)

[14.3	Protocol Contributors	75](#_Toc231996678)

[14.4	Trial Committees	76](#_Toc231996679)

[14.4.1	Trial Management Group (TMG)	76](#_Toc231996680)

[14.4.2	Trial Steering Committee (TSC)	77](#_Toc231996681)

[14.4.3	Independent Data and Safety Monitoring Committee (IDSMC)	77](#_Toc231996682)

[15	Publication and Dissemination	78](#_Toc231996683)

[15.1	Publication Policy	78](#_Toc231996684)

[15.1.1	Authorship	78](#_Toc231996685)

[15.2	Dissemination to Key Stakeholders	78](#_Toc231996686)

[15.3	Data Sharing	79](#_Toc231996687)

[16	Chronology of Protocol Amendments	80](#_Toc231996688)

[16.1	Version 1.0 (31/08/2017)	80](#_Toc231996689)

[16.2	Version 2.0 (20/04/2018)	80](#_Toc231996690)

[16.3	Version 3.0 (28/06/2018)	80](#_Toc231996691)

[16.4	Version 4.0 (03/08/2018)	80](#_Toc231996692)

[16.5	Version 5.0 (18/10/2018)	81](#_Toc231996693)

[16.6	Version 6.0 (28/11/2018)	81](#_Toc231996694)

[16.7	Version 7.0 (09/07/2019)	81](#_Toc231996695)

[16.8	Version 8.0 (02/04/2020)	82](#_Toc231996696)

[16.9	Version 9.0 (12/08/2020)	83](#_Toc231996697)

[16.10	Version 10.0 (30/11/2021)	84](#_Toc231996698)

[16.11 Version 11.0 (18/03/2022)	85](#_Toc231996700)

[16.12 Version 12.0 (18/07/2023)	85](#_Toc231996701)

[16.13 Version 13.0 (28/09/2023)	87](#_Toc231996702)

[16.14 Version 14.0 (15/10/2024)	87](#_Toc231996703)

[16.15 Version 15.0 (13/03/2025)	87](#_Toc231996704)

[16.16 Version 16.0 (24/02/2026)	88](#_Toc231996705)

[17	References	89](#_Toc231996707)

[18	Documents Supplementary to the protocol	92](#_Toc231996708)

**Glossary**

| A&E | Accident and Emergency Department |
| --- | --- |
| ABPI | Association of the British Pharmaceutical Industry |
| ACD | Acid Citrate Dextrose |
| AE | Adverse Event |
| ANCOVA | Analysis of covariance |
| AP | Acute pancreatitis |
| Ar | Adverse Reaction |
| AUC | Area under the curve |
| AUDIT | Alcohol Use Disorders Identification Test |
| BRU | Biomedical Research Unit |
| cECT | Contrast enhanced computerised tomography scan |
| ci | Chief investigator |
| CONSORT | Consolidated Standards of Reporting Trials |
| COVID-19 | Corona virus disease 2019 |
| CRF | Case report form |
| CRN | Clinical Research Network |
| CRP | C-reactive protein |
| CTA | Clinical Trial Authorisation |
| CTIMP | Clinical Trial of an Investigational Medicinal Product |
| ctu | Clinical Trials Unit |
| CyTOF | Cytometry by time of flight |
| DNA | Deoxyribonucleic acid |
| DSUR | Developmental Safety Update Report |
| ECG | Electrocardiogram |
| eGFR | Estimated glomerular filtration rate |
| eMC | Electronic Medicines Compendium |
| EQ | EuroQol |
| eQTL | Expression Quantitative Trait Loci |
| EU | European Union |
| EuDract | European Clinical Trials Database |
| GCP | Good Clinical Practice |
| GMP | Good Manufacturing Practice |
| gp | General Practitioner |
| HbA1c | Glycated haemoglobin |
| HRA | Health Research Authority |
| HRUQ | Health Resource Use questionnaire |
| IB | Investigator’s brochure |
| IBD | Inflammatory bowel disease |
| ICH | International Council for Harmonisation of Technical Requirements for Pharmaceuticals for Human Use |
| IDSMC | Independent Data and Safety Monitoring Committee |
| IMP | Investigational Medicinal Product |
| ISRCTN | International Standard Registered Clinical Study Number |
| i.v. | Intravenous |
| LCTC | Liverpool Clinical Trials Centre |
| lhp JRO | Liverpool Health Partners Joint Research Office |
| LUHFT | Liverpool University Hospitals NHS Foundation Trust |
| M | Mean |
| MHRA | Medicines and Health Care Products Regulatory Agency |
| MRI | Magnetic resonance imaging |
| MSD | Merck, Sharp and Dohme Limited |
| N/A | Not applicable |
| NCT | National Clinical Trial |
| N/D | Not determined |
| NHS | National Health Service |
| NICE | National Institute for Heath and Care Excellence |
| NIHR | National Institute for Health and Care Research |
| NIMP | Non-Investigational Medicinal Product |
| NK | Not known |
| NR | Not recorded |
| NRLS | National Reporting and Learning System |
| NYHA | New York Heart Association |
| PBMC | Peripheral blood mononuclear cell |
| PI | Principal investigator |
| PISC | Patient Information Sheet and Consent Form |
| PLICS | Patient-level information and costing systems |
| QA | Quality assurance |
| QALY | Quality-Adjusted Life Years |
| QC | Quality control |
| qPCR | Quantitative polymerase chain reaction |
| RAC | Revised Atlanta Classification |
| R&D | Research and Development |
| RAPID-I | Randomised treatment of Acute Pancreatitis with Infliximab: Double-blind, placebo-controlled, multi-centre phase II trial |
| REC | Research Ethics Committee |
| RN | Research Nurse (Registered) |
| RNA | Ribonucleic acid |
| RSI | Reference Safety Information |
| RSO | Research Support Office |
| SAE | Serious Adverse Event |
| SAP | Statistical Analysis Plan |
| SAR | Serious Adverse Reaction |
| SARS-CoV-2 | Severe acute respiratory syndrome corona virus 2 |
| SDV | Source Data Verification |
| SEM | Standard error of the mean |
| SOFA | Serial Organ Failure Assessment |
| SOP | Standard Operating Procedure |
| SPC | Summary of Product Characteristics |
| SPIRIT | Standard Protocol Item: Recommendations for Interventional Trials 2013 |
| SSAR | Suspected Serious Adverse Reaction |
| susar | Suspected Unexpected Serious Adverse Reaction |
| T | Thymus |
| t.d.s. | Ter die sumendum (three times a day) |
| TMG | Trial Management Group |
| TNFα | Tumour Necrosis Factor alpha |
| Tsc | Trial Steering Committee |
| uar | Unexpected Adverse Reaction |
| UK | United Kingdom |
| UKBC | UK Biocentre |

# 1 Protocol Summary

| Full Title: | Phase IIb, randomised, double-blind, placebo-controlled, multi-centre trial of infliximab with transcriptomic biomarker and mechanism evaluation in patients with acute pancreatitis. | |
| --- | --- | --- |
| Short Title: | Randomised treatment of Acute Pancreatitis with Infliximab: Double- blind, placebo-controlled, multi-centre phase II trial. | |
| Acronym: | RAPID-I | |
| **Phase:** | IIb | |
| **Target Condition:** | Acute pancreatitis (AP) | |
| **Sample size:** | At least 66 participants with evaluable primary outcome data in each arm (provided that no treatment arms are dropped prematurely). | |
| **Trial Design:** | Randomised, double-blind, placebo-controlled, multicentre trial, employing an adaptive trial design with two interim analyses. | |
| **Main Inclusion Criteria:** | Adult patients attending Accident and Emergency (A&E) at or admitted to recruiting hospitals via a GP with a new diagnosis of AP established by two of: (i) typical continuous upper abdominal pain; (ii) amylase and/or lipase three or more times the upper limit of normal; (iii) characteristic findings on abdominal imaging (if undertaken urgently by CT or MRI); *and* in whom trial treatment can be started as soon as possible and within 36 hours of admission, allowing 120 min within the 36 hour period for preparation of trial medication; *and* from whom appropriate consent is obtained (from the patient or their legal representative). | |
| **Main Exclusion Criteria:** | Age <18 or >85; patients with a bodyweight over 200 kilograms (kg); known previous AP diagnosed within the last 30 days or known chronic pancreatitis; known multiple sclerosis, systemic vasculitis, Guillain-Barré syndrome or other demyelinating disorder; known epilepsy; known moderate to severe heart failure and/or coronary disease; known severe respiratory conditions including cystic fibrosis, severe asthma and severe chronic obstructive pulmonary disease (COPD), on home oxygen or home mechanical ventilation; jaundice (serum bilirubin >50 µmol/L) or known advanced liver disease; known cancer for which chemotherapy and/or radiotherapy ongoing/completed in last 6 months before admission; known haematological malignancy; known cancer requiring palliative care; known established infection prior to or suspected infection, including COVID-19, at the time of AP onset; known history of tuberculosis, or household contact with those with tuberculosis or opportunistic infection; known history of infective hepatitis*; known rare diseases or inborn errors of metabolism that significantly increase the risk of infections, including severe combined immunodeficiency (SCID) and homozygous sickle cell disease; known live vaccine, infectious agent or immunosuppressive therapy within one month; known hypersensitivity to infliximab or to inactive components of Infliximab or to any murine proteins; known pregnancy or lactation at admission; women of childbearing potential who do not agree to use adequate contraception up to six months after trial infusion treatment; known participation in investigational medicinal product study within last three months before admission.  *If patient has recovered from a previous Hepatitis A infection, then they do not need to be excluded. | |
| **Study Centres and Distribution:** | Multicentre trial with up to 40 UK recruiting centres. | |
| **Patient Study Duration:** | Inpatient stay for treatment of AP with follow-up of 90 days. | |
| **Description of**  **Agent/Intervention:** | **Intervention:** Blinded single intravenous (i.v.) infusion of 5 mg/kg or 10 mg/kg infliximab in 250 ml (500 ml for patients weighing over 100 kg) 0.9% sodium chloride infused over 2 hours begun as soon as possible and within 36 hours of admission to hospital.*  **Control:** Blinded single placebo i.v. infusion of 250 ml (500 ml for patients weighing over 100 kg) 0.9% sodium chloride.  * Following recommendations from the RAPID‑I Independent Data and Safety Monitoring Committee (IDSMC), endorsed by the Trial Steering Committee (TSC), Arm B (10 mg/kg infliximab) was discontinued on 19^th^ of May 2026. From this date onwards, participants are randomised to Arm A (5 mg/kg infliximab) or Arm C (placebo) only. | |
|  | **Objectives** | **Outcome Measures** |
| **Primary:** | To determine the efficacy of a single i.v. infusion of 5 mg/kg or 10 mg/kg infliximab in the treatment of AP. | C-reactive protein (CRP) on days 2, 4 (+/- 1) and 14 (+/- 2), summated as area under the curve (AUC). |
| **Secondary:** | To evaluate the safety of a single i.v. infusion of 5 mg/kg or 10 mg/kg infliximab in AP.  To develop a clinically meaningful outcome measure for AP that can be used for drug regulatory purposes.  To estimate the cost-effectiveness of a single i.v. infusion of 5 mg/kg or 10 mg/kg infliximab in the treatment of AP.  To develop national capacity in the National Institute for Health and Care Research (NIHR) networks to undertake trials in pancreatitis. | Cumulative pain score, opiate requirements, nutritional deficit (number of days without solid food), decline in serum albumin (negative AUC), rise in neutrophils (AUC), cumulative selective serial organ failure assessment (SOFA) score, local pancreatic injury on contrast-enhanced computerised tomography scan (CECT day 14 +/- 7 days), infection, length of stay, mortality, and patient reported outcome (EQ-5D-5L) on Days 4 (+/- 1 day), 14 (+/- 2 days) and 90 (+/- 7 days). |

## Schematic of Study Design:

Placebo 0.9% sodium chloride infusion effect on CRP

(15 patients*)

5 mg/kg infliximab infusion effect on CRP

(15 patients*)

10 mg/kg infliximab infusion effect on CRP

(15 patients*)

First interim analysis conducted when effect on CRP observed in at least 15 patients in each arm.

Any infliximab arm with mean CRP no lower than placebo to be dropped.

Placebo 0.9% sodium chloride infusion effect on CRP

(40 patients*)

5 mg/kg infliximab infusion effect on CRP

(40 patients*)

10 mg/kg infliximab infusion effect on CRP

(40 patients*)

Placebo 0.9% sodium chloride infusion effect on CRP

(66 patients*)

5 mg/kg infliximab infusion effect on CRP

(66 patients*)

10 mg/kg infliximab infusion effect on CRP

(66 patients*)

Final analysis of all data with total recruitment depending on decisions made at interim analyses

Second interim analysis conducted when effect on CRP observed in at least 40 patients in each remaining arm. Any infliximab arm with mean CRP no lower than placebo to be dropped.

Patients presenting with AP at A&E assessed for eligibility, exclusions recorded

Eligible consenting patients randomised and trial treatment begun within 36 hours of admission

**Figure 1:** **Flow diagram of RAPID-I.**

*Number of patients where primary outcome has been observed, not number of patients recruited.

2 Introduction

2.1 Background

AP is an inflammatory disorder of the pancreas causing excruciating pain, gastrointestinal dysfunction and pronounced systemic inflammatory responses with circulatory and respiratory disturbances that can lead to organ failure and death^1^. AP is one of the commonest gastrointestinal causes of emergency admission to hospital and over the last five decades has increased in incidence in all countries surveyed^1,2^. Despite many randomised clinical trials and systematic reviews there are no accurate means to predict disease course on admission^3^ and there is no licensed specific therapy that ameliorates, let alone aborts, the condition^4,5^. This failure is likely due to the choice of agent and speed of treatment delivery in randomised trials^5,6^, coupled with a lack of focus on widely applicable outcome measures for all patients^5^, not just those developing persistent organ failure (>48 hours). Trial randomisation has been within 72 to 120 hours of admission, very late in the treatment of a medical emergency, complicated by inaccurate prognostic indices to stratify severity. The failure of prognostication, precluding accurate stratification in trials, is in part due to an absence of studies using systems medicine approaches. The work proposed here will address these issues, testing a treatment with robust proof of principle, markedly reduced door-to-needle time and well-defined outcome measures with effective application of systems medicine.

Tumour necrosis factor alpha (TNFα) has a major role in the pathogenesis and severity of acute pancreatitis^7,8^; it is released by injured pancreatic acinar cells in response to toxins^9^ (bile acids, ethanol metabolites) implicated in human AP^6^ and in large amounts by leukocytes^10^, orchestrating cascades in the local and systemic inflammatory responses that are so deleterious in AP^7,11-14^. Circulating TNFα levels rise early and remain elevated for days in human AP, proportional to severity^11-14,^ presenting a suitable drug target to inhibit the amplified immune responses that further damage the pancreas and drive widespread organ dysfunction, but with a potentially large antigen sink from systemic inflammation. Anti-TNF therapy for other indications dramatically reduces the incidence of AP to less than one tenth of that without (OR=0.04, 95% CI=0.01-0.16)^15^, providing the foundation for the proposed trial of the intravenous (i.v.) anti-TNF agent infliximab in AP.

## 2.2 Rationale

TNFα is released in AP by injured pancreatic acinar cells to initiate immune responses in which TNFα is pivotal^9^, driving many deleterious inflammatory cascades^7-15^. The central role of TNFα has been shown in animal AP models, with marked amelioration from anti-TNF treatment^7^. TNFα rises in human AP for a week or more, proportional to disease severity^11-14^, and anti-TNF treatment used for other indications dramatically reduces the incidence of AP to less than one tenth of that without anti-TNF treatment^15^. Anti-TNF therapy has been documented to abrogate human AP^16^. Also, TNFα expression-enhancing -1031C and -863A alleles significantly increase the risk of organ dysfunction in AP^8^. A pilot trial of pentoxyfylline, which has weak anti-TNF effects, has found this treatment associated with reduced admission to and length of intensive care, although no other parameter was affected^17^, suggesting more powerful anti-TNF therapy is required. Infliximab has been selected as it is given via i.v., which will ensure rapid bioavailability to treat AP. This is different from other biologics, which are given subcutaneously.

This trial will determine the efficacy of early initiation of anti-TNF treatment in AP, setting new standards for trials in AP. Using a randomised, double-blind, placebo-controlled adaptive design^18,19^ with two doses of a single i.v. infusion of infliximab at 5 mg/kg or 10 mg/kg, the trial will determine size of effect, safety and potential effectiveness. Proof of concept has been established from extensive preclinical studies, clinical observational and pilot human trials^7-15^. The primary outcome measure of CRP is one of the most widely used measures of inflammatory disease severity^20^ that has robust validity in AP^21^, with representative clinical secondary outcome measures to substantiate efficacy.

High therapeutic response rates have been demonstrated in inflammatory bowel disease (IBD) using 5 mg/kg infliximab, the standard initial dose^22^. Severe IBD, however, presents a greater challenge, with higher TNFα production and faster consumption of infliximab by the reticuloendothelial system, as seen in pharmacokinetic analyses^23,24^. When an initial therapeutic response to 5 mg/kg is lost or in more severe IBD, 10 mg/kg has been shown to be more effective^25-28^, as in rheumatoid arthiritis^29^. Similarly, higher dosing has been found more effective in preventing colectomy in severe IBD^30^. Effectiveness analyses have shown adequate trough levels to be an independent predictor of favourable outcome^25-28^. Although there is substantial experience in the use of infliximab, received by >2 million patients worldwide^31^, dosing varies in different scenarios^22-30^. As systemic inflammation is one hallmark of AP that may result in a very large TNFα antigen sink^22^ and there are few data to inform optimal dosing, the trial will test both doses used in IBD, i.e. a single i.v. infusion of either 5 mg/kg or 10 mg/kg infliximab.

The trial provides a unique platform to undertake studies to validate a transcriptomic prognostic signature that predicts disease severity on admission more accurately than existing means^3^, adding greatly to knowledge by evaluating the efficacy of infliximab in AP on TNFα signalling and downstream effectors. The National Institute for Health Research (NIHR) Liverpool Pancreas Biomedical Research Unit (BRU) identified a differential gene expression signature from blood taken on the day of admission for AP that predicts moderately severe and severe AP (Revised Atlanta Criteria, RAC^32^) with 91% sensitivity, 79% specificity and 87% accuracy. Previous measures applied on admission have at best 74% accuracy (CRP is accurate at 48 hours, but not on admission)^3^. This prognostic signature, identified from bioinformatic analyses of whole transcriptome Affymetrix arrays (see Figure 2) and confirmed by quantitative polymerase chain reaction (qPCR), has now been optimised with a core set of three genes, to target appropriate resuscitation and supportive treatment in more severely affected patients. Three further genes may be added when a different classification (Determinants Based with mild, moderate, severe and critical categories^33^) is used. Functional analyses of the differentially expressed genes have shown TNFα signalling to be pivotal during the first week. There is pronounced suppression of thymus (T) cell function in severe disease, notably suppression of T regulatory cells, consistent with suppressive effects of high levels of TNFα on lymphocytes in AP^11-14^, increasing the risk of infection^11,34,35^. Gene enrichment analysis suggests T cell apoptosis and necroptosis from ligation of tumour necrosis factor receptor 1; these major transcriptional changes will be used in the exploratory assessment of efficacy and safety of infliximab treatment. If response to infliximab is most evident in severe AP, the prognostic signature may also be clinically useful in stratification for different doses; alternatively, response to infliximab may be predicted and/or monitored by a new transcriptomic signature.

**Figure 2. Heat map of differentially expressed genes** (red +4 to green –4 on Affymetrix whole transcriptome microarrays) in blood from 92 patients with mild (M) versus severe (S, moderate and severe, RAC) AP on day one (D1, 1410 differentially expressed genes), two (1823), seven (395) and 14 (10 differentially expressed genes, but fewer samples available from later days). These data were used to derive the prognostic signature to be validated and define the 100 transcipts for exploratory efficacy and safety analyses of infliximab in AP, examining TNFα signalling, downstream effects, T cell function, and immune competence.

Comprehensive data from the BRU AP cohort mirroring the anticipated trial recruitment confirm the validity of serum CRP levels (measured as area under the curve, AUC) as the primary outcome measure (see Figure 3).

**Figure 3**. **Changes in serum (A) CRP and (B) albumin in 333 AP patients** with mild, moderate, severe or fatal (12 patients) AP (RAC) over four weeks from admission, showing the number of patients measured at each time point. Strong correlation is shown between these two parameters and the clinical impact of AP.

The data shown from a large patient cohort matching the inclusion and exclusion criteria of the trial confirm CRP rose in all patients to a level and for a period proportional to severity (normal <10 mg/L): in mild AP to 80 +/- 10 mg/L (mean +/- standard error of the mean, M +/-SEM), elevated for two weeks; moderate to 220 +/- 30 mg/L, elevated for four weeks; severe to 320 +/- 40 mg/L, elevated longer; in fatal AP as in severe AP, but remaining highly elevated.

CRP is widely used as a robust tool to determine clinical management in AP^1,3,21^ and other inflammatory diseases^20^, including composite scores in regulatory drug approval^36-38^; CRP falls in response to supportive intervention in AP^39^. Changes in CRP over time can be summarised by the AUC, which the BRU cohort shows to be directly proportional to disease outcome (r=0.637, p<0.001), notably the presence and duration of organ failure. AUC captures the whole clinical course far more comprehensively and accurately than a single measure, whether a maximum value or on a specific day. As shown in Figure 3, measurement of CRP on days 2, 4 and 14 offers the most efficient means of capturing AUC, since between days 4 and 14 the fall in CRP is linear, and after day 14 the difference in AUC between mild, moderate and severe AP is less; more frequent patient sampling presents practical difficulties in patient recall, notably with the reduction of outpatient face-to-face contact introduced during the corona virus disease 2019 (COVID-19) pandemic. The validity of the secondary outcome measures has similarly been confirmed in this cohort, exemplified here by serum albumin levels (see Figure 2).

2.2.1 Benefits and Risks

AP accounts for over 50% of all admissions to hospital for pancreatic digestive disease, with an incidence of 30-50/100,000^1,2^. In ~70% of patients AP results in hospital stays of 6-7 days with pain and nutritional deficit requiring opiates and nutritional support, plus investigation and appropriate management of the commonest causes, these being gallstones (cholecystectomy +/- endoscopic sphincterotomy) or alcohol excess (counselling/abstinence/support), as well as hyperlipidaemia, hypercalcaemia or rarer causes^1,40^. After discharge patients typically require 3-6 weeks off work, plus further time dependent on cause. The outcome of such cases is considered mild in the 2013 RAC^32^, although severity is a continuum, and the total number of bed days occupied is greatest for patients with this severity level of AP. Ideally a treatment would abort/cure AP, preventing admission and permitting early return to work. Realistically the first effective treatment is likely to ameliorate not abort AP, yet will be a substantial improvement in reducing unmet need.

In ~20% of patients AP results in significant organ dysfunction or organ failure (<48 hours) and/or local pancreatic injury with acute fluid collections and/or necrosis that cause more prolonged pain, nutritional deficit and hospital stays over 2 weeks^1,32,40^. After discharge patients will typically require 6-12 weeks or more off work, dependent on cause and requisite management. Such cases are moderate in RAC^32^.

In ~10% of patients AP results in persistent organ failure (>48 hours), likely with local injury causing more prolonged pain, prolonged nutritional deficit and hospital stays over 4 weeks^1,32,40^. Critical and/or high dependency care is required, often with intervention for pancreatic necrosis. Death is likely in up to half of this group, resulting in an overall likelihood of death in all cases of AP of 1-5% (mild, moderate and severe)^1,2^. After discharge surviving patients will typically have to take 12 or more weeks off, or may never return to work. Such cases are severe in RAC^32^.

There is thus an urgent need for an effective treatment for AP. If the proposed trial demonstrates efficacy of infliximab in AP, a route will be established to a major improvement in the health of a major group of National Health Service (NHS) patients, reducing the human suffering from AP and permitting earlier return to normal life, reduced hospital stays and associated costs. In time there would be potential expansion of a range of therapeutic options. If the proposed trial does not demonstrate efficacy, there will nevertheless be development of national infrastructure to undertake trials in AP, refinement of measures and quality indicators for trials in AP, potential validation of a new prognostic in AP, and deeper understanding of the immune disorders in AP.

There is a risk to individual trial participants of infliximab-related i.v. infusion reactions, categorized as mild, moderate, severe, life-threatening or fatal^41,42^. These have been reported in 5-25% of patients on long-term infliximab, the majority of which occur after repeated administration and are mild, e.g. pruritus, flushing and myalgia, not requiring modification of the infusion^42^. Hydrocortisone and chlorphenamine will be given to all patients, with adrenaline, further chlorphenamine and paracetamol prescribed as required, prior to graded infusion with cautious initial dosing (protocol of standard care for other indications). Moderate reactions, e.g. chest tightness, urticaria, hypertension, would prompt infusion interruption, chlorphenamine and/or paracetamol and with favourable response, resumption of infusion^42^. Severe or worse reactions (<1% of patients) warrant immediate cessation of the infusion and emergency management with adrenaline, resuscitation and intensive support^42^. Delayed serum sickness-like reactions occur predominantly with repeated administration of infliximab and are usually self-limiting^42^.

The other significant risk is an increased likelihood of infection, notably exacerbation or reactivation of tuberculosis, as well as potential reactivation of chronic viral infection and exacerbation of any bacterial infection consequent upon AP^43,44^. The risks of tuberculosis are predominantly associated with long-term administration of infliximab, as for inflammatory bowel disease or autoimmune arthritides (0.2-0.3 per 100 patient years^44^). Nevertheless, exclusion criteria will include a history of tuberculosis, including that identified on chest x-ray, or chronic viral infection^44^. Tuberculin testing will take too long, and interferon gamma release assays will be compromised by potential suppression of the adaptive immune response by AP. Since trial treatment is to be initiated within 36 hours of admission to hospital (for a disease with an overall risk of organ failure of 20-30% and mortality of 1-5%), it is not feasible to undertake prior screening tests for chronic viral infection. The most significant viral risk is reactivation of hepatitis B, addressed by combined hepatitis B surface antigen and core antibody testing after trial entry and local monitoring of liver function tests (by participating sites) during follow-up, with treatment in the unlikely event of reactivation^43^.

Evidence suggests that patients on long-term anti-TNF biologics are not at increased risk of severe COVID-19^45,46^, and as a result of calls for anti-TNF trials in patients with COVID-19^47^, the CATALYST trial assessed infliximab in the treatment of COVID-19 pneumonia^48^. All patients who received infliximab in the CATALYST trial also received dexamethasone as standard therapy, which inhibits TNF and its downstream signalling; while no benefit was found for the addition of infliximab to dexamethasone, this addition was found to be safe. A range of immunotherapies have an established place in the treatment of COVID-19^49^, with improved outcomes from reduction of the storm that contributes to severe COVID-19 disease and mortality. There is emerging evidence that anti-TNF biologics have conferred some protection from COVID-19 on patients with inflammatory disease^50^ and on patients with rheumatoid arthritis^51^. Nevertheless, the entry criteria in RAPID-I ensure that all patients identified by NHS Digital to be at high risk from infection with severe acute respiratory syndrome corona virus 2 (SARS-CoV-2) are excluded from the trial. Throughout all procedures conducted in the trial, all relevant precautions will be taken to prevent the spread of SARS-CoV-2 and COVID-19 infection, according to all national and local guidelines in force, including required use of personal preventive equipment (PPE), social distancing and local policies on testing. Nationally mandated vaccination advice for patients admitted to hospital with a moderate or severe acute illness such as acute pancreatitis is for vaccination to be delayed until the acute illness has improved^52,53^, typically four weeks after discharge, in an endeavour to ensure adequate vaccination responses. By that time, infliximab administered as trial medication will be cleared (elimination half-life 7-12 days, mean residence time 12-17 days^54^) by the reticulo-endothelial system and will not compromise vaccination responses. As standard of care for acute pancreatitis, patients who have not received a first, second or subsequent SARS-CoV-2 vaccination and are recruited into RAPID-I are advised to delay SARS-CoV-2 vaccination for four weeks after discharge, subject to personalised advice from their local clinical teams (e.g. if there is an ongoing problem such as intra-abdominal sepsis undergoing drainage, this could be longer), to best ensure protective immunological responses^52,53^.

Although	routine	prophylactic	antibiotics	against	bacterial infection are not recommended in AP^40^, the BRU found bacteraemia very early in some patients with AP and all patients in this trial will be prescribed piperacillin/tazobactam 4.5 g t.d.s for up to 7 days or until discharge, whichever is earlier (or the local centre’s antibiotic(s) for intra-abdominal infection, e.g. in the case of penicillin allergy), starting at trial entry. To further ensure the safety of trial participants, there may be a co-investigator at each NHS centre with extensive experience of anti-TNF treatment for IBD. The adaptive trial design with two interim analyses has safety as a critical consideration for the Independent Data and Safety Monitoring Committee (IDSMC) in determining whether to advise continuation.

## 2.3 Objectives

### 2.3.1 Primary Objective

The primary objective is to determine the efficacy of a single i.v. infusion of 5 mg/kg and 10 mg/kg infliximab in the treatment of AP.

### 2.3.2 Secondary Objectives

1. To evaluate the safety of a single i.v. infusion of 5 mg/kg or 10 mg/kg infliximab in AP.
2. To evaluate differential gene expression of 100 genes to:
i. Validate an expression signature for accurate prediction of severe AP;
ii. Undertake exploratory efficacy and safety analyses with representative transcriptomes of TNFα signaling and immune competence, including expression quantitative trait loci (eQTL);
iii. To develop a gene expression signature to predict treatment response;
iv. To increase knowledge of how infliximab is working in this patient group and develop stratified approaches, through mechanistic evaluation, complemented by cytokine and leukocyte subset analysis.
3. To validate a cumulative AP outcome score derived from the primary and secondary outcome measures of efficacy, in line with verbal and written advice obtained from the Medicines and Healthcare products Regulatory Agency (MHRA), European Medicines Agency and Food and Drug Administration to develop a clinically meaningful outcome measure that can be used for drug regulatory purposes^36-38^.
4. To estimate the cost-effectiveness of infliximab expressed as the incremental cost per quality-adjusted life-year (QALY) gained, adopting a health and personal social service perspective.
5. To develop national capacity in the NIHR networks to undertake trials in pancreatitis.

## 2.4 Outcome Measures/Endpoints

All measures have been widely used previously and confirmed in the BRU cohort to display abnormality that is proportional to severity, correlating strongly with major clinical outcomes.

### 2.4.1 Primary Outcome/Endpoint

The primary efficacy outcome measure will be the difference in mean serum CRP measured on days 2, 4, and 14 (summated as AUC) in the active arms (5 mg/kg or 10 mg/kg) versus the placebo arm. CRP assays will be undertaken centrally to ensure standardised measurement.

### 2.4.2 Secondary Outcomes/Endpoints

Efficacy:

1. Cumulative pain scores (numerical rating scale from 0 to 10 daily for first 14 days*).
2. Opiate requirements (morphine equivalents daily for first 14 days*).
3. Nutritional deficit (number of days without solid food for first 14 days*).
4. Decline in serum albumin (negative AUC from admission value for first 14 days*).
5. Rise in circulating neutrophils (AUC for first 14 days*).
6. Cumulative selective serial organ failure assessment^55^ (SOFA for first 14 days*) score.
7. Local pancreatic injury on CECT scan (Day 14 +/- 7 days).
8. Infective complications (within first 90 days).
9. Length of hospital stay (up to 90 days).
10. Mortality (within first 90 days).
11. Patient reported outcome (EuroQol EQ-5D-5L^56^ including the visual analogue scale EQ-VAS) at Day 4 (+/- 1 day), Day 14 (+/- 2 days) and Day 90 (+/- 7 days).

* Assessments made during inpatient stay only.

Safety:

12. Potential safety signals^57^ will be adverse events related to infliximab including infusion reactions and delayed serum sickness reactions (within first 90 days).
13. A further safety signal will be a significant increase in the incidence of infective complications associated with the use of infliximab over placebo (see item 8 above).
14. Assay for antibodies to infliximab will be undertaken at Day 14 (+/- 2 days).

Transcriptomics:

15. Absolute and/or relative expression of selected transcripts from samples taken on Days 2, 4 (+/- 1 day) and 14 (+/- 2 days).
16. Cytokine and leucocyte subsets profiles on Days 2, 4 (+/- 1 day) and 14 (+/- 2 days).

Outcome measures for AP trials:

17. Discriminant function (trial treatment versus placebo) of efficacy measures across domains: clinical, laboratory, critical care, local injury, infection, length of stay and patient reported outcome domains.

Health economics:

18. NHS costs, based on resource utilisation at Days 14 (+/- 2 days) and 90 (+/- 7 days) assessed using a Health Resource Use Questionnaire (HRUQ) and routinely collected data from Patient-Level Information and Costing Systems (PLICS)
19. Incremental cost per QALY gained by trial treatment.

National AP trial capacity:

20. Time to recruitment of target sample size, this number depending on the adaptive design.

3 TRIAL DESIGN

RAPID-I is a randomised, placebo-controlled, double-blind, multi-centre, three-arm, phase IIb efficacy trial of infliximab in patients with AP. Patients will be randomised (1:1:1 allocation ratio) to receive an i.v. infusion of either 5 mg/kg or 10 mg/kg infliximab or placebo, initiated within 36 hours of admission to hospital. A delegated member of the research team will randomise online. Treatment allocation will only be revealed to Pharmacy or unblinded staff preparing trial treatment in a separate biologics preparation area (e.g. Clinical Research Facility or Day Case Biologics Treatment Facility or Ward) to ensure the research team administering the treatment remain blinded. The feasibility of this design is predicated on experience from the NIHR Pancreas BRU rate of recruitment of patients with AP on the day of admission to observational studies, as well as responses to the feasibility questionnaires sent to six sites. Standard Protocol Items: Recommendations for Interventional Trials (SPIRIT)^58^, Consolidated Standards of Reporting Trials (CONSORT)^59^ and Consolidated Health Economic Evaluation Reporting Standards (CHEERS)^60^ guidelines will be followed throughout.

Up to three analyses of the efficacy data (two interim analyses and a final analysis) are planned, as a smaller expected sample size can be achieved by using interim analyses. The first will take place after the primary outcome has been observed for 15 patients in each arm. At this point any treatment arm with a mean CRP AUC value larger or equal to the placebo group will be dropped. Should both treatment arms be dropped, the study will stop. A second interim analysis will take place after the primary outcome has been observed for at least 40 patients in each of the remaining arms. The stopping rules will be as for the first interim analysis.

At the final analysis comparative t-statistics will be computed using an analysis of covariance (ANCOVA) model. If the resulting test statistic for either dose falls below a critical value of -2.196, the corresponding null hypothesis of no difference between the active group and placebo can be rejected and superiority of the active dose can be claimed. Note that it is possible to claim superiority of both doses over placebo.

The revised design has an overall (family-wise) error rate of 2.5% and 80% power (see 10.3 for details of the sample size calculations) and has been specified such that any treatment worse than placebo at interim analysis will be dropped. To account for (potentially) dropping a dose for other reasons, the conditional error principle will be used to adjust the design^19^. This will ensure that the overall type I error rate of the study is still controlled and result in an increased power compared to not accounting for this unplanned modification.

4 Study Setting and Selection of Centres/Clinicians

## 4.1 Selection of Centres/Clinicians

There are two different types of participating centre contributing to the RAPID-I trial:

1. Patient recruitment and data collection centres
2. Data collection only centres

4.1.1 Criteria for Patient Recruitment and Data Collection Centres

1. An NHS Hospital Trust providing adult A&E, Gastroenterology and General Surgical services.
2. Gastroenterology services with expertise in biologic therapies.
3. A Principal Investigator who may be an emergency medicine physician, or gastroenterologist with expertise in biologics, or surgeon or physician responsible for the management of patients with acute pancreatitis, the others having the option to be Co-Investigators at the centre, if desired.
4. Sufficient research capacity comprised of staff, time and facilities to undertake the trial, including patient screening and recruitment (recruitment is anticipated at most centres during office hours, but is not restricted to these hours), randomisation, collection, centrifugation (clotted blood) and forwarding to the UK Biocentre of blood samples in pre-paid collection kits provided, preparation and blinded administration of trial treatment by infusion pump initiated within 36 hours of admission in patients with acute pancreatitis, daily inpatient and scheduled outpatient assessments including CECT on Day 14 +/- 7 days, collection and provision to the LCTC of all required data, identification and management of adverse events including notification to LCTC within protocol defined timeframes, identification and provision of information to the LCTC of all protocol breaches.
5. Pharmacy Department, or a separate biologics preparation area (e.g. Clinical Research Facility or Day Case Biologics Treatment Facility or Ward), with capacity and capability for aseptic preparation of trial medication within 120 min of request immediately following consenting of patient or within a period that will allow administration of trial medication to the patient within 36 hours of admission, and which will maintain the necessary double-blinding of the trial team and patient.
6. Pharmacy Department, or suitable alternative, with capacity and capability for temperature-controlled storage of trial medicine vials.
7. Capacity to transfer blood samples (using pre-paid collection kits provided) to the UK Biocentre within protocol defined timeframes.
8. All research staff contributing to the trial must have valid certified GCP training throughout the conduct of the trial. Requirements of the Sponsor are that renewal of GCP must occur every 3 years.
9. The Centre works to the International Association of Pancreatology/American Pancreatic Association^40^ and NICE evidence-based guidelines^61^ for the management of AP.
10. All secondary care centres must have capacity and capability to transfer patients to a participating tertiary care site with whom the secondary care centre is networked.

4.1.2 Criteria for Data Collection Only Centres

1. An NHS Hospital providing Gastroenterology and General Surgical services.
2. Gastroenterology services with expertise in biologic therapies.
3. A Principal Investigator who may be a gastroenterologist with expertise in biologics or surgeon or physician responsible for the management of patients with acute pancreatitis, the other having the option to be Co-Investigator at the centre, if desired.
4. Sufficient research capacity comprised of staff, time and facilities to undertake follow-up trial visits, including blood sample collection, centrifugation (clotted blood) and forwarding to the UK Biocentre of blood samples, daily inpatient and scheduled outpatient assessments including CECT on Day 14 +/- 7 days, collection and provision to the LCTC of all required data, identification and management of adverse events including notification to LCTC within protocol defined timeframes, identification and provision of information to the LCTC of all protocol breaches. It is anticipated, however, that of the minority of patients recruited into RAPID-I who are transferred to Data Collection Only Centres, most will be transferred after the required trial blood sampling times.
5. Capacity to transfer blood samples (using pre-paid collection kits provided) to the UK Biocentre within protocol defined timeframes.
6. All research staff contributing to the trial must have valid certified GCP training throughout the conduct of the trial. Requirements of the Sponsor are that renewal of GCP must occur every 3 years.
7. The Centre works to the International Association of Pancreatology/American Pancreatic Association^40^ and NICE evidence-based guidelines^61^ for the management of AP.

**N.B. Centres responsible for data collection only will not be required to undertake randomisation, preparation and administration of trial treatment or IMP management; they will be completed by the recruitment site, prior to patient transfer (should this be needed).**

It is anticipated that Data Collection Only Centres will usually be tertiary care centres receiving trial patients transferred for provision of specialist pancreatic services e.g. for the management of pancreatic necrosis. Occasionally Data Collection Only Centres may be secondary care centres receiving trial patients transferred for other reasons e.g. to be nearer their homes. Centres fulfilling the criteria above will be selected to be Recruitment and Data Collection Centres or Data Collection Only Centres. Following obtention by the LCTC of all necessary global approvals (Health Research Authority, HRA; Research Ethics Committee, REC; MHRA), centres will be opened to recruitment and/or data collection once local approval has been issued (“Capacity and Capability” confirmation/Research and Development (R&D) Approval), and once study-specific conditions (e.g. site personnel training requirements) and all necessary documents have been returned to LCTC as detailed in the trial ‘greenlight’ checklist.

# 5 Study Population

## 5.1 Inclusion Criteria

1) Adult patients attending A&E at or admitted to recruiting hospitals via a General Practitioner with a new diagnosis* of AP established by two of:

i.	typical continuous upper abdominal pain;

ii.	amylase and/or lipase three or more times the upper limit of normal;

iii.	characteristic findings on abdominal imaging (if undertaken urgently by CT or magnetic resonance imaging, MRI);

*and*

2) Patients in whom trial treatment can be started as soon as possible and within 36 hours† of admission, allowing 120 min within the 36-hour period for preparation of trial medication;

*and*

3) Patients from whom appropriate consent is obtained (consent to be given by the patient or their legal representative).

* Not readmission for continuing AP from a previous diagnosis of AP

† Every effort should be made to start the trial infusion as soon as possible after admission. The 36-hour window allows inclusion of patients identified too late on their day of admission to have trial medication prepared in time for trial infusion to be started on the day of admission.

**N.B. Please note all severity levels of AP are to be included in the trial.**

## 5.2 Exclusion Criteria

1) Age <18 or >85.
2) Patients with a bodyweight of over 200 kilograms.
3) Known previous AP diagnosed within the last 30 days* or known chronic pancreatitis.
4) Known multiple sclerosis, systemic vasculitis, Guillain-Barré syndrome or other demyelinating disorder.
5) Known epilepsy.
6) Moderate to severe heart failure and/or coronary heart disease (New York Heart Association† (NYHA) Functional Class III/IV).
7) Known severe respiratory conditions including cystic fibrosis, severe asthma and severe chronic obstructive pulmonary disease (COPD), on home oxygen or home mechanical ventilation‡.
8) Jaundice (serum bilirubin >50 µmol/L), and/or known advanced liver disease, on waiting list for liver transplantation or considered unsuitable for transplantation.
9) Known cancer for which chemotherapy and/or radiotherapy is ongoing or was completed within less than 6 months from admission.
10) Known haematological malignancy.
11) Known cancer that is end-stage with ongoing palliative care or for which palliative care is appropriate.
12) Known established infection prior to or suspected infection, including COVID-19§, at the time of AP onset.
13) Known history of (including that identified on chest x-ray) or household contact with individuals who have tuberculosis or opportunistic infection.
14) Known history of infective hepatitis.¶
15) Known rare diseases or inborn errors of metabolism that significantly increase the risk of infections, including severe combined immunodeficiency (SCID) and homozygous sickle cell disease**.
16) Known immunosuppressive or biologic therapy within one month of admission.
17) Known live vaccines or therapeutic infectious agents within one month of admission.
18) Known hypersensitivity to infliximab or to inactive components of infliximab to any murine proteins.
19) Known pregnancy or lactation at the time of admission.
20) Women of childbearing potential who do not agree to use adequate contraception, (includes prior surgical sterilisation; birth control pill, patch or injection; intrauterine device; male or female condom (but not both); female cap with spermicide or total sexual abstinence††; but not periodic abstinence, withdrawal or spermicides only), up to 6 months after trial infusion treatment.
21) Known to be currently participating in a trial testing any investigational medicinal product or participation in a clinical study involving a medicinal product in the last three months.

***** Patients with a known previous diagnosis of AP made outside this 30-day period can be included provided they have a new diagnosis of a new attack of AP and are not being readmitted for continuing AP from a previous diagnosis of AP.

† **Table 2. New York Heart Association (NYHA) Classes**

| **Class** | **Patient Symptoms** |
| --- | --- |
| I | No limitation of physical activity. Ordinary physical activity does not cause undue fatigue, palpitation, dyspnoea or angina. |
| II | Slight limitation of physical activity. Comfortable at rest. Ordinary physical activity results in fatigue, palpitation, dyspnoea or angina. |
| III | Marked limitation of physical activity. Comfortable at rest. Less than ordinary activity causes fatigue, palpitation, dyspnoea or angina. |
| IV | Unable to carry out any physical activity without discomfort. Symptoms of heart failure or angina at rest. If any physical activity is undertaken, discomfort increases. |

‡ Severe asthma requires high dose inhaled steroid plus a second controller and/or systemic steroids for control or may be uncontrolled despite this therapy; severe COPD is defined by FEV_1_ <50% but will be unreliable at presentation of AP so must be identified by medical history before the onset of AP including periods of extreme difficulty breathing and talking, with chronic productive cough.

§ Symptoms distinct from AP include new continuous cough, loss of taste or sense of smell; national and local policies on testing to be followed but as this is an emergency medicine trial SARS-CoV-2 testing is not included in an entry criterion.

¶ If patient has recovered from a previous hepatitis A infection, then they do not need to be excluded.

** Rare diseases affect fewer than 1 in 2,000 people and 80% are rare genetic disorders.

†† Total sexual abstinence is defined as per the Clinical Trials Facilitation Group Guidance: Sexual abstinence is considered a highly effective method only if defined as refraining from heterosexual intercourse during the entire period of risk associated with the study treatments. The reliability of sexual abstinence needs to be evaluated in relation to the duration of the clinical trial and the preferred and usual lifestyle of the subject.

## 5.3 Co-enrolment Guidelines

Individuals who have participated in a trial testing any investigational medicinal product in the three months preceding screening will be ineligible for the RAPID-I trial. Where recruitment into another trial that does not involve an investigational medicinal product or other trial intervention (e.g. observational study) is considered to be appropriate and without having any detrimental effect on the RAPID-I trial, this must first be discussed with the LCTC who will contact the Chief Investigator (CI, Professor Robert Sutton).

# 6 Recruitment and Randomisation

## 6.1 Participant Identification and Screening

A screening log will be maintained of all the patients who undergo screening, regardless of whether they decide to participate in or are deemed eligible for the trial, to provide important information for monitoring purposes. Reasons for not being eligible and reasons for declining to participate will be asked routinely but it will be made clear that patients or their legal representatives do not have to provide a reason unless happy to do so. Copies of the completed screening log should be sent to LCTC at the end of each month.

Patients will be reviewed upon their arrival in the A&E Department or receiving Ward. As part of standard care, full medical history and concomitant medications will be obtained, a physical examination will be completed, blood and urine tests will be performed (full blood count, urea, creatinine, electrolytes, albumin, calcium, glucose, amylase and/or lipase, pulse oximetry and/or arterial blood gases and urine pregnancy test in women of childbearing age; one or two tests may be undertaken after the diagnosis of AP is made e.g. triglycerides, arterial blood gas), an electrocardiogram (ECG) and chest x-ray will be carried out. Those patients who meet the criteria for the diagnosis of AP and who are suitable for inclusion (see 5.1 and 5.2) will be provided with information about the trial (both verbal and written) to consider participation. At this point, Pharmacy, or the unblinded research team responsible for preparation of trial medication in the separate biologics preparation area, will be informed about potential recruitment of a patient into RAPID-I.

Only a doctor authorised on the site Delegation Log can confirm full eligibility of any patient; a record of this confirmation must be made in the patient’s medical notes on the date of screening.

**N.B. Recruitment is anticipated during office hours but is not restricted to these hours if additional capacity is available.**

**The allocated time windows for each scheduled trial time point (subsequent to baseline and randomisation) allow almost all time points to be completed within office working hours (Mon-Fri, 09:00 – 17:00). However, for patients who are recruited on a Friday, their Day 2 time point (12-36 hours after the start of trial infusion) will have to be completed on a Saturday. If a research site does not have capacity to complete the Day 2 time point during the weekend and are unable to collect and process the central trial blood samples, then patient recruitment should be restricted to Monday to Thursday.**

## 6.2 Informed Consent

Upon diagnosis of AP and following screening, suitable patients will be provided with verbal and written information about the trial and asked to provide written informed consent. As AP is an acute, life threatening condition that may inhibit higher brain function and the trial is set in an emergency setting, it is possible that a number of patients will be lacking capacity. In this case, if a Personal Legal Representative (as defined by the Medicines for Human Use (Clinical Trials) Regulations 2004 and amendments) is available, they must be asked for a proxy decision, and if not available, a Professional Legal Representative (as defined by the Medicines for Human Use (Clinical Trials) Regulations 2004 and amendments) will be asked for a proxy decision on the patient’s behalf (see Section 6.2.2).

The following consent forms will be signed as appropriate depending on the capacity of the patient:

- RAPID-I Patient Information Sheet and Consent form (Patient with Capacity)
- RAPID-I Personal Legal Representative Information Sheet and Consent form (Patients Lacking Capacity)
- RAPID-I Professional Legal Representative Information Sheet and Consent form (Patients Lacking Capacity)
- RAPID-I Patient that Regains Capacity Information Sheet and Consent Form (Patient Regaining Capacity)

### 6.2.1 Obtaining Informed Consent

Informed consent is a process initiated prior to an individual agreeing to participate in a trial and continues throughout the individual’s participation. Informed consent is required for all patients participating in LCTC coordinated trials. In obtaining and documenting informed consent, site staff should comply with applicable regulatory requirements and should adhere to GCP and to the ethical principles that have their origin in the Declaration of Helsinki.

Site staff delegated by the Principal Investigator (PI) and appropriately trained with experience in obtaining informed consent will conduct an interview with the patient (or their Legal Representative in the case of patients lacking capacity). The appropriate ethically approved Patient Information Sheet and Consent form (PISC) will also be provided, describing in detail the trial interventions/products, trial procedures and risks. The patient (or his/her Legal Representative) will be asked to read and review this document. Upon reviewing the document, the site staff will explain the research study to the patient (or his/her Legal Representative), including a discussion of the objectives, risks and inconveniences of the trial and the conditions under which it is to be conducted. This information will emphasise that participation in the trial is voluntary and that the participant may withdraw from the trial at any time and for any reason. A contact point where further information about the trial may be obtained will be provided.

All patients (or their Legal Representatives) will be given the opportunity to ask any questions that may arise, the opportunity to discuss the study with their relatives, carers and/or associates and time to consider the information prior to agreeing to participate. As the approach for consent will take place in emergency settings, a fixed period cannot be prescribed over which the decision to participate should be considered. The amount of time to consider participation in RAPID-I before consent will differ on a case-by-case basis but in every case, it should allow for sufficient time to confirm full eligibility, to carry out randomisation and to commence treatment within 36 hours of admission.

Consenting patients (or their Legal Representative in the case of patients lacking capacity) will then sign and date the informed consent document. Both the person obtaining consent and the patient (or their legal Representative) must personally sign and date the form. The original signed copy of the consent form must be filed in the Investigator Site File, and three copies must be made. One copy will be given to the participant or their Legal Representative for their records, another copy will be kept in the participant’s medical notes (if the site has electronic notes the consent form will be scanned) and a copy should be sent to the LCTC. Consent forms should be submitted to the LCTC ideally within 24 hours, but no later than 7 days of consent being obtained. The process of informed consent will also be detailed in the patient’s medical notes.

Consent from the patient (or his/her Legal Representative in case of a patient lacking capacity) must be obtained prior to any trial-specific procedures or assessments and prior to randomisation, after a full explanation has been given of the treatment options, including the conventional and generally accepted methods of treatment.

A patient may, without being subject to any resulting detriment, withdraw from the trial at any time by revoking their informed consent (similarly a Legal Representative may withdraw the patient under the same conditions). It will be emphasized to all patients (and their Legal Representatives) that their welfare rights (or those of the patient) will be protected and the quality of their medical care will not be adversely affected if they decline to participate in this study or decide to withdraw consent at a later time. The right of the patient (or his/her Legal Representative) to refuse to consent, or to withdraw without giving any reason, must be respected.

After the patient has entered the trial, the clinician is free to give alternative treatment to that specified in the protocol, at any stage, if s/he feels it to be in the best interest of the patient. Nevertheless, the reason for doing so should be recorded and the patient will remain within the trial for the purpose of follow-up and data analysis according to the treatment option to which they have been allocated.

### 6.2.2 Patients Lacking Capacity

As AP is an acute, life threatening condition that may inhibit higher brain function and the trial is set in an emergency setting, it is possible that a number of patients will be lacking capacity. Considering that the benefits of inclusion of patients lacking capacity outweigh the risks (there are no data to indicate that more adverse events (AEs) due to the study interventions would arise in such patients), that the odds for randomisation to the active treatment are 2:1 and that these patients could potentially benefit the most from receiving the trial intervention due to the severity of their condition, such patients will be included if eligible.

All patients must be assumed to have capacity unless it is established that they do not. The site research team will assess patient capacity in accordance with their local practices.

**Table 1 – Hierarchy of informed consent for an incapacitated adult**

| ***England, Wales and Northern Ireland*** | ***Scotland*** |
| --- | --- |
| *1. Personal Legal Representative*  A person not connected with the conduct of the trial who is:   a) suitable to act as the legal representative by virtue of their relationship with the adult, *and* b) available and willing to do so. | *1. Personal Legal Representative*  1A. Any guardian or welfare attorney who has power to consent to the adult’s participation in research as defined in the Adults with Incapacity (Scotland) Act 2000.  1B. If there is no such person, the adult’s nearest relative as defined in section 87(1) of the Adults with Incapacity (Scotland) Act 2000. |
| *2. Professional Legal Representative*  A person not connected with the conduct of the trial who is:   a) the doctor primarily responsible for the adult’s medical treatment, *or* b) a person nominated by the relevant health care provider (e.g. an acute NHS Trust or Health Board).   A Professional Legal Representative may only be approached if no suitable Personal Legal Representative is available. | *2. Professional Legal Representative*  A person not connected with the conduct of the trial who is:   a) the doctor primarily responsible for the adult’s medical treatment, *or* b) a person nominated by the relevant health care provider.   A Professional Legal Representative may only be approached if it is not reasonably practicable to contact either 1A or 1B before the decision to enter the adult into the trial is made. |

Please see the table above for the hierarchy of informed consent from Legal Representatives if a patient is assessed to be lacking capacity.

In those with impaired capacity if a Personal Legal Representative is available, they will be approached to consider participation in the trial and will be asked for a proxy decision on behalf of the patient. If not available, a Professional Legal Representative will be approached and asked for a proxy decision. Every effort will be made to identify a suitable Personal Legal Representative, prior to approaching a Professional Legal Representative.

If the patient regains capacity (which will be the case for most patients), they will be informed about the trial and invited to continue with trial participation. They will be asked to sign the Patient that Regains Capacity Information Sheet and Consent form, alternatively they may choose to withdraw their involvement (patient follow-up).

6.2.3 Patients and/or Legal Representatives Unable to Read

If a patient is unable to read or if a legally acceptable representative is unable to read (should the patient be determined to lack capacity), an impartial witness should be present during the **entire** informed consent discussion. After (i) the written informed consent form and patient information sheet is read and explained to the patient or the patient’s legally acceptable representative, and (ii) after the patient or the patient’s legally acceptable representative has orally consented to the patient’s participation in the trial and (iii) if capable of doing so, has signed and personally dated the informed consent form, the witness should sign and personally date the consent form. The details of the independent/impartial witness should also be recorded in the patient’s medical notes.

By signing the consent form, the witness attests that the information in the consent form and patient information sheet was accurately explained to, and apparently understood by, the patient or the patient’s legally acceptable representative, and that informed consent was freely given by the patient or the patient’s legally acceptable representative.

**N.B. An independent/impartial witness should personally sign and date the consent form in addition to the patient or their legal representative, if the patient/legal representative is unable to read. A witness does not consent on behalf of the patient or their legal representative.**

An independent/impartial witness must be a person independent of the trial, who cannot be unfairly influenced by people involved in the trial, who attends the informed consent process if the patient or the patient’s legally acceptable representative cannot read, and who reads the consent form and patient information sheet supplied to the patient.

6.2.4 Ongoing Informed Consent

The local research team should reaffirm the patient’s willingness (or Legal Representative’s willingness for the patient, whilst the patient lacks capacity) to continue in the trial throughout their participation.

Patients who have been discharged and are unable or choose not to attend their Day 90 follow-up visit at the research site, may have their adverse event and concomitant medication data collected remotely via telephone. In these circumstances, patient questionnaires (EQ-5D-5L and HRUQ) may also be completed via telephone, or alternatively posted to the patient for completion.

## 6.3 Eligibility Confirmation

Once a patient has been screened and has had all eligibility assessments performed (see Section 5 and Section 6.1) and Informed Consent has been obtained (see Section 6.2), a doctor authorised on the site Delegation Log must confirm full eligibility of the patient. A record of this confirmation must be made in the patient’s medical notes on the date of screening.

## 6.4 Baseline

**N.B. Informed consent must be obtained before baseline as trial-specific data and blood samples are required as part of baseline assessment.**

Once the patient (or their Legal Representative) has provided written informed consent and a doctor authorised on the trial site Delegation Log has confirmed full eligibility, the baseline assessments will be undertaken. The weight of the consented patient will be determined through the use of a weighing scale, used to calculate trial treatment dosage. Any type of hospital weighing scale is appropriate for this purpose, depending on site availability and mobility of the patient. A qualified medical practitioner or non-medical/supplementary healthcare professional, such as an Advanced Nurse Practitioner or Pharmacist, authorised on the trial site Delegation Log will complete a signed prescription for the trial intervention. For non-medical/supplementary prescribers, their approvals must be maintained within their training files. Randomisation will be carried out by a member of the research team delegated the duty on the delegation log. Pharmacy, or unblinded research team responsible for preparation of trial medication in a separate biologics preparation area, will be made aware of the eligible patient so that they know to expect the treatment allocation email and can allocate time to prepare the trial treatment.

Further baseline assessments to be completed while trial medication is being prepared are:

1. A blood sample to determine Hepatitis B surface antigen and Hepatitis B core antibody status with results to be obtained after randomisation and administration of trial intervention;
2. Omics blood samples (2.5 ml in each of two PAXgene^®^ tubes for ribonucleic acid, RNA; 5 ml clotted blood in serum tube (centrifuged) for cytokines; 6 ml in acid citrate dextrose (ACD) tube for peripheral blood mononuclear cells, PBMCs; 8.5 ml in PAXgene tube for deoxyribonucleic acid, DNA; volumes subject to availability of tubes of these volumes);
3. Abdominal pain (numerical rating scale of pain in stomach area and opiate morphine equivalents received over last 24 hours);
4. Time of last solid food, history of vomiting;
5. Selective SOFA score (cardiology, respiratory and renal only);
6. Recording of ward area;
7. Concomitant medications (if any change from screening).

At any convenient point following administration of trial treatment the aetiology of AP should be recorded, including that informed by transabdominal ultrasound, alcohol history with use of the Alcohol Use Disorders Identification Tool (AUDIT) assessment tool, serum triglycerides, serum calcium and endoscopic ultrasound. The findings of any SARS-CoV-2 tests should also be recorded.

## 6.5 Randomisation Procedures

Participants will be randomised by a delegated member of the research team to receive either arm A infusion of 5 mg/kg infliximab or arm B 10 mg/kg infliximab or arm C placebo (in a ratio of 1:1:1) once*:

a. Eligibility criteria have been fulfilled;
b. Fully informed written consent has been obtained;
c. Full eligibility has been confirmed by an authorised doctor;
d. Baseline patient weight has been determined.
e. The prescription has been completed for the trial infusion and accompanying medications by an authorised doctor or non-medical/supplementary healthcare professional.

*Please see 3.1 Protocol modification following interim analysis, regarding discontinuation of arm B.

A delegated member of the research team will randomise participants by means of a secure, 24-hour web-based randomisation system stratified by centre using a programme controlled centrally by the LCTC. A personal login username and password, provided by the LCTC, will be required to access the randomisation system; designated research staff will be issued with their personal login and password.

When patient consent and full eligibility are confirmed in the system, a unique study number (randomisation number) will be displayed on a secure webpage and an automated email confirmation providing the treatment allocation will be sent to the Pharmacy, or the unblinded research team responsible for preparation of trial medication in a separate biologics preparation area, and the LCTC randomising statistician(s). The site’s PI, LCTC Trial Manager and the research staff member responsible for randomisation will receive a separate email confirming that randomisation has taken place without revealing treatment allocation. Administration of the trial treatment must be begun within 36 hours of admission, allowing 120 min within the 36 hour period for preparation of trial medication. It is the responsibility of the PI or delegated research staff to inform the Pharmacy, or the unblinded research team responsible for IMP preparation at their centre prior to randomisation to ensure there is sufficient supply of the study treatments.

**Randomisation: web access http://ctrc.liv.ac.uk/Randomisation/Rapid.One**

*If there are any problems with the randomisation systems, contact*

*the coordinating LCTC on 0151 794 9774, or via email on rapid.one@liverpool.ac.uk*

(Note that the coordinating LCTC is open from 0900 – 1700, Monday – Friday, excluding public holidays)

Centres will be provided with emergency back-up randomisation envelopes to be used in the event of a failure outside LCTC office hours or if the problem cannot be resolved in a reasonable timeframe. These will be stored in the Pharmacy, or separate biologics preparation area at each individual site. In the event that emergency back-up envelopes are required to be used, the Pharmacist or unblinded research team responsible for preparation of trial medication will be contacted by the blinded research team who will request randomisation of the patient. The Pharmacist, or unblinded research team responsible for preparation of trial medication will select the next sequentially numbered envelope that will give the randomised allocation. Instructions to be followed are provided in the envelopes. Upon receipt at LCTC, there will be a check to ensure the envelopes have been used in sequence. The site should also email the Trial Manager within 24 hours to notify LCTC that it has been necessary to use a back-up envelope.

Pharmacy, or the unblinded research team responsible for preparation of trial medication, will check to ensure that the correct number of randomisation envelopes are present, that they are intact, and that the sequential numbering system is maintained. Any discrepancies should be immediately reported to the LCTC.

## 6.6 Who is Blinded to Allocations

The participants, LCTC staff (excluding statistical team members as appropriate, those who process site dispensing logs or those involved in safety reporting), and all members of the site research teams administering trial medication and managing trial patients (including the delegated member randomising the patient but excluding Pharmacy, or the unblinded research team responsible for preparation of trial medication) will be blinded to treatment allocations.

Once the delegated blinded research team member performs randomisation and the Pharmacy, or unblinded research staff member responsible for preparation of trial medication, is provided with the allocation to either Arm A, B or C, the Pharmacy or unblinded research staff member will prepare the infusion, which will be covered by an opaque sleeve and labelled so as to maintain blinding.

Piperacillin/tazobactam (or the local centre’s antibiotic(s) of choice for intra-abdominal infections as defined in the recruiting centre’s written antibiotic policy e.g. if the patient has penicillin allergy), hydrocortisone and chlorphenamine will be prescribed for all trial patients and given to ensure equipoise between trial arms and to maintain blinding. Adrenaline will also be prescribed for use as required to counter severe anaphylactic reactions/shock from the infusion, should this occur, and paracetamol to be used as required. Severe anaphylactic reactions/shock are to be managed according to the Resuscitation Council (United Kingdom, UK) guidelines.

The IDSMC (and the trial statistical team) will be the only body with access to unblinded comparative data, which it will monitor to advise the Trial Steering Committee (TSC) on continuation or cessation of the trial.

For unblinding procedures see Section 8.6.2.

# 7 Participant Timeline, Procedures and Assessments

## 7.1 Schedule for Follow-up

The total duration of the study is 90 days from commencement of trial infusion, which must be administered as a single dose infusion starting within 36 hours of admission. Assessments will be conducted in line with the schedule in the table below. If patients are discharged before assessment time points are reached, they will be assessed as outpatients.

Assessment on Day 90 (+/- 7 days) may be undertaken remotely by videolink or telephone, if after discharge a participant is unable or would prefer not to return to the research site for their visit, but not on Days 4 (+/- 1 day) or 14 (+/- 2 days), as these are critical to the primary outcome and patients need to attend the research site in person.

**Table 3. Schedule of Assessments.**

| Procedures | | Screening | Consent | Baseline / Randomisation | IMP Administration | As required from trial entry | Day 2 (12-36 h) | Day 4 (+/- 1 day) | Day 14 (+/- 2 days) | Day 90 (+/- 7 days) |
| --- | --- | --- | --- | --- | --- | --- | --- | --- | --- | --- |
| Diagnostic blood tests (amylase, lipase) | | X |  |  |  |  |  |  |  |  |
| Assessment of diagnosis of acute pancreatitis | | X |  |  |  |  |  |  |  |  |
| Review of medical history | | X |  |  |  |  |  |  |  |  |
| Review of concomitant medications ^A^ | | X |  |  |  | X |  |  |  | X |
| Physical exam | Complete | X |  |  |  |  |  |  |  |  |
|  | Symptom-directed (including vital signs) | X |  |  |  | X |  |  |  |  |
| Local blood tests and results ^B^ | Full blood count and differential | X |  |  |  | X | X | X | X |  |
|  | Creatinine | X |  |  |  | X | X | X | X |  |
|  | Albumin | X |  |  |  | X | X | X | X |  |
|  | Calcium | X |  |  |  | X |  |  |  |  |
|  | Glucose | X |  |  |  | X |  |  |  |  |
|  | Triglycerides | X |  |  |  | X |  |  |  |  |
|  | PaO2/SpO2 ^C^ | X |  |  |  | X |  |  |  |  |
|  | Hep B surface antigen + core antibody assay |  |  | X |  |  |  |  |  |  |
| Central blood sample collection, processing and posting using collection kits provided ^B^ | CRP ^D^ |  |  |  |  |  | X | X | X |  |
|  | Omics blood samples ^E^ |  |  | X |  |  | X | X | X |  |
|  | Measurement of infliximab levels |  |  |  |  |  |  | X | X |  |
|  | Detection of antibodies to infliximab |  |  |  |  |  |  |  | X |  |
| Pregnancy test (urine) | | X |  |  |  |  |  |  | X |  |
| ECG | | X |  |  |  |  |  |  |  |  |
| Chest x-ray | | X |  |  |  |  |  |  |  |  |
| Assessment of eligibility criteria | | X |  |  |  |  |  |  |  |  |
| Signed consent form | |  | X |  |  |  |  |  |  |  |
| Confirmation of full eligibility by an authorised doctor | |  |  | X |  |  |  |  |  |  |
| Patient weight | |  |  | X |  |  |  |  |  |  |
| Randomisation | |  |  | X |  |  |  |  |  |  |
| Prescription of piperacillin/tazobactam ^F^, hydrocortisone, chlorphenamine, adrenaline and trial infusion | |  |  | X |  |  |  |  |  |  |
| Preparation of trial infusion | |  |  | X |  |  |  |  |  |  |
| Administration of piperacillin/tazobactam or alternative(s) ^G^ | |  |  | X |  | X |  |  |  |  |
| Administration of hydrocortisone and chlorphenamine | |  |  | X |  |  |  |  |  |  |
| Administration of trial infusion | |  |  |  | X |  |  |  |  |  |
| Assessment of Adverse Events ^A^ | |  |  |  | X | X |  |  |  | X |
| Daily assessment of abdominal pain ^H^ | |  |  | X |  | X | X | X | X |  |
| Daily recording of oral solid food intake ^I^ | |  |  | X |  | X | X | X | X |  |
| Daily selective SOFA score ^J^ | |  |  | X |  | X | X | X | X |  |
| Daily recording of ward area | |  |  | X |  | X | X | X | X |  |
| Recording of imaging and interventions | |  |  |  |  | X |  |  |  | X |
| Aetiology ^K^ | |  |  |  |  | X |  |  |  | X |
| Contrast enhanced pancreatic CT scan ^L^ | |  |  |  |  |  |  |  | X |  |
| EQ-5D-5L ^A^ | |  |  |  |  |  |  | X | X | X |
| HRUQ ^A M^ | |  |  |  |  |  |  |  | X | X |
| Data Collection ^A^ | | X | X | X | X | X ^N^ | X | X | X | X |

Assessments for Day 2 and Day 4 must take place 12-36 h and 48-120 h respectively after commencement of trial infusion (defined as 0 h).

^A^ Day 90 follow up can be completed remotely via telephone (including questionnaires).

^B^ Consent is mandatory before collection of any blood samples not part of standard care.

^C^ Either pulse oximetry oxygen saturation or arterial blood gases recorded at admission on air.

^D^ 5 ml clotted blood in SST tube (centrifuged) for CRP

^E^ 2.5 ml in x 2 PAXgene tubes for RNA; 5 ml in SST tube (centrifuged) for cytokines; at baseline: 8.5 ml in PAXgene tube for DNA; at baseline, Day 4 and 14: 6ml in ACD tube for PBMCs; volumes subject to availability of tubes of these volumes.

^F^ Alternative antibiotics (e.g. if penicillin allergy) for intra-abdominal infection as defined in local written antibiotic policy.

^G^ Administered for up to 7 days or until discharge, whichever time point is earlier; alternatives given in Section 6.6.

^H^ Daily numerical rating scale of abdominal pain and daily administration of opiates during inpatient stay for up to 14 days.

^I^ For nutritional assessment see Section 7.1.1, recorded during inpatient stay for up to 14 days.

^J^ Cardiac, respiratory and renal only; if no lab test, most recent value from previous day to be used; up to 14 days as ^H, I^.

^K^ Transabdominal ultrasound, alcohol (AUDIT) and drug history, serum triglycerides, calcium, +/- endoscopic ultrasound +/- magnetic resonance cholangiopancreatography (MRCP).

^L^ Contrast enhanced computed tomography scan (CECT) on Day 14 +/- 7 days.

^M^ Health Resource Use questionnaire (HRUQ, to be completed if patient has been discharged from index (trial) admission).

^N^ Data collection on inpatients up to discharge or Day 14, whichever is soonest as for ^H, I, J^.

The following activities will occur at each of the time points with every effort made for these activities to be conducted on the day as specified and if not then within the time window specified.

### 7.1.1 Continuous Recordings

All participants administered a trial infusion are to be observed for at least 1-2 hours post-infusion for acute infusion-related reactions. Symptom directed physical examination, abdominal pain (daily numerical rating scale and administration of opiates), nutrition and route (oral intake of solid food) and selective SOFA score (cardiac, respiratory and renal only; if no lab test, using most recent value from previous day) will be recorded daily until Day 14 or discharge, whichever is soonest. Imaging and therapeutic interventions (e.g. mechanical ventilation, drainage, necrosectomy) and ward area (A&E, Level 3/General Ward, Level 2/High Dependency Unit, Level 1/Intensive Care Unit, other) will be recorded until Day 90 or discharge, whichever occurs soonest.

Piperacillin/tazobactam should be administered for up to 7 days or until discharge, whichever time point is earlier. Alternative antibiotics are given in [Section 6.6](#_Toc439170066) and Section 8.9.1. Concomitant medications (drug and duration, see Section 8.9.3 for required information) and adverse events will be collected from screening until Day 90 (during the in-patient stay and at subsequent follow up visits).

All routine blood tests (full blood count and differential, creatinine and liver function tests [only albumin to be reported]) in the tabulated schedule (routine admission assessment and on Days 2, 4, and 14, or nearest day after trial infusion for each time point) will be conducted by the clinical team and results used by the clinical team. The primary outcome measurement of CRP will be measured in samples taken on Days 2, 4 and 14 (using sample collection kits provided), with the CRP assay undertaken centrally to ensure standardised measurement. The clinical team will not have access to the results of the centralised assay of CRP for clinical management but may assay CRP locally at any time if considered clinically necessary. Local CRP results nearest in time (days) to any missing central CRP data may be requested if centrally analysed CRP is unobtainable.

Aetiology will be recorded when available either during the in-patient stay or follow-up period until Day 90, including the findings of any SARS-CoV-2 tests and any treatment given for COVID-19; severity classification (RAC^32^) will also be made during this period.

### 7.1.2 Day 2 (12-36 h after commencement of trial infusion)

Full blood count, differential and liver function tests (only albumin to be reported) undertaken locally; CRP (centrifuged) and Omics blood samples will be taken at this time point for assessment at a central laboratory (using sample collection kits provided).

### 7.1.3  Day 4 (+/- 1 day i.e. 48-120 h after commencement of trial infusion)

Full blood count, differential and liver function tests (only albumin to be reported) undertaken locally; CRP (centrifuged), Omics (including ACD for PBMCs) and infliximab level blood samples will be taken at this time point for assessment at a central laboratory (using sample collection kits provided). Participants will also be asked to complete the EQ-5D-5L questionnaire.

### 7.1.4 Day 14 (+/-2 days)

Full blood count, differential and liver function tests (only albumin to be reported) undertaken locally; CRP (centrifuged), Omics (including ACD for PBMCs), infliximab level and antibodies to infliximab blood samples will be taken at this time point for assessment at a central laboratory (using sample collection kits provided). Women of childbearing potential will have a urine pregnancy test. Patients will be asked to complete the EQ-5D-5L and, if already discharged from hospital, an HRUQ questionnaire. A CECT scan will also be performed to assess local pancreatic injury; these scans will be available to be read locally but will be assessed by a centralised panel as a secondary measure of trial outcome. As one or more CECT scans are undertaken at varying time points in 60% patients with AP as standard of care in the UK^62^, and early repetition of CECT is usually unnecessary, the window for CECT is wider at Day 14 +/- 7 days. If two or more CECTs are undertaken for clinical reasons in any individual patient, the nearest to Day 14 will be used for central trial evaluation.

### 7.1.5 Discharge

Date of discharge will be recorded.

### 7.1.6 Day 90 (+/-7 days)

This follow up visit can be undertaken remotely by videolink or telephone if participants are unable or would prefer not to attend the hospital. Participants will be asked to complete the EQ-5D-5L questionnaire and, if discharged from their index (trial) admission, an HRUQ; both can be undertaken remotely over videolink or by telephone, or if not suitable posted to the participant to complete and return. Date and length of in-patient stay of any readmission(s) during the first 90 days will be recorded as well as reason(s), intervention(s) and outcome. Women of childbearing potential will be asked if they have become pregnant since entering the trial.

### 7.1.7 Beyond Day 90

Data on the length of in-patient stay beyond 90 days during the index admission (at the start of which trial medication was given) will not be obtained as part of this protocol but may be sought subsequently as well as reason(s), intervention(s) and outcome.

## 7.2 Efficacy

Efficacy will be determined by comparison of the cumulative CRP (AUC) between trial treatment arms. Further exploratory efficacy analyses will be comparison between trial treatment arms of the secondary outcome measures, all of which represent the disease and capture severity. Comparison of the time course of immune response gene expression and serum cytokines (including TNFα and Interleukin-6) will also be made between treatment arms.

Level of care, investigations, treatments, length of stay and resource use will be recorded in trial participants’ Case Report Forms (CRFs) and via the Finance Departments of participating centres’ provision of patient-level information and costing system (PLICS) data. Unit costs will be obtained from standard sources. Quality-adjusted life years (QALYs) will be estimated as AUC, using data from the EQ-5D-5L questionnaire administered on Days 4, 14 and 90.

The primary outcome measure is serum CRP (clotted blood in SST tube, centrifuged, for serum) measured at Days 2, 4 and 14 time points with measurement in a central laboratory.

**N.B. Centrifugation is mandatory at the research site, prior to posting, to ensure separation of serum from clotted cells by gel to prevent degradation of CRP.**

CRP rises to a peak then falls slowly for a substantial period in all patients with AP (see Figure 3 in Section 2.2); CRP is a clinically useful marker of disease outcome that is the most studied and robustly validated marker in AP^12,14,20,21^. CRP is used to decide when imaging or intervention is necessary and as a means to determine progression or resolution of AP; CRP levels fall in response to successful intervention^39^. BRU prospective data on a 330 patient cohort matching the inclusion and exclusion criteria of RAPID-I confirm CRP rises in all patient groups to a level and for a period proportional to severity (classified according to the RAC): in mild AP to 80 +/- 10 mg/L (M +/-SEM), elevated for two weeks; moderate to 220 +/- 30 mg/L, elevated for four weeks; severe to 320 +/- 40 mg/L, elevated longer. Changes in CRP over time can be represented by the area under the curve (AUC), which the BRU cohort shows to be directly proportional to disease outcome (RAC severity; r=0.637, p<0.001), notably the presence and duration of organ failure.

Should the centrally analysed CRP be unavailable then sites may be requested to provide local CRP results nearest in time (days) to any missing central CRP data.

The secondary outcome measures will be pain (cumulative numerical rating scale and opiate administration in morphine equivalents for the first 14 days while an inpatient), nutritional deficit (number of days without solid food up to Day 14), rise in neutrophils (AUC for first 14 days), decline in serum albumin (negative AUC from admission value for first 14 days), respiratory, cardiac and/or renal dysfunction assessed by cumulative serial organ failure assessment (SOFA up to Day 14) score, local pancreatic and associated injury on pancreas contrast-enhanced computerised tomography scan (CECT on Day 14 +/- 7 days), infective complications (first 90 days), length of hospital stay, mortality (first 90 days), and patient reported outcome (EQ-5D-5L^56^) at Days 4, 14 and 90. Where possible cumulative measures will be used, as with serum CRP, to reflect the overall impact of disease outcome. A blinded panel of pancreatic radiologists at the Royal Liverpool University Hospital will assess the extent of injury seen on CECT on Day 14 (+/- 7 days) using the modified CT severity index that obtains a score of up to 10 for inflammation, fluid collections, necrosis and extra-pancreatic complications^63^. EQ-5D-5L will be used to calculate the incremental cost per QALY gained through trial treatment (see Section 7.4.1 below). The measures have been confirmed in the BRU cohort to display abnormality in all patient groups, proportional to severity and clinical outcomes.

## 7.3 Procedures for Assessing Safety

Each trial participant’s baseline data will assist identification of any adverse event, whether or not related to infliximab administration, distinct from any adverse reaction where there may be a relationship with infliximab. There will be monitoring during the infliximab infusion, and a member of the trial team will review trial participant in-patients daily. If discharged, participants will be asked whether they have experienced any adverse events at return visits (or remotely at Day 90 +/- 7 days) until study completion at the Day 90 time point. The needs of each participant will determine further referral (e.g. to Infectious Diseases Physician^43^) and standard follow-up for AP.

Exploratory safety analyses will be undertaken using transcripts selected to assess (1) innate immune responses; (2) changes in T cell subsets and their functions; (3) other changes contributing to immune competence, complemented by cytokine profiling.

### 7.3.1 Safety Assessment 1

Hepatitis B surface antigen and core antibody will be assayed after trial entry^43^; standard liver function tests (albumin, bilirubin, alanine aminotransferase/aspartate aminotransferase, alkaline phosphatase, gamma glutamyl transferase) will be monitored locally, at participating sites, as part of standard care, throughout the trial.

### 7.3.2 Safety Assessment 2

Adverse events related to infliximab including infusion reactions and delayed serum sickness reactions (that typically occur after a second or subsequent infliximab infusion that will not to be given in this trial, but the reaction may still occur) will be reviewed during and subsequent to trial treatment up to Day 90 days.

### 7.3.3 Safety Assessment 3

Antibodies to infliximab will be measured at Day 14.

### 7.3.4 Safety Assessment 4

The number of infective complications in participants receiving infliximab at either dose will be compared with the number of infective complications in participants receiving placebo. Data on the number of infective complications in each treatment arm will be presented at each IDSMC meeting during the conduct of the trial (made available by the Trial Statistician and seen only by the IDSMC, see Section 10.4) and analysed when the trial is complete (see Section 10.5).

### 7.3.5 Safety Assessment 5

Exploratory (discovery) safety analyses will be undertaken using transcripts selected to assess (1) innate immune responses; (2) changes in T cell subsets and their functions; (3) other changes contributing to immune competence, complemented by cytokine profiling.

### 7.3.6 Safety Assessment 6

Urine pregnancy tests at baseline and the Day 14 time point will be performed for all female participants of childbearing potential. Pregnancy of female partners of male trial participants will not be assessed or reported as part of the trial. Most importantly patients are required to attend for trial follow up on Day 14 +/- 2 days for the primary outcome measure, but if they do not, the local research team will post a pregnancy test to the patient for them to complete. The result of the completed pregnancy test will be communicated to the local research team by the patient and the CRF updated accordingly.

## 7.4 Other Assessments

### 7.4.1 Health Economics

A cost utility analysis will be conducted to estimate the incremental cost per quality-adjusted life year (QALY) gained with infliximab (optimal dose) versus placebo (standard care).

The direct costs of health care resources used by patients in the trial after discharge from their index (trial) admission will be calculated from data collected using an HRUQ and administered by the research nurse (RN) at Days 14 and 90 (if and when patient discharged from their index (trial) admission). Where patients have been discharged and are unable and would prefer not to return to the research site for their Day 90 trial visit, their questionnaires can be completed remotely by videolink or telephone, or if not suitable, via the post.

The questionnaire includes items related to use of primary care services, A&E, inpatients stays and outpatient clinic visits. Participants’ use of secondary care services while admitted will be recorded directly using the study CRFs. These will be supplemented with PLICS data, obtained subject to agreement from the Finance Departments of each participating centre. These datasets include Health Resource Groups which detail costs for patient stays and treatments. Responsibility for the data collection and pseudonymisation will rest with the site RN who will supply their site Finance Departments with the necessary details to ensure only information on consented participating patients are provided. It is the responsibility of the site Finance Departments to provide the site RNs with the data in a timely fashion and should the site RN so request, to ensure all patient identifying data have been replaced with the patient RAPID-I trial number. Pseudonymised PLICS data will be transferred securely from each site RN to the LCTC, and then on to the Centre for Health Economics and Medicines Evaluation, Bangor University for analysis.

QALYs will be estimated from patients’ responses to the EQ-5D-5L questionnaire, administered on Days 4 (only if the patient is conscious and able to), 14 and 90. The five attributes of this questionnaire (mobility, self-care, usual activities, pain/discomfort, and anxiety/depression) will be summarised into a single preference-based utility score. EQ-5D-5L data will be analysed in accordance with the NICE preferred approach as indicated at the time of analysis.

### 7.4.2 Special Assays or Procedures

Transcriptomic (2.5 ml blood x 2 in PAXgene tubes) and cytokine (5 ml clotted blood in SST tube (centrifuged) for serum, in addition to separate 5 ml clotted blood in SST tube (centrifuged) for the primary outcome measure CRP) samples will be taken at baseline and at Days 2, 4 and 14 time points. Additionally, 8.5 ml PAXgene tube blood sample for DNA will be taken at baseline only, and samples will be taken at baseline, Days 4 and 14 for PBMC profiling (6 ml in ACD; all volumes subject to availability of tubes of these volumes).

**N.B. Trial samples (including those for CRP at the specified time points) will be packaged into kits (supplied by UK Biocentre) and when possible *sent on the same day* and if not then *as promptly as possible thereafter* to the UK Biocentre (NIHR NBC, Units 2&3, Java Park, Bradbourne Drive, Tilbrook MK7 8AT, UK)**.

Subsequently NanoString technology will be used for quantitative transcriptomic analyses, with eQTL analysis from Illumina genome-wide association study of patient DNA, assessing a panel of 100 gene transcripts defined from the BRU whole transcriptome cohort study at the time points specified. The transcripts have been selected from all those differentially expressed between mild and severe disease, with 20 transcripts representing each of (1) the largest and most consistent differential expression between mild and severe disease, including a prognostic signature; (2) TNFα and downstream signaling; (3) innate immune responses, notably those of neutrophils typical of AP; (4) changes in T cell subsets and their functions; (5) other changes contributing to immune competence. This will be complemented by Luminex cytokine profiling of TNFα, interleukin 6 (IL6), IL2, total interferon alpha and gamma, and by peripheral blood mononuclear cell subset mass cytometry (Cytometry by time of flight, CyTOF) in all participants.

Participants will be asked if they are happy to consent to any remaining samples being kept for use in future research studies (this will be optional to patients). Samples will be stored at the UK Biocentre for future and exploratory measurements.

## 7.5 Patient Transfers

A number of participants recruited at secondary care centres may require specialist pancreatic care and referral to a tertiary care centre may be required. The research team responsible for the patient at the secondary care centre must liaise with the research team at the tertiary care centre to ensure they are aware of the patient and are able to continue trial follow-up assessments and procedures.

For participants who are transferred for any other reason (e.g. they move from the area in which they were originally recruited to the trial), every effort should be made for them to be followed-up at another participating trial centre and for this trial centre to take over responsibility for the participant or for follow-up via the participant’s GP.

For all transfers, a transfer case report form (CRF) will be completed by the current centre and returned to LCTC. LCTC will then forward the transfer CRF on to the new centre, the PI at the new centre will be asked to sign this CRF and return to LCTC to confirm that they are taking over responsibility for the patient in the trial. The current centre should provide a copy of the participant’s full CRF to the new centre.

## 7.6 Withdrawal

In consenting to the trial, participants are consented to trial treatment, follow-up and data collection. Participants (or their Legal Representatives) are free to withdraw consent at any time without providing a reason. If voluntary premature discontinuation of treatment occurs, the participant (or Legal Representative) should be asked to allow continuation of scheduled evaluations and be given appropriate care under medical supervision until the symptoms of any adverse event resolve or the participant’s condition becomes stable.

Follow-up of these participants will be continued through the trial research nurses, the PI at each centre and, where these are unsuccessful, through the participant’s General Practitioner (GP, via the site), unless the participant explicitly also withdraws consent for follow-up.

### 7.6.1 Premature Discontinuation of Trial Intervention

Participants may prematurely discontinue trial treatment for any of the following reasons:

a. Withdrawal of consent.
b. Unacceptable toxicity.
c. Intercurrent illness preventing further treatment.
d. Any change in the participant’s condition that justifies the discontinuation of treatment in the attending clinician’s opinion.

If a participant wishes to prematurely discontinue trial treatment, centres should nevertheless explain the importance of remaining on trial follow-up, or failing this, of allowing routine follow-up data to be used for trial purposes. Generally, follow-up will continue unless the patient explicitly also withdraws consent for follow-up (see Section 7.6.2).

### 7.6.2 Withdrawal from Trial Completely

Those who wish to withdraw consent for the trial completely will have pseudonymised data collected up to the point of that withdrawal included in the analyses, however the participant will not contribute further data to the trial unless required by law. The LCTC must be informed in writing and a withdrawal CRF should be completed.

## 7.7 Loss to Follow-up

The minimum follow-up will be until Day 90 from the time of recruitment of the last participant. If any of the trial participants are lost to follow-up, contact will initially be attempted through the local PI or delegated research staff at each site. Wherever possible, information on the reason for loss to follow-up will be recorded.

In the case of a missed assessment visit, the scheduled measurements at the next visit should be carried out as planned and the lost data due to the missed visit will be considered as missing values.

## 7.8 Trial Closure

The end of the trial is defined in 7.8.1. The TSC, however, may close the trial prematurely on the recommendation of the IDSMC.

### 7.8.1 Definition of End of Trial

The end of the trial is defined as the date on which data for all participants are frozen and data entry privileges are withdrawn from all trial databases.

# 8 Trial Treatment

## 8.1 Introduction

Infliximab is a prescription drug with marketing authorisation for the treatment of rheumatoid arthritis, Crohn's disease, ulcerative colitis, ankylosing spondylitis, psoriatic arthritis and psoriasis. In the RAPID-I trial infliximab will be used outside the manufacturer’s indication for the treatment of AP, and it is classed as an investigational medicinal product (IMP).

Participants will be randomised equally (Section 6.5) to infusion with 5 mg/kg infliximab (arm A, Section 8.2), infusion with 10 mg/kg infliximab (arm B, Section 8.3) or infusion of 0.9% sodium chloride (arm C placebo, Section 8.4).

Compulsory administration of non-investigational medicinal products (NIMPs) before and after the IMP/placebo infusions is described in Section 8.9.1.

Please refer to the relevant current Summary of Product Characteristics (SPC) for infliximab 100 mg powder for concentrate for solution for infusion, available on the Electronic Medicines Compendium (eMC) website: <https://www.medicines.org.uk/emc/product/3831/smpc> for infliximab (detailed in Section 8.2.1)... Additional information is also available in the RAPID-I Pharmacy Manual/IMP Preparation Manual, which also describes arrangements required at those centres where trial medication will not be prepared and dispensed by Pharmacy e.g. a Clinical Research Facility or suitable alternative.

## 8.2 Arm A - Infusion of 5 mg/kg Infliximab

### 8.2.1 Formulation, Packaging, Labelling, Storage and Stability

**Generic name:** Infliximab (Any available brand)

**Supply and distribution:** Vials of infliximab will be sourced from existing hospital stock via usual NHS procurement procedures. The research team will liaise with the Pharmacy team to ensure that there are always enough vials of infliximab in stock and in date.

**Packaging:** As commercially packaged and provided by manufacturer.

**Formulation:** Each vial contains infliximab 100 mg powder in the form of freeze-dried white pellet, for concentrate for solution for infusion.

**Labelling:** Label templates for the Infliximab vials will be provided. Site Pharmacies will be required to print labels and label the vials at the point of dispensing. Site specific changes to the provided template should be agreed by Sponsor prior to use. Each vial will be labelled and used for RAPID-I trial use only and the label will be in line with regulation 46 SI2004/1031 and the detailed guidance provided in annex 13 of the European Union (EU) Good Manufacturing Practice (GMP) guide.

**Storage:** Infliximab should be stored in a refrigerator at 2 °C to 8 °C and can be stored for up to 3 years, or less as per expiry/re-test date. Upon removal from refrigerated storage, infliximab must not be returned to refrigerated storage. *N.B. Storage instructions are for infliximab before reconstitution.*

**Stability:** Once reconstituted, the infliximab solution should be used as soon as possible and within 3 hours of reconstitution and dilution. When reconstitution and dilution are performed under aseptic conditions, infliximab infusion solution can be used within 24 hours if stored at 2 °C to 8 °C. Do not store any unused portion of the infusion solution for re-use.

### 8.2.2  Dosage, Preparation and Administration of Study Treatment/s

**Dosage:** 5 mg/kg is 5 mg of infliximab per kg of body weight. Each infliximab vial contains 100 mg infliximab. The participant’s weight will be detailed on the prescription and the trial Pharmacy, or unblinded research team staff responsible for preparation of trial medication, will calculate the appropriate dose and number of vials needed for that participant. As anaphylactic shock is a possible infusion reaction of infliximab, emergency resuscitation equipment should be available in the ward area within which the administration of study treatment is conducted, including an artificial airway.

**Preparation:** Reconstitute and dilute in accordance with the relevant current manufacturer’s SPC, which is available on the eMC website: (<https://www.medicines.org.uk/emc/product/3831/smpc>). Site teams should access the SPC for the infliximab brand being used at their site.

**N.B. For participants who weigh under or equal to 100 kg, please make up into a total volume of 250 ml with 0.9% sodium chloride. For patients who weigh over 100 kg and up to 200 kg, please make up into a total volume of 500 ml with 0.9% sodium chloride. This will ensure the concentration of infliximab infusion will not exceed 4mg / ml, as per manufacturer’s SPC**.

**Reconstitution**: Please refer to the RAPID-I Pharmacy Manual/IMP Preparation Manual for instruction.

**Administration:** Diluted infliximab in 0.9% sodium chloride:

1. Should be administered intravenously and initiated within 36 hours of admission. The infusion should be started as soon as possible once provided by the Pharmacy, or unblinded research team responsible for preparation of trial medication (this must be within 3 hours of reconstitution and dilution, or with 24 hours if stored under refrigerated conditions 2°C - 8°C)
2. Over a 2-hour period.
3. Via an infusion pump, using an infusion set with an in-line, sterile, non-pyrogenic, low protein-binding filter (pore size 1.2 micrometer or less).
4. Qualified healthcare professionals (authorised by the PI) trained to detect any infusion-related issues should administer the infusion.
5. All participants administered infliximab are to be observed for at least 1-2 hours post-infusion for acute infusion-related reactions.

### 8.2.3 Specific Restrictions

The effect of co-administration of infliximab with other agents is unknown. Do not infuse infliximab concomitantly in the same intravenous line with other agents.

### 8.2.4 Overdose

Overdose may occur if the wrong patient weight has been supplied to the Pharmacy, or unblinded research team responsible for preparation of trial medication, an incorrect weight is used in the calculation, or the calculation has been done incorrectly. As per REMICADE^®^ 100 mg powder for concentrate for solution for infusion SPC, single doses up to 20 mg/kg have been administered without toxic effects. Special care must be taken by all site team members to avoid overdosing, notably by the Pharmacy, or unblinded research team responsible for preparation of trial medication, who are most likely to identify an overdose has been prepared. In the event of identification of an overdose, unblinding should occur, the patient will be closely observed for any adverse event and the site team should notify the LCTC of the event and examine the reasons for the event occurring to ensure that it does not happen again. The LCTC will log this event as a protocol deviation.

## 8.3 Arm B – Infusion of 10 mg/kg Infliximab

### 8.3.1 Formulation, Packaging, Labelling, Storage and Stability

See Section 8.2.1.

### 8.3.2 Dosage, Preparation and Administration of Study Treatment/s

**Dosage:** 10 mg/kg is 10 mg of infliximab per kg of body weight. Each IMP vial contains 100 mg infliximab. The participant’s weight will be detailed on the prescription and the trial Pharmacy, or unblinded research team staff responsible for preparation of trial medication, will calculate the appropriate dose and number of vials needed for that participant.

Preparation and administration are as described in Section 8.2.2.

### 8.3.3 Specific Restrictions

See Section 8.2.3.

### 8.3.4 Overdose

See Section 8.2.4.

## 8.4 Arm C – Placebo

250 mls (for participants weighing under or equal to 100 kg) or 500 mls (for participants weighing over 100 kg and up to 200 kg) commercial sodium chloride (0.9%) solution:

1. Should be administered intravenously and initiated within 36 hours of admission.
2. Over a 2 hour period.
3. Via an infusion pump, using an infusion set with an in-line, sterile, non-pyrogenic, low protein-binding filter (pore size 1.2 micrometer or less).

## 8.5 Dose Modifications/Interruption

If a mild reaction occurs to the administration of trial treatment (e.g. pruritus, flushing or myalgia) then the infusion does not need to be modified. If a moderate reaction occurs (e.g. chest tightness, urticaria or hypertension), the infusion should be interrupted and chlorphenamine i.v. (if not already administered, or a further dose of 10 mg if 10 mg has already been administered) and/or paracetamol should be administered. If there is a favourable response following this, then the infusion can be resumed. If there is a severe reaction then the infusion should be immediately stopped and emergency management should take place with adrenaline, resuscitation and intensive support, according to the Resuscitation Council (United Kingdom, UK) guidelines.

## 8.6 Blinding and Unblinding

### 8.6.1 Blinding

Infusion bags for IMP/placebo treatment will be covered in an opaque sleeve, sealed and labelled by the Pharmacy or unblinded research team responsible for preparation of trial medication for blinding, thus the staff/department preparing the trial medication is not blinded to the treatment allocation. The treatment allocation must not be revealed to the blinded research staff administering the infusion and managing the participant, unless unblinding becomes necessary at a subsequent time point (see Section 8.6.2 below). Any evidence of seal breakage or tampering observed by any member of the trial team should be recorded as accidental unblinding, as per Section 8.6.2.1.

### 8.6.2 Unblinding

*N.B. Allocation must not be routinely revealed to LCTC personnel.* Unblinding will generally be discouraged during treatment.

If simply ceasing trial treatment is a viable option for the participant’s care, it should not be necessary for unblinding to occur.

**Justification:** Unblinding is done on a per case basis (i.e. single participant) when knowing the treatment allocation is needed to:

1. Enable treatment of severe adverse event/s, or
2. Enable administration of another therapy that is contraindicated by the trial treatment, or
3. Enable appropriate ongoing care upon cessation of allocated trial therapy.

**Procedure:**

***During LCTC office hours (Monday-Friday 9 am-5 pm, excluding bank holidays):***

1. Site personnel should call the Trial Manager at LCTC who will contact the Chief Investigator (Professor Robert Sutton), to obtain approval, where possible.
2. Once approved, site personnel should then request the unblinding from the local site Pharmacy/unblinded research team (N.B. The PI is responsible for ensuring that all research personnel are aware of contact details for obtaining details of treatment allocation).
3. As soon as possible thereafter, the Trial Manager at LCTC should be contacted confirming unblinding has taken place and an unblinding CRF should be completed and returned LCTC.

***Out of LCTC office hours:***

Site personnel should request the unblinding from the local site Pharmacy/unblinded research team (N.B. The PI is responsible for ensuring that all research personnel are aware of contact details for obtaining details of treatment allocation), if Pharmacy is open/unblinded research team is available.

***Out of Pharmacy office hours/when unblinded research team is unavailable:***

Assume all patients are on infliximab and administer treatment accordingly.

**In all instances of unblinding:**

1. Do not disclose treatment allocation to site personnel unless knowledge is directly relevant to patient care.
2. Record and report in writing to the LCTC, by use of the unblinding CRF (including the identity of all recipients of the unblinding information).

**Please note, should an emergency situation occur, unblinding may occur without approval being in place from the CI and LCTC to enable management/treatment of patient.**

#### 8.6.2.1 Accidental Unblinding

If accidental unblinding occurs, this must be reported to the LCTC by use of the unblinding CRF. When reporting, include details about:

1. Date of unblinding;
2. Detailed explanation of circumstances;
3. Recipients of the unblinding information;
4. Action to prevent further occurrence.

#### 8.6.2.2 Unblinding at Trial Closure

Upon trial closure the criteria for unblinding will remain in effect. The Pharmacy or unblinded research team responsible for preparation of trial medication **should not** disclose treatment allocations on an individual basis.

Incidents of immunogenicity to even single low doses of infliximab are not uncommon; this is of concern because future use of the drug might be rendered ineffective. For this reason, at trial closure, the LCTC will notify local investigators in writing of unblinding information for patients under their care. A copy of this notification should be placed in the medical records and a copy retained in the Investigator Site File. It is the responsibility of the local PI to notify trial participants of their allocated treatment.

## 8.7 Accountability Procedures for Study Treatment

IMP is to be sourced from existing hospital stock via usual NHS procurement procedures. The research team will liaise with the Pharmacy team to ensure that there are always enough vials of infliximab in stock and in date. At any centre where trial medication is not prepared by Pharmacy, but in a separate biologics area e.g. a Clinical Research Facility, IMP stock will stored at the centre’s Pharmacy Department for subsequent recorded transfer of stock on an ongoing basis to the separate biologics preparation area for storage in accordance with IMP regulations, unless the Facility has the capacity to receive and store IMP stock directly, in accordance with IMP regulations.

## 8.8 Assessment of Compliance with Study Treatment

In all three arms, the full volume (250 ml, or 500 ml for all patients weighing over 100kg) of the treatment infusion should normally be administered over a period of 2 hours. Any defect in the opaque sleeve over the infusion bag or evidence of seal breakage or tampering observed by any member of the trial team should be recorded as accidental unblinding as per Section 8.6.2.1.

## 8.9 Concomitant Medications/Treatments

### 8.9.1 Non-Investigational Medicinal Products (NIMPs)

As NIMPs do not fall within the definition of investigational medicinal products, Articles 13 and 14 of Directive 2001/20/EC are not directly applicable. Therefore, these NIMPs should be dispensed and destroyed in accordance with local legislations and requirements. All NIMPs to be administered will be sourced via usual hospital procurement arrangements and stored in accordance with local hospital practice and in compliance with manufacturer’s instructions. All NIMPs prescribed to participants taking part in RAPID-I will be recorded on the “Concomitant Medications CRF”.

The following NIMPs are to be administered to **all participants** **before and continued after** the IMP/placebo infusion:

- piperacillin/tazobactam 4.5 g i.v three times a day (t.d.s) for up to 7 days or until discharge, whichever is earlier.
- as an alternative, centres may use one of their own antibiotic regimens for intra-abdominal infection as defined in their written local antibiotic policy (e.g. because of penicillin allergy) for up to 7 days or until discharge, whichever is earlier, and recorded in the trial CRF accordingly.

NIMPs to be administered to **all participants** **before** the IMP/placebo infusion are:

- 100 mg hydrocortisone i.v. (compulsory prophylactic), and
- 10 mg chlorphenamine i.v. (compulsory prophylactic).

NIMPs to be administered to participants as required are:

- chlorphenamine (further dose of 10 mg i.v.)
- paracetamol, and
- adrenaline, to be prescribed in advance of administration of trial IMP and used in the event of severe anaphylaxis according to the Resuscitation Council (UK) guidelines.

### 8.9.2 Medications Not Permitted/ Precautions Required

In the absence of compatibility studies, the trial medicinal product (infliximab or placebo) must not be infused with other medicinal products. The SPC for the infliximab brand used at site must be followed in all patients who are entered into the RAPID-I trial during the 90 days of follow-up, avoiding concurrent administration of other biological therapeutics, live vaccines and therapeutic infectious agents.

### 8.9.3 Data on Concomitant Medication

Concomitant medications data are to be collected from trial entry until study completion at Day 90 and recorded on the “Concomitant Medications CRF”. The details to be recorded are NIMPs administered including antibiotics prescribed outside of the protocol, opiates, inotropes, insulin and pancreatic enzyme supplements. The PI or delegated research team member should reassess concomitant medications at each trial visit and record the required medicines on the CRF, including at the Day 90 (+/- 7 days) visit, which may be via videolink or telephone if the participant has been discharged and is unable or does not wish to attend the research site for that follow-up visit. A photocopy of the original Concomitant Medication CRF should be sent to the LCTC within 7 days of all updates. The original copy of the CRF should only be sent to the LCTC on completion of follow-up for each participant.

# 9 Safety Reporting

Safety reporting in clinical trials aims to ensure both the safety of trial participants and the safety of current and future patients. Effective safety reporting facilitates an ongoing assessment of risk-benefit ratio. Emerging safety data allows the Sponsor to safely manage the trial by introducing amendments to the protocol, provide updated information to investigators and participants where necessary, and determine whether it is safe to continue to conduct the trial or make changes to the protocol.

## 9.1 Terms and Definitions

**“Adverse Event (AE)”**

An adverse event (AE) is any untoward medical occurrence in a subject to whom a medicinal product has been administered, including occurrences that are not necessarily caused by or related to that product.

Therefore, an AE is any unfavourable or unintended change in the function (symptoms), structure (signs) or chemistry (laboratory data) in a subject to whom an IMP has been administered, including occurrences which are not necessarily caused by or related to that product.

**“Adverse Reaction (AR)”**

An adverse reaction is any untoward and unintended response in a subject to an investigational medicinal product that is related to any dose administered to that subject.

Therefore, an AR is any unfavourable or unintended change in the function (symptoms), structure (signs) or chemistry (laboratory data) in a subject that is related to any dose of an IMP administered to that subject.

**“Unexpected Adverse Reaction (UAR)”**

An unexpected adverse reaction is an adverse reaction the nature and severity of which is not consistent with the information about the medicinal product in question set out in the Investigator's Brochure (IB) or the SPC, which may be referenced where the IMP in question is a product with a marketing authorisation.

**“Serious Adverse Event (SAE), “Serious Adverse Reaction, or Unexpected Serious Adverse Reaction”**

An AE, AR or UAR respectively that:

- results in death;
- is life threatening (places the subject, in the view of the Investigator, at immediate risk of death from the experience as it occurred – this does not include an adverse experience that, had it occurred in a more severe form, might have caused death);
- requires hospitalisation or prolongation of existing hospitalisation (hospitalisation is defined as an in-patient admission, regardless of length of stay, even if the hospitalisation is a precautionary measure for continued observation – hospitalisations for a pre-existing condition, including elective procedures that have not worsened, do not constitute an SAE);
- results in persistent or significant disability or incapacity (substantial disruption of one’s ability to conduct normal life functions);
- consists of a congenital anomaly or birth defect (in offspring of subjects, or their partners, taking the IMP regardless of time of diagnosis);
- other important medical events (these may not result in death, be life-threatening, or require hospitalisation, but may be considered a serious adverse event or experience when, based upon appropriate medical judgment, they are considered to jeopardise the subject and may require medical or surgical intervention to prevent one of the outcomes listed in this definition).

**“Suspected Serious Adverse Reaction (SSAR)”**

An adverse reaction that is classed in nature as serious and which is consistent with the information about the medicinal product in question, which in the case of a licensed product is set out in the SPC for that product, and in the case of any other investigational medicinal product is set out in the IB relating to the trial in question.

**“Suspected Unexpected Serious Adverse Reaction (SUSAR)”**

An adverse reaction that is classed in nature as serious and which is not consistent with the information about the medicinal product in question, which in the case of a licensed product is set out in the SPC for that product, and in the case of any other investigational medicinal product is set out in the IB relating to the trial in question.

**“Reference Safety Information (RSI)”**

The information used for assessing whether an adverse reaction is expected. This is contained in either the IB or the SPC.

## 9.2 Notes on Adverse Event Inclusions and Exclusions

The below sections provide guidance on what should, and should not be considered as AEs for the purposes of the RAPID-I trial:

### 9.2.1 Include

- An exacerbation of a pre-existing illness.
- An increase in frequency or intensity of a pre-existing episodic event/condition.
- A condition (even though it may have been present prior to the start of the trial) detected after trial drug administration.
- Continuous persistent disease or symptoms present at baseline that worsen following the administration of the trial treatment.
- Laboratory abnormalities that require clinical intervention or further investigation (unless they are associated with an already reported clinical event).
- Abnormalities in physiological testing or physical examination that require further investigation or clinical intervention.
- Injury or accidents.

### 9.2.2 Do Not Include

- Medical or surgical procedures* – the condition which leads to the procedure is the adverse event.
- Pre-existing disease or conditions present before treatment that do not worsen.
- Situations where an untoward medical occurrence has occurred, e.g. cosmetic elective surgery.
- Overdose of medication without symptoms or signs†.
- Widely recognised and documented clinical features of AP that are defined outcome measures in this trial (see Sections 2.4 and 7.2). These features include abdominal pain and/or nutritional deficit; pancreatic, peri-pancreatic, intra-abdominal and/or thoracic (pleural effusion) fluid collections; pancreatic and/or intra-abdominal necrosis; respiratory dysfunction/failure, cardiac dysfunction/failure and/or renal dysfunction/failure; pancreatic exocrine and/or endocrine insufficiency; infection of intra-abdominal collections, necrosis, respiratory tract, urinary tract, and/or intravascular catheters, lines and cannulas.

* *Cosmetic elective surgery is cited here as example of an event that is not reportable as an AE.*

† See also Section 8.2.4. *If overdose occurred with resulting symptoms and signs that met the protocol criteria for AE/AR/SAE/SAR then they should be reported accordingly. This bullet is to note that although overdose of medication without symptoms or signs may be excluded from AE reporting this may still require investigation to ensure the protocol and regulatory requirements are met, e.g. for IMP management and administration to ensure participant safety.*

### 9.2.3 Notification of Deaths

There is an overall likelihood of death in all cases of AP of 1-5%, the likelihood of which is proportional to severity (mild, moderate and severe, RAC)^1,2,32^. All deaths that occur during the protocol-specified AE reporting period (randomisation to Day 90 time point), regardless of relationship to study drug, must be recorded. All deaths should be reported on a “Serious Adverse Event CRF” and returned to the LCTC within 24 hours.

Death should be considered an outcome and not a distinct event. The event or condition that caused or contributed to the fatal outcome should be recorded as the single medical concept on the appropriate CRF. Generally, only one such event should be reported. The term “sudden death” should only be used for the occurrence of an abrupt and unexpected death due to presumed cardiac causes in a participant with or without pre-existing heart disease, within 1 hour of the onset of acute symptoms or, in the case of an unwitnessed death, within 24 hours after the participant was last seen alive and stable. If the cause of death is unknown and cannot be ascertained at the time of reporting, “unexplained death” should be recorded on the appropriate CRF. If the cause of death subsequently becomes available (e.g. after autopsy), “unexplained death” should be replaced by the established cause of death.

### 9.2.4 Reporting of Pregnancy / Lactation

A urine pregnancy test will be performed at Baseline for all female participants of childbearing potential. This will be repeated at the Day 14 visit. The pregnancy test will either be completed at the Day 14 visit or will be communicated to the local research team by the patient and the CRF updated accordingly. Female participants of childbearing potential must agree to use adequate contraception, as per exclusion criteria 20, for up to 6 months following administration of study treatment.

Any pregnancy reported in a female participant up to 90 days from the day of trial treatment administration, whether associated with an AE or not, must be reported to the LCTC within 24 hours of the local research team becoming aware, in accordance with the timelines and contact information for an SAE. PIs shall follow pregnancies to term to obtain the outcome of the pregnancy and this is to be reported to the LCTC. If the pregnancy outcome is determined to be anything other than a delivery without complications, if the child outcome is anything other than normal, or if the mother experiences an SAE during the pregnancy, an SAE form be completed and submitted to LCTC (please see Section 9.8.2 for further information on SAE reporting).

Patients who are known to be lactating at the time of screening will be excluded from the trial.

## 9.3 Notes on Severity / Grading of Adverse Events

The investigator responsible for the care of the participant should assign the severity/grading of each adverse event using the definitions below.

Regardless of the classification of an AE as serious or not, its severity must be assessed according to medical criteria alone using the following categories:

**Mild**: does not interfere with routine activities

**Moderate**: interferes with routine activities

**Severe**: impossible to perform routine activities

A distinction is drawn between serious and severe AEs. Severity is a measure of intensity (see above) whereas seriousness is defined using the criteria in Section 9.8.2, hence, a severe AE need not necessarily be a SAE.

## 9.4 Relationship to Trial Treatment

The local Investigator responsible for the care of the participant should assign the causality of each AE using the definitions in Table 4. An AE whose causal relationship to the study drug is assessed by the investigator as “possible”, “probable”, or “definite” is an AR.

If any doubt about the causality exists the local Investigator should inform the LCTC who will notify the CI. In the case of discrepant views on causality between the local Investigator and others, the MHRA will be informed of both points of view.

**Table 4: Definitions of Causality**

| **Relationship** | **Description** |
| --- | --- |
| **Unrelated** | There is no evidence of any causal relationship. N.B. An alternative cause for the AE should be given. |
| **Unlikely** | There is little evidence to suggest there is a causal relationship (e.g. the event did not occur within a reasonable time after administration of the trial medication). There is another reasonable explanation for the event (e.g. the participant’s clinical condition, other concomitant treatment). |
| **Possibly** | There is some evidence to suggest a causal relationship (e.g. because the event occurs within a reasonable time after administration of the trial medication). However, the influence of other factors may have contributed to the event (e.g. the participant’s clinical condition, other concomitant treatments). |
| **Probably** | There is evidence to suggest a causal relationship and the influence of other factors is unlikely. |
| **Almost certainly** | There is clear evidence to suggest a causal relationship and other possible contributing factors can be ruled out. |

## 9.5 Expectedness

The Reference Safety Information (RSI) in RAPID-I is Section 4.8 of the REMICADE^®^ 100mg powder for concentrate for solution for infusion SPC.

It is not a regulatory requirement for a reporting physician to provide their opinion of expectedness. Therefore, the reporting physician at the local research site will not be asked to make an assessment of expectedness. The assessment of expectedness will be made by the CI (or their medically qualified delegate) using the trial’s current MHRA-approved RSI (at the time of SAE onset) following receipt of the “Serious Adverse Event CRF” at LCTC.

All events graded as serious and judged by the local reporting Investigator to be possibly, probably, or almost certainly related to the IMP, and assessed as **unexpected** by the CI or their delegate, will be reported as a SUSAR (see Section 9.2 and Summary of Product Characteristics for the list of Expected Adverse Events).

## 9.6 Follow-up After Adverse Events

All adverse events (AEs/ARs/SAEs/SARs/SUSARs) should be followed until satisfactory resolution or until the local Investigator responsible for the care of the participant deems the event to be chronic or the participant to be stable.

When reporting serious adverse events (SAEs/SARs/SUSARs) the local Investigator responsible for the care of the participant should apply the following criteria to provide information relating to event outcomes:

- resolved;
- resolved with sequelae (specifying with additional narrative);
- not resolved/ongoing;
- on-going at final follow-up;
- fatal or unknown.

## 9.7 Time Period for Adverse Event Reporting

Investigators and delegated members of the local research teams will actively monitor participants for all adverse events from randomisation until the end of their trial follow-up period (i.e. Day 90 time point), and report these to the LCTC on the appropriate CRFs (see Section 9.8).

Outside of this period of active monitoring, should the Investigator (or other members of the local research team) become aware of adverse events that meet the definition of serious and related, these should also be reported to the LCTC. Any events assessed as serious, related and unexpected will be reported.

Upon becoming aware of a serious adverse event (SAE/SAR/SUSAR), the Investigator or other delegated member of the team must report this to the LCTC within 24 hours.

## 9.8 Reporting Procedures

Depending on the nature of the event, the reporting procedures below should be followed. Any questions concerning adverse event reporting should be directed to the LCTC in the first instance. A flowchart is given below to aid in determining reporting requirements.

**Figure 4. Flowchart for Reporting Requirements of Adverse Events (AE)**

**AE**

**AR**

Is the

AE related?

**Adverse Event (AE)**

**Yes**

**No**

**SAR / SUSAR**

Is the

AE serious?

Is the

AE serious?

**No**

**No**

**Yes**

**Yes**

Report to LCTC within seven days

using the AE form

CRF and submit as per routine schedule

**SAE**

Report to LCTC within 24 hours

using the SAE form

### 9.8.1 Non-serious Adverse Events (AEs/ARs)

All non-serious adverse events (i.e. AEs and ARs), whether expected or not, should be recorded on an “Adverse Event CRF”, which should be transmitted to the LCTC within seven days of the local research team becoming aware of the event.

### 9.8.2 Serious Adverse Events (SAEs/SARs/SUSARs)

All serious adverse events (i.e. SAEs, SARs and SUSARs) must be reported to LCTC within 24 hours of the local site becoming aware of the event. The “Serious Adverse Event CRF” asks for the nature of the event, date of onset, severity, corrective therapies given, outcome and causality. The reporting local Investigator should assign the causality of the event. Additional information should be sent within 5 days if the event has not resolved at the time of initial reporting.

All adverse events assessed as serious, related and unexpected (i.e. SUSARs) will be reported on to the MHRA and RAPID-I’s Research Ethics Committee (REC) by the LCTC according to the following timelines; fatal and life-threatening SUSARs within 7 days of notification to LCTC and non-life threatening SUSARs within 15 days. RAPID-I PIs will be notified of all SUSARs occurring throughout the trial by the LCTC. Local Investigators should report any SUSARs and/or SAEs as required locally.

## 9.9 Safety Reporting Responsibilities – Investigator

The PI at each centre is responsible for reporting all AEs regardless of their relationship to the trial intervention.

All serious adverse events (SAEs/SARs/SUSARs) must be reported to LCTC within 24 hours of the local research team becoming aware. All non-serious adverse events should be reported to LCTC within seven days of the local research team becoming aware.

Patient safety incidents that take place in the course of research should be reported to the National Reporting and Learning System (NRLS) by each participating NHS Trust in accordance with local reporting procedures.

**Minimum information required for reporting serious adverse events (SAEs/SARs/SUSARs)**

- Valid European Clinical Trials Database (EudraCT) number (if applicable)
- Sponsor trial number
- One identifiable coded subject (randomisation number)
- One identifiable reporter
- One SAE
- Suspect IMP (infliximab)
- A seriousness assessment
- A causality assessment

**Process for reporting serious adverse events**

i. The “Serious Adverse Event CRF” should be completed, signed and submitted to the LCTC by a local Investigator named on the trial’s ‘signature list and delegation of responsibilities log’ as responsible for reporting SAEs and making trial-related medical decisions. The Investigator should assess the SAE for the likelihood that it is a response to the trial intervention. In the absence of an authorised local Investigator, the CRF should be completed (excluding causality assessment) and signed by an alternative member of the local research team and submitted to the LCTC. As soon as possible thereafter, the responsible investigator should check the SAE form, make amendments as appropriate, provide a causality assessment, sign and re-send to the LCTC. Initial CRF reports shall be followed by detailed follow-up CRF reports as appropriate.
ii. When submitting a “Serious Adverse Event CRF” to the LCTC research sites should also telephone the appropriate Trial Manager or Data Manager on telephone number **0151 794 9774** to advise that an SAE report has been submitted.
iii. The “Serious Adverse Event CRF” must be submitted to the LCTC within 24 hours of the local research team becoming aware of the event; this must be **securely** emailed to the LCTC Central Safety Team via: **LCTCSafe@liverpool.ac.uk**
iv. The local reporting Investigator must **notify** their R&D department of the event (as per standard local governance procedures).
v. The participant must be followed-up until clinical recovery is complete and laboratory results have returned to normal, or until the event has stabilised. Follow-up should continue after completion of protocol treatment as necessary.
vi. Follow-up information should be noted on another “Serious Adverse Event CRF” by ticking the box marked ‘follow-up’ and submitting to the LCTC as information becomes available. Extra, annotated information and/or anonymised copies of test results may be provided separately.
vii. The participant **must** be identified by trial randomisation number, date of birth and initials only. The participant’s name **must not** be used on any correspondence.

### 9.9.1 Maintenance of Blinding at Sites

Systems for SUSAR and SAR reporting should, as far as possible, maintain blinding of individual clinicians and of trials staff involved in the day-to-day running of the trial. Unblinding clinicians may be unavoidable if the information is necessary for the medical management of particular patients. The safety of patients in the trial always takes priority. In each report, seriousness, causality and expectedness should be evaluated for infliximab (irrespective of dose) and placebo. Unblinding will be done through the Pharmacy if they prepare the infusion. If the infusion is prepared outside of Pharmacy, a member of the site research team will access the unblinding envelope/log labelled with the randomisation number of the patient. If unblinding needs to occur out of Pharmacy/research team hours, it should be assumed that the participant is on infliximab and treatment should be administered accordingly. All instances of unblinding must be recorded and reported to the LCTC. For further information on unblinding, please refer to Section 8.6, Blinding and Unblinding.

## 9.10 Safety Reporting Responsibilities – LCTC

The LCTC is undertaking duties delegated by the trial Sponsor and is responsible for the notification of serious adverse events to the CI (or appropriate delegate) for review and expectedness assessment, and onward expedited reporting of SUSARs and other SARs to the MHRA and REC. The LCTC will also notify trial PIs of all trial SUSARs.

Upon receipt of a “Serious Adverse Event CRF”, LCTC will liaise with the trial CI (or appropriate delegate) who will evaluate the events reported for seriousness, expectedness and causality. These will be reviewed within 24 h and those that are identified as SUSARs will be reported to MHRA and REC by the LCTC. The causality assessment given by the local Investigator at the hospital cannot be overruled and in the case of disagreement, both opinions will be provided with the report.

Timelines of onward reporting to MHRA and REC are as follows:

- SUSARs that are fatal or life-threatening must be reported not later than 7 days after the LCTC is first aware of the reaction. Any additional relevant information must be reported within a further 8 days.
- SUSARs that are not fatal or life-threatening must be reported within 15 days of the LCTC first becoming aware of the reaction.
- A list of all SARs (expected and unexpected) must be reported annually.

It is recommended that the following safety issues should also be reported in an expedited fashion:

- an increase in the rate of occurrence or a qualitative change of an expected SAR, which is judged to be clinically important;
- post-study SUSARs that occur after the patient has completed a clinical trial and are notified by a local Investigator to the LCTC.
- new events related to the conduct of the trial or the development of the IMP and likely to affect the safety of the participants, such as:

a. a SAE that could be associated with the trial procedures and which could modify the conduct of the trial;
b. a significant hazard to the participant population, such as lack of efficacy of an IMP used for the treatment of a life-threatening disease;
c. a major safety finding from a newly completed animal study (e.g. carcinogenicity);
d. any anticipated end or temporary halt of a trial for safety reasons and conducted with the same IMP in another country by the same sponsor;

- recommendations of the IDSMC, if any, where relevant for the safety of the participants.

### 9.10.1 Maintenance of Blinding at LCTC

Adverse events that are considered serious, unexpected and possibly, probably or almost certainly related to one of the trial treatments (i.e. SUSARs) will have to be unblinded at the LCTC prior to reporting to the MHRA and REC.

## 9.11 Safety Reports

Safety reports will be generated during the course of the trial that allow for monitoring of SAE and SAR reporting rates across sites and reviewed at trial oversight committee meetings. The LCTC will send Developmental Safety Update Reports (DSURs) containing a list of all SAEs to the MHRA and REC annually. Any concerns raised by the IDSMC or inconsistencies noted at a given site may prompt additional training at sites, with the potential for the LCTC to carry out site visits if there is suspicion of unreported safety events in patient case notes. Additional training will also be provided if there are unacceptable delays in safety reporting timelines. If any safety reports identify issues that have implications for the safety of trial participants, the PIs at all institutions participating in the trial will be notified.

## 9.12 Urgent Safety Measures

An urgent safety measure is a procedure not defined by the protocol, which is put in place prior to authorisation by the MHRA and REC in order to protect clinical trial participants from any immediate hazard to their health and safety.

The LCTC will notify the MHRA and REC immediately and, in any event, within 3 days that such a measure has been taken and the reasons why it has been taken. The initial notification to the MHRA will be by telephone (ideally within 24 hours) and a notice in writing will be sent within 3 days, setting out the reasons for the urgent safety measure and the plan for further action. After discussion with the MHRA and REC, further action will be agreed, which may include submission of a substantial amendment, a temporary halt, or permanent termination of the trial.

If the study is temporarily halted it may not recommence until authorised to do so by the MHRA and REC. If the study is permanently terminated before the date specified for its conclusion (in the original applications to MHRA and REC), the LCTC should notify the MHRA and REC within 15 days of the date of termination by submitting the formal End of Trial Notification.

## 9.13 Contact Details and Out-of-hours Medical Cover

Participants will be instructed to contact the local Investigator (their office number) during working hours. A patient card providing details of their participation in the RAPID-I trial will also be supplied. Outside working hours, the normal local procedure should be followed. Ward and switchboard staff should be aware where they should direct calls to both in- and out-of-hours.

# 10 Statistical Considerations

## 10.1  Introduction

Separate Statistical Analysis Plans (SAPs) will be developed prior to the interim and final analyses of the trial. The main features of these planned statistical analyses are included here in the protocol.

## 10.2  Method of Randomisation

Participants will be equally randomised to all treatment arms currently in the trial using a secure (24-hour) web-based randomisation programme controlled centrally by LCTC to ensure allocation concealment. Randomisation lists will be generated using block randomisation with random variable block length, stratified by site. The lists will be produced by an independent statistician (who is not otherwise involved in the RAPID-I trial) at LCTC.

## 10.3  Sample Size Calculation

An adaptive trial design^18^ will be adopted and treatment arms may be dropped prematurely as outlined in Section 10.4. The total planned sample size is 198, with each active treatment arm and the placebo arm having an equal number of patients (n=66). The revised sample size of 198 is based on a one-sided overall (family-wise) type I error of 2.5% and a power of 80% to detect a 25% smaller CRP AUC value than participants on placebo. This order of magnitude of drop is indicative of a meaningful impact on clinical outcomes in AP^39^. This specification of the effect of interest (reduction in CRP AUC) corresponds to a difference between the groups of 0.545s, where s is the standard deviation of CRP, and has the advantage that it is not necessary to pre-specify the standard deviation to obtain the required sample size. The revised sample size is based on the actual number of participants included in each treatment arm for the first interim analysis (not disclosed outside of the closed IDSMC meeting) and an additional interim analysis once there is sufficient data available for at least 40 participants per treatment arm as outlined below. The study will seek to recruit a total of 222 patients to account for a 10% drop-out rate in each arm but if the drop-out rate is higher this will be further inflated to ensure 80% power is still achieved. Owing to the study using stratified randomisation, it is unlikely that the number of participants in each arm will be exactly equal. The final analysis will aim to include at least 66 participants in each arm.

## 10.4  Interim Monitoring and Analyses

Two planned interim analyses will occur during the recruitment period. The first after the primary outcome has been observed for 15 participants in each arm. At this point any treatment arm with a mean CRP AUC value larger or equal to the control group will be dropped. Should both treatment arms be dropped, the study will stop.

A second interim analysis will take place after the primary outcome has been observed for a total of 40 participants in each of the remaining arms. The stopping rules will be as for the first interim analysis.

The design has been specified such that any treatment that is worse than placebo at the interim analysis has to be dropped. To account for (potentially) dropping of a dose for other reasons, the conditional error principle is to be used to adjust the design^19^.

## 10.5  Analysis Plan

The IDSMC will review the results of the interim analyses and advise the TSC if any treatments are to be dropped on the basis of the pre-defined rule. The IDSMC will also be asked to give advice on whether the accumulated data from the trial, together with results from other relevant trials, justifies continuing recruitment of further patients or further follow-up of recruited participants. Any such decision to discontinue recruitment, in all participants or in selected subgroups will be made only if the result is likely to convince a broad range of clinicians including participants in the trial and the general clinical community.

The trial will be analysed using the International Conference on Harmonisation E9(R1) Guidelines and reported using the ‘Consolidation Standard of Reporting Trials’ (CONSORT^59^) guidelines. Full analysis plans will be developed prior to the interim and final analyses of the trial. The main features of the statistical analysis are included here. The analysis of primary and secondary outcomes will use the principle of intention to treat, based on all randomised participants, as far as is practically possible.

For the final analysis of the primary outcome, comparative t-statistics will be computed using an analysis of covariance (ANCOVA) model. If the resulting test statistic for either dose falls below a critical value of -2.196, the corresponding null hypothesis of no difference between the active group and placebo can be rejected and superiority of the active dose can be claimed. Note that it is possible to claim superiority of both doses over placebo.

A last value carried forward strategy will be used to compute the CRP AUC for patients with missing CRP measurements. This strategy is conservative in that missing values lead to increased CRP AUCs and hence an increased drop-out rate will be penalized. If drop-out is similar between the arms, then the effect of this strategy cancels out in the comparison and hence leads to robust conclusions.

To assess the sensitivity of this approach we will also conduct a complete case analysis; a longitudinal model will be used as a core secondary analysis. Sub-group analyses will examine outcome in mild AP using the Harmless Acute Pancreatitis Score^3^ and in severe AP in those presenting with organ failure (SOFA scores; N.B. these tools cannot be used to stratify prior to trial treatment) as well as sub-group comparisons of the distribution of AP severities (RAC); if necessary, outcome will be analysed in a standardised period prior to cholecystectomy in mild AP.

For continuous secondary outcomes, data will be presented as means and standard deviations, medians and interquartile ranges, minima and maxima, and comparative t-statistics will be computed using an ANCOVA model. For binary secondary outcomes, data will be reported in terms of odds ratios and analysed using a logistic regression model. Secondary count data will be analysed using Poisson regression, accounting for dispersion using a negative binomial model, and/or for zero-inflation if indicated. Ordinal data will be analysed using an appropriate ordinal regression model. All regression models will be adjusted for recruiting hospital.

Bioinformatic analyses of transcriptomic, eQTL and complementary cytokine and leukocyte subset data will be undertaken and applied to differentially expressed genes, adopting an iterative approach informed by outcome, with sub-group efficacy and safety analyses of those positive or not for the differentially expressed predictive gene transcript signature.

## 10.6 Health Economic Analysis

The primary (cost utility) analysis will consider QALYs and costs from the perspective of the NHS. Unit cost data will be obtained from reference sources, including routine hospital data (NHS reference costs), the British National Formulary and nationally published data. Total costs will be calculated as the sum-product of resource use and unit cost for each patient.

The incremental analysis will be based on the mean costs and QALYs for each treatment group. The Health Economic Analysis Plan, agreed prior to the analysis, will define the analytic steps to be undertaken.

Sensitivity analyses will be conducted to test the robustness of findings. We will use such analyses based on the observed distributions of outcome and costs to test whether, and to what extent, the incremental cost-effectiveness ratios are sensitive to key assumptions in the analysis (e.g. unit prices, different utility estimates EQ-5D vs. EQ-VAS components of EQ-5D-5L).

The joint uncertainty in costs and QALYs will be addressed through application of bootstrapping and estimation of cost-effectiveness acceptability curves to allow for graphical representation of the probability of cost-effectiveness according to different thresholds of cost-effectiveness. The economic findings will be reported according to the consolidated health economic evaluation reporting standards (CHEERS) guideline^58^.

# 11 Regulatory and Ethical Approvals

## 11.1 Statement of Compliance

This trial will be carried out in accordance with LCTC Standard Operating Procedures, the World Medical Association Declaration of Helsinki (1996), the UK-legislated principles of Good Clinical Practice (GCP) and all other applicable legislation (data protection, clinical trials, human tissue).

The procedures detailed within this protocol are compliant with the Ionising Radiation (Medical Exposure) Regulations, and appropriate review by a Medical Physics Expert and Clinical Radiation Expert has been undertaken.

## 11.2 Regulatory Approval

This trial will be registered with the MHRA and a Clinical Trial Authorisation (CTA) will be obtained prior to the trial commencing.

## 11.3  Ethical Considerations

This research protocol is designed scientifically with statistical plans of sufficient power to determine in a safe manner whether infliximab is an effective treatment for AP, something that is greatly needed.

Trial participants will be recruited in a fair way, representative of all patients with AP. Patients will be randomised into one of three arms, in one of which they will receive a placebo infusion (0.9% sodium chloride) and in the other two arms the infusion plus different doses of infliximab (5 mg/kg or 10 mg/kg). Although participants will not be able to choose their own treatment, the adaptive design of the study with two interim analyses will ensure that any treatment that is worse than placebo at interim analysis will be dropped. Furthermore, participants in all arms will receive required prophylactic medication comprised of 100 mg hydrocortisone and 10 mg chlorphenamine intravenously; piperacillin/tazobactam 4.5 g intravenously t.d.s, or alternative antibiotic(s) for up to 7 days or until discharge, whichever is earlier; and adrenaline will be prescribed so as to be immediately available if necessary. There will be thorough clinical supervision with joint management from Pancreatology and Gastroenterology specialists, the latter with substantial expertise in the use of infliximab.

Recruitment of patients will be upon admission to A&E following the diagnosis of AP. Due to the nature of the study (emergency care setting and trial treatments must be initiated within 36 hours of admission) only limited time will be available to consider participation. However, in compliance with GCP, patients will be (i) accurately informed of the trial, risks, benefits, and alternatives, (ii) asked to ensure they understand how it relates to them, and (iii) make a voluntary decision about participation. They will have an opportunity to ask questions.

Moreover, as AP is an acute, life-threatening condition and the trial may produce benefit in individuals lacking capacity, such patients will be included if eligible. If patients are unable to give consent, a close relative, carer (Personal Legal Representative) or if unavailable an independent doctor or staff member (Professional Legal Representative) will be asked for a proxy decision using local standard operating procedures. The participant will be informed about the trial as soon as it is possible (once capacity has been regained) and asked to consent for continuation of any trial procedures and data collection.

Patient autonomy, privacy and welfare will be paramount and override continued pursuit of the research. Participants will be kept abreast of findings that might cause them to withdraw participation, without detriment.

As indicated in Section 2.2.1, there are risks of infliximab-related i.v. infusion reactions and an increased risk of infection from infliximab, notably exacerbation or reactivation of tuberculosis, reactivation of chronic viral infection and/or exacerbation of any bacterial infection consequent upon AP. Care has been taken to minimise these risks at the screening phase with eligibility criteria excluding patients with any history of tuberculosis, opportunistic infections or chronic viral infections, as well as through the administration of prophylactic antibiotics to all trial participants to protect against bacterial infection.

Independent ethical review and all other regulatory approvals will be sought. To minimize potential conflicts of interest, the IDMSC will ensure independent oversight, advising the TSC on continuation or not of the trial, designed to minimize bias.

## 11.4  Approvals

The protocol, PISCs and any proposed public-facing material will be submitted to an appropriate Research Ethics Committee (REC), regulatory authority (MHRA), Health Research Authority (HRA) and host institutions for written approval.

Any substantial amendments to the original approved documents will be submitted and, where necessary, approved by the above parties before use.

## 11.5 Protocol Deviation and Serious Breaches

A breach of the protocol or GCP is ‘serious’ if it meets the regulatory definition of being “likely to affect to a significant degree the safety or physical or mental integrity of the trial participants, or the scientific value of the trial”. All confirmed serious breaches of GCP or protocol will be reported to the MHRA and REC in an expedited manner by the sponsor.

If any persons involved in the conduct of the trial become aware of a potential serious breach, they must immediately report this to the LCTC who will in turn notify the Sponsor. The Sponsor will assess the breach and determine if it meets the criteria of a ‘serious’ breach of GCP or protocol and therefore requires expedited reporting to the MHRA and REC.

In determining whether or not the breach is likely to affect to a significant degree the safety, physical or mental integrity of participants, the Sponsor may seek advice from medical expert members of the Trial Management Group (TMG) and/or of the independent oversight committees (IDSMC and TSC). In determining whether or not the breach is likely to significantly affect the scientific value of the trial, the Sponsor may seek advice from the Trial Statistician. However, the Sponsor retains responsibility for the assessment of whether or not a breach meets the definition of ‘serious’ and is subject to expedited reporting to MHRA and REC.

Breaches confirmed as ‘serious’ will be reported to the MHRA and REC within 7 days by the sponsor and notified to the TMG, IDSMC and TSC at their next meetings.

Any requests for additional information from the Sponsor, TMG, TSC, IDSMC, REC or MHRA will be promptly actioned by the relevant member(s) of the research team and open communication will be maintained to ensure appropriate corrective actions are taken and documented.

Other incidences of protocol non-compliance will be recorded as protocol deviations, the frequency of which will be monitored and reported to the trial oversight committees.

# 12 Data Management and Trial Monitoring

Details of the monitoring to be carried out for the RAPID-I study are included in the RAPID-I Trial Monitoring Plan, maintained separately to this protocol.

Trial Oversight Committees related to the monitoring of the trial are detailed in Section 14.4.

## 12.1 Source Documents

In order to resolve possible discrepancies between information appearing in the CRF and any other patient related documents, it is important to know what constitutes the source document and therefore the source data for all information in the CRF.

Source data is defined as: “All information in original records and certified copies of original records of clinical findings, observations, or other activities in a clinical trial necessary for the reconstruction and evaluation of the trial. Source data are contained in source documents (original records or certified copies).” (ICH E6, 1.51).

Source documents are defined as: “Original documents, data, paper and electronic records (e.g. hospital records, clinical and office charts, laboratory notes, memoranda, participant diaries or evaluation checklists, pharmacy dispensing records, recorded data from automated instruments, copies or transcriptions certified after verification as being accurate copies, microfiches, photographic negatives, microfilm or magnetic media, x-rays, participant files, and records kept at the pharmacy, at the laboratories and at medico-technical departments involved in the clinical trial).” (ICH E6, 1.52).

The CRF will be considered the source document for data where no prior record exists and which is recorded directly in the CRF. A RAPID-I source document list will be produced for each site.

Date(s) of conducting the informed consent process including date of provision of patient information, confirmation of full eligibility, randomisation number and the fact that the patient is participating in a clinical trial (including possible treatment arms) should be added to the patient’s medical record chronologically.

## 12.2 Data Capture Methods

The trial paper CRFs are the primary data collection instrument. All data requested on the CRF must be recorded. All missing data must be explained as follows:

- if a space on the CRF is left blank because the procedure was not done or the question was not asked, “N/D” should be recorded on the CRF;
- if the item is not applicable to the individual case, “N/A” should be recorded;
- if the data item is un-known, “NK” should be recorded;
- if a data item has not been recorded on source data, ‘NR’ should be recorded.

All changes to original CRF entries (e.g. correction of errors) must be initialled and dated in accordance with GCP.

Participant questionnaires such as EQ-5D-5L, Health Resource Use and pain numerical rating scales are source documents and **sites should photocopy them** in order to retain a copy at site before mailing originals to LCTC. Local research teams will ensure CRFs and questionnaires are sent to LCTC separately to the consent form as the consent form has patient identifiable data on it.

The LCTC will distribute CRFs to sites on an on-going basis. Completed CRFs should be returned to LCTC within 2-3 weeks of the visit date.

## 12.3 Monitoring

### 12.3.1 Central Monitoring

Data received at LCTC will be checked for missing or unusual values (range checks) and checked for consistency over time. Any suspect data will be returned to the site in the form of data queries. Data query forms will be produced at the LCTC from the trial database and sent either electronically or through the post to a named individual (as listed on the site delegation log). Sites will respond to the queries providing an explanation/resolution to the discrepancies and return the data query forms to LCTC. The forms will then be filed along with the appropriate CRFs and the appropriate corrections made on the database. There are a number of monitoring features in place at the LCTC to ensure reliability and validity of the trial data, to be detailed in the Trial Monitoring Plan.

Central checks of consent will be completed for each participant to ensure the completeness of consent and that the timing of consent is in line with the protocol.

Central monitoring will be performed in a proportionate manner as informed by the Trial Risk Assessment and Monitoring Plans.

12.3.2 Clinical Site Monitoring

In order to perform their role effectively, the Trial Manager (or Monitor) and persons involved in Quality Assurance and Inspection may need direct access to primary data, e.g. patient records, laboratory reports, appointment books, etc. Since this affects the patient’s confidentiality, this fact is included on the Participant Information Sheet and Informed Consent form (PISC).

## 12.4 Confidentiality

Individual participant medical information obtained as a result of this study is considered confidential and disclosure to third parties is prohibited with the exceptions noted below. All persons involved in the trial have a duty to preserve the confidentiality of participants taking part in the study. The University of Liverpool is registered as a Data Controller with the Information Commissioners Office and will act as a Joint Data Controller in this trial, with Bangor University (Health Economics Analysis, Section 7.4.1)

Verification that appropriate informed consent has been obtained will be confirmed by the supply of copies of participants’ signed informed consent forms to the LCTC by recruiting centres, therefore participants’ names data will be transferred to the LCTC. This requirement will be explicitly stated in the PISC.

CRFs will be pseudonymised, will not contain details of participant names and will be labelled with the unique trial randomisation number. Medical information may be given to the participant’s medical team and all appropriate medical personnel responsible for the participant’s welfare. Patient CECT scans taken on Day 14 +/- 7 days will be pseudonymised (containing limited personal identifiable information, such as randomisation number and date of scan, but not the patient’s name etc.) and provided to a central panel for review.

The LCTC will be undertaking activities requiring the transfer of identifiable data. The transfer of these data is disclosed in the PISC.

## 12.5 Quality Assurance (QA) and Quality Control (QC)

QA includes all the planned and systematic actions established to ensure the trial is performed and data generated, documented, recorded and reported in compliance with applicable regulatory, ethical and governance requirements. QC includes the operational techniques and activities performed within the QA system to verify that the requirements for quality of the trial-related activities are fulfilled: e.g. state what clinical site monitoring (and audit) is planned, if any. In accordance with the monitoring plan, site visits will or will not be conducted and source verification performed, if central monitoring processes indicate these are required. Monitoring activities could include, but are not limited to the following:

- The Trial Manager at LCTC will verify that appropriate approvals are in place prior to initiation of a site and the relevant personnel have attended trial specific training.
- Greenlight checklists will be completed to confirm that all approvals are in place prior to trial initiation at LCTC and the individual site.
- The TMG will determine the minimum key staff required to be recorded on the site delegation log in order for the site to be initiated.
- Data will be evaluated for compliance with the protocol and for accuracy in relation to source documents.
- Types and mechanisms of training of staff for the study will be specified.
- The PI and other key staff from each centre will attend site initiation training, coordinated by the LCTC, which will incorporate elements of trial-specific training necessary to fulfil the requirements of the protocol.
- The TMG will monitor screening, randomisation and consent rates between centres.
- The process for consent, recruitment and randomisation will be evaluated for compliance with the protocol.
- Data quality checks and monitoring procedures will be undertaken in line with the trial Data Management Plan, maintained separately to this protocol.
- The IDSMC and independent members of the TSC will provide independent oversight of the trial.

## 12.6 Records Retention

All trial documents will be retained for a maximum period of 25 years from the End of Trial.

The PI is responsible for archiving of all relevant source documents so that the trial data can be compared against source data after completion of the trial (e.g. in case of inspection from authorities).

The PI at each investigational site must make arrangements to store the essential trial documents, (as defined in Essential Documents for the Conduct of a Clinical Trial, ICH E6, Guideline for GCP) including any trial site files provided (e.g. Investigator Site File, Pharmacy Site File, IMP Preparation File) until the LCTC informs him/her that the documents are no longer to be retained. The PI is required to ensure the continued storage of the documents, even if the investigator, for example, leaves the clinic/practice or retires before the end of the required storage period. Delegation must be documented in writing.

The LCTC undertakes to store the sections of the Trial Master File relevant to their delegated duties, and originally completed CRFs, except for source documents pertaining to the individual investigational site, which are kept by the investigator only. All electronic CRFs and trial data will be archived onto appropriate media for long-term accessible storage. Hard copies of data will be boxed and transferred to secure premises where unique reference numbers are applied to enable confidentiality, tracking and retrieval.

All other persons and organisations involved in the trial will be responsible for storing and archiving the parts of the TMF relevant to their delegated duties (e.g. laboratories, IMP distributors, etc.).

# 13 Indemnity

RAPID-I is sponsored by the University of Liverpool and co-ordinated by the LCTC. The University of Liverpool has vicarious liability for the actions of its staff, when through the course of their employment they are involved in the design and initiation of clinical research, including but not limited to the authorship of the Protocol. The University of Liverpool has appropriate insurance in place to cover this liability.

As this is an investigator-initiated study, The Association of the British Pharmaceutical Industry (ABPI) guidelines for patient compensation by the pharmaceutical industry do not apply. However, in terms of liability, NHS Trust and Non-Trust Hospitals have a duty of care to patients treated, whether or not the patient is taking part in a clinical trial, and they are legally liable for the negligent acts and omission of their employees. Compensation is therefore available in the event of clinical negligence being proven.

**Clinical negligence is defined as:**

*“A breach of duty of care by members of the health care professions employed by NHS bodies or by others consequent on decisions or judgments made by members of those professions acting in their professional capacity in the course of their employment, and which are admitted as negligent by the employer or are determined as such through the legal process”.*

# 14 Roles and Responsibilities

## 14.1 Role of Study Sponsor and Study Funder

The Sponsor of this trial is the University of Liverpool. The Sponsor will ensure that clear agreements are reached, documented and carried out, respecting the dignity, rights, safety and wellbeing of participants and the relationship with healthcare professionals. This will provide for proper design, management, initiation, conduct, monitoring, data collection, data analysis, data protection, financing and reporting of this trial, meeting appropriate scientific, legal and regulatory standards. The responsibility for design, conduct, management, data analysis, data interpretation, manuscript writing, and dissemination of results is delegated to the Trial Management Group.

The funders of this study are the Medical Research Council and National Institute for Health and Care Research who are providing financial funding through the Efficacy and Mechanism Evaluation Programme. The funders will ensure there is proper use of the funds they have allocated to provide value for money. The funders assure the quality of the study, taking the lead in establishing that the research proposal is worthwhile, of high scientific quality, has an appropriate research infrastructure with expert clinical trial management, has the capacity to comply with the principles of GCP and represents good value for money.

## 14.2 Funding and Support in Kind

| **Funder(s)** | **Financial and Non-financial Support Given** |
| --- | --- |
| Medical Research Council and National Institute for Health and Care Research | Financial funding (£1,792,074) |
| Liverpool University Hospitals NHS Foundation Trust | Financial funding (£119,926) |
| MSD (MERCK SHARP & DOHME LIMITED, Hertford Road, Hoddesdon, Hertfordshire, EN11 9BU) | MSD supplied the IMP infliximab (REMICADE^®^) administered as trial medication in this study (MSD MISP Database number 53626) through an Investigator Initiated Clinical Trial Research Agreement between MSD and the Liverpool University Hospitals NHS Foundation Trust (LUHFT) to 30^th^ September 2024. MSD did not provide any financial funding in connection with this agreement. |

## 14.3 Protocol Contributors

The individuals who contributed substantively to protocol development and drafting are listed below:

| **Name** | **Affiliations** | **Contribution to protocol** |
| --- | --- | --- |
| Robert Sutton | Institute of Systems, Molecular and Integrative Biology, University of Liverpool | Led the writing of this protocol, clinical and scientific arrangements, trial design and conduct |
| Sreedhar Subramanian | Cambridge University Hospitals NHS Foundation Trust | Clinical arrangements, trial design and conduct |
| Richard Body | University of Manchester | Clinical arrangements, trial design and conduct |
| Thomas Jaki | MRC Biostatistics Unit, Cambridge | Statistical arrangements, trial design and conduct |
| Catherine Spowart | LCTC, University of Liverpool | Protocol development, governance arrangements and trial conduct |
| Matt Smyth | LCTC, University of Liverpool | Protocol development, governance arrangements and trial conduct |
| Michaela Brown | LCTC, University of Liverpool | Statistical arrangements, trial design and conduct |
| Carrol Gamble | LCTC, University of Liverpool | Statistical arrangements, trial design and conduct |
| Dyfrig Hughes | Centre for Health Economics and Medicines Evaluation, Bangor University | Health Economics aspects |
| Catrin Plumpton | Centre for Health Economics and Medicines Evaluation, Bangor University | Health Economics aspects |
| Diane Latawiec | Institute of Systems, Molecular and Integrative Biology, University of Liverpool | Laboratory arrangements. |
| Amy Lucas | TMG PPI Representative | Protocol development and trial conduct |

Advice was received from Co-Applicants to the EME Funding Programme, External Experts and the BRU (subsequently LUHFT Pancreas) Patient Advisory Group.

The design, conduct, data analysis, data interpretation, manuscript writing, and dissemination of results are the responsibility of the TMG, subject to the approval of the University of Liverpool as Sponsor and the Medical Research Council and National Institute for Health and Care Research as Funders.

## 14.4 Trial Committees

### 14.4.1 Trial Management Group (TMG)

A TMG will be formed comprising the CI, other lead investigators (clinical and non-clinical), members of the LCTC and a lay member. A Sponsor representative will be invited to attend TMG meetings. The TMG will be responsible for the day-to-day running and management of the trial. Refer to the TMG terms of reference and trial oversight committee membership document for further details. Meetings will be in Liverpool with video- and/or teleconferencing. The TMG will report to the TSC.

### 14.4.2 Trial Steering Committee (TSC)

The TSC will consist of an independent medical expert chairperson, an independent biostatistician and two independent lay members. TMG representatives will be given the opportunity to contribute as non-independent and non-voting TSC members. The role of the TSC is to provide overall supervision for the trial and provide advice through its independent Chairman. The ultimate decision for the continuation of the trial lies with the TSC.

The TSC will first convene prior to the start of recruitment and will then define frequency of subsequent meetings (at least annually). Refer to the TSC terms of reference and trial oversight committee membership document for further details.

### 14.4.3 Independent Data and Safety Monitoring Committee (IDSMC)

The IDSMC consists of an independent clinical trialist chairperson, an independent expert in Pancreatology and an independent expert in medical statistics.

The IDSMC will be responsible for reviewing and assessing recruitment, interim monitoring of safety and effectiveness, trial conduct and external data. The IDSMC will first convene prior to the start of recruitment and will then define frequency of subsequent meetings (at least annually). Details of the interim analyses and monitoring are provided in Section 10.4.

The IDSMC will provide a recommendation to the TSC concerning the continuation of the study. Refer to the IDSMC charter and trial oversight committee membership document for further details.

# 15 Publication and Dissemination

## 15.1  Publication Policy

The results from different centres will be analysed together and published as soon as possible. The design, conduct, data analysis, data interpretation, manuscript writing, and dissemination of results are the responsibility of the TMG on behalf of the University of Liverpool as Sponsor, the Medical Research Council and National Institute for Health and Care Research as Funders.

The TMG will form the basis of the Writing Committee and advise on the nature of publications. The Uniform Requirements for Manuscripts Submitted to Biomedical Journals (<http://www.icmje.org/>) will be respected. The National Clinical Trial (NCT) identifier allocated to this trial will be attached to any publications resulting from this trial.

The members of the TSC and IDSMC will be listed with their affiliations in the Acknowledgements / Appendix of the main publication.

### 15.1.1 Authorship

Contributors to all four of (i) the design, conduct, data analysis and interpretation, (ii) writing, (iii) manuscript approval and (iv) accountability for the integrity of the work will, depending on their contribution and journal requirements, be included by name at the manuscript head or listed at the end in a byline as members of the Great Britain and Ireland Pancreatitis Study Group. It is expected that those contributors who fulfil all four of the above criteria and are listed in the byline will be identified in MEDLINE as authors, in accordance with the policy of the International Committee of Medical Journal Editors.

## 15.2  Dissemination to Key Stakeholders

This trial will be promoted by the CI, PIs and contributing parties in national and international Pancreatic Societies including the Pancreatic Society of Great Britain and Ireland, European Pancreatic Club, American Pancreatic Association and International Association of Pancreatology, with encouragement of presentations, lectures and symposia. The LUHFT Pancreas Patient Advisory Group will promote the study nationally with a long-term aim of international networking. Communication via the Clinical Research Networks, major NHS Trusts and pancreatic and gastrointestinal services will highlight the work, taking opportunities to discuss via various media.

Once findings concerning efficacy, safety, immune mechanisms in AP, validity of the predictive differential gene expression signature, development of a treatment response signature, a Cumulative AP Outcome Score and quality indicators have been made, the organisations outlined above as well as others will be used by those contributing to the study, including patients and the public, to present the findings and to carry forward work arising from the implications of the findings.

Publication of all original work and associated reviews is likely to lead to one or more high impact publications and potential future work. These findings may lead to several shifts in approach, including further AP trials as well as standard clinical management of the diagnosis and treatment in AP. If RAPID-I shows infliximab to be an effective treatment for AP, a pivotal phase III trial (RAPID-II) will be pursued. Furthermore, should the transcriptomic signatures be validated, commercialisation will be encouraged, to lead to market application.

The results of RAPID-I will be disseminated regardless of the magnitude or direction of effect.

## 15.3  Data Sharing

Individual clinicians must undertake not to submit any part of their individual data for publication without the prior consent of the TMG. All publications shall include a list of participating PIs and collaborators.

# 16 Chronology of Protocol Amendments

16.1 Version 1.0 (31/08/2017)

Please note version 1.0 (31/08/17) was not submitted to HRA, Ethics or MHRA for approval.

16.2 Version 2.0 (20/04/2018)

Please note version 2.0 (20/04/2018) was submitted to HRA and Ethics for approval.

16.3 Version 3.0 (28/06/2018)

After receiving ethical provisional opinion, the following amendments were incorporated prior to resubmission. Version 3.0 was also submitted to MHRA for approval.

- Exclusion criterion added; patients with a body weight of over 200 kilograms (Section 5.2, Page 24)
- Randomisation issues contact number updated (Section 6.5, Page 31 and Section 9.9, Page 53).
- New treatment instructions, as per SPC update (30/05/2018); all patients, regardless of treatment arm, weighing over 100kg must receive 500 ml trial infusion rather than 250 ml, to ensure concentration of infliximab does not exceed 4 mg/ml (Section 8.2.2, Page 49, Section 8.4, Page 50 and Section 8.8, Page 52).
- AUDIT questionnaire removed from appendix.

16.4 Version 4.0 (03/08/2018)

Amendments were required to the protocol version 3.0 (28/06/2018) following receipt of a grounds for non-acceptance letter (with right to amend request) from the MHRA. In parallel to the MHRA requested amendments, further amendments not requested by the MHRA were also added (although agreed by the MHRA). The amendments are outlined below:

- Rationale for 10mg/kg dose update; the rationale to support the proposed higher dose of 10mg/kg was expanded (Section 2.2, Pages 16 and 17).
- Additional secondary outcome; pancreatic sufficiency (faecal elastase on Day 28 (+/- 2 days) and Day 90 (+/- 3 days); HbA1c on Day 90 (+/- 3 days)) included as an additional secondary outcome. Protocol summary on page 13 updated to reflect this change also (Section 2.4.2, Page 21).
- Exclusion criterion update; patients with suspected infections at the time of AP presentation now excluded also. Previously only patients with known established infections prior to AP onset were to be excluded. The protocol summary on page 13 also updated to reflect this (Section 5.2, Page 24).
- Exclusion criterion update; women of childbearing potential to be excluded who do not agree to use adequate contraception up to 6 months after the infliximab infusion, as per SPC Section 5.2, Page 25). The protocol summary on page 13 also updated to reflect this.
- Schedule of Assessments; table updated to include triglyceride standard care blood test at screening and as required from trial entry. Faecal elastase assessment included at Day 28 and Day 90. HbA1c included at Day 90 (Section 7.1, Pages 33 and 34).
- Continuous recordings; faecal elastase and glycated haemoglobin (HbA1c) included in time point descriptions (Section 7.1.1, Pages 36 and 37).
- Reporting of Pregnancy / Lactation update; updated to align with the exclusion criterion update above (Section 9.2.4, Page 48).
- Reference Safety Information update; Section 4.8 ‘Undesirable effects’ of the SPC is now defined as the RSI for the study, sections 4.3-4.9 (Section 9.5, Page 49).

16.5 Version 5.0 (18/10/2018)

Amendments were required to Version 4.0 (03/08/2018) following confirmation from the MHRA that clarification was required in regard to monitoring of patient liver function test results. The below amendments were made in addition to the aforementioned amendment:

- CTA and NCT reference numbers added (Title Page, Page 1).
- Monitoring of liver function tests; clarification that LCTC will not be centrally monitoring patient liver function test results, this will be the responsibility of the recruiting site caring for each patient, as per standard care. Only albumin will be requested by LCTC (Section 2.2.1, Page 20 and throughout Section 7, Page 32-39).
- Calculation of total volume of infusion administered; clarification of whom will calculate total volume administered to patients, this will be completed by LCTC, not sites (Section 8.8, Page 44).
- Concomitant medication to be recorded; insulin and pancreatic enzyme supplements added to the listed of concomitant medications that should be recorded on the concomitant medication form (Section 8.9.3, Page 45).
- Completed CRF return timeline; completed CRFs to be returned to LCTC within 2-3 weeks, protocol previously stated 2 weeks only (Section 12.2, Page 60).
- Protocol contributors; Sean Gaines added as a protocol contributor (protocol development, governance arrangements and trial conduct) (Section 14.3, Page 66).

16.6 Version 6.0 (28/11/2018)

Amendments were required to Version 5.0 (18/10/2018) following confirmation that not all recruiting sites will have access to bed weighing scales in their Accident and Emergency Department. The below amendment was made to clarify that any type of weighing scale is permitted for determination of patient weight:

- The weight of the consented patient will be determined through the use of a weighing scale, used to calculate trial treatment dosage. Any type of hospital weighing scale is appropriate for this purpose, depending on site availability and mobility of the patient. (Section 6.4, Baseline, Page 29)

16.7 Version 7.0 (09/07/2019)

Amendments were required to Version 6.0 (28/11/2018) following feedback from a number of potential participating sites confirming they would be unable to participate in the trial due to a number of reasons. Trial infusions are no longer required to be completed by a Trials Pharmacy department as previously specified (although this remains as an option for sites should they wish), alternatively, sites may identify unblinded, independent research nurses, who may prepare and blind the infusion, aseptically, within an area that is separate from the blinded research team and the patient - the protocol has been amended throughout to reflect this. A number of minor administrative changes have also been included in this updated version. A summary of the changes can be found below:

- Updated postal address of the LCTC and removal of obsolete FAX number. An email address has been included for Bangor University (Page 6).
- Main inclusion and exclusion criteria within the protocol summary were updated to align with the changes made to Section 5, Study Population, please see below for further details (Section 1, Page 14-15)
- Number of recruiting sites increased up to 40 (Section 1, Page 15)
- Addition of a second type of participating centre, Data Collection only centres. Eligible secondary care sites may now also contribute to patient recruitment. The criteria for each site type have been separated into different sub-sections (Section 4.1, Pages 27-28).
- Inclusion criterion 2 has been amended to state that the trial infusion must be commenced within 36 hours of the onset of abdominal pain; it no longer includes the requirement of the trial infusion to be commenced within 12 hours of recorded hospital admission (Section 5.1, Page 30).
- Exclusion criterion 3 has been amended to exclude patients for which no time can be identified for the onset of their abdominal pain (Section 5.2, Page 30)
- Patient transfers; further text has been included to instruct research teams at secondary care centres to liaise with research teams at their tertiary centre, prior to referring the trial patient for specialist care, ensuring they are able to continue trial follow-up assessments and procedures (Section 7.5, Page 44).
- Reporting of pregnancy and/or lactation; further text included instructing sites to report incidents as SAEs i.e. delivery with complication, abnormal birth and SAEs experienced by the mother during the pregnancy (Section 9.2.4, Page 56).
- Blinding and unblinding; unblinding pressure-sealed envelopes will be provided to sites where the infusion is prepared outside of Pharmacy – unblinding instructions have been included for such sites (Section 8.6, Pages 50-51).
- Removal of LCTC FAX number in safety reporting instructions; sites are instructed to securely email Serious Adverse Event CRFs to the trial email account (Section 9.9, Page 61).
- Confidentiality; Bangor University noted as a Joint Data Controller in RAPID-I (Section 12.4, Page 70).
- Patient CECT scans (Day 14 +/- 2 days) will be pseudonymised and provided to a central panel for review (Section 12.4, Page 70).
- Addition of Richard Body and Quentin Nunes as protocol contributors (Section 14.3, Page 75).

16.8 Version 8.0 (02/04/2020)

Amendments were required to Version 7.0 (09/07/2019), in response to the COVID-19 pandemic, to allow local research teams to collect/complete the following data/assessments remotely for outpatients who were unable to return to the research site for their scheduled follow-up visits (prior approval from LCTC must be given for each individual patient for whom this method of follow up is proposed:

- Adverse events
- Concomitant medications
- EQ-5D-5L questionnaires
- HRUQ
- Results of Day 28 pregnancy test for female patients of childbearing potential (posting of urine pregnancy test to patients’ residence)

16.9 Version 9.0 (12/08/2020)

The following amendments were made to Version 8.0 (02/04/2020):

- Two exclusion criteria have been added as follows to ensure patients at high risk from COVID-19 infection are not entered into the trial, as the effects of infliximab on COVID-19 infection in high risk patients are not known and could be deleterious:
  - Known severe respiratory conditions including cystic fibrosis, severe asthma and severe chronic obstructive pulmonary disease (COPD), on home oxygen or home mechanical ventilation.
  - Known rare diseases or inborn errors of metabolism that significantly increase the risk of infections, including severe combined immunodeficiency (SCID) and homozygous sickle cell disease.

The exclusion criterion that lists ‘known established infection prior to or suspected infection at the time of onset of AP’ now incorporates ‘including COVID-19’ as follows:

- Known established infection prior to or suspected infection, including COVID-19‡, at the time of AP onset.
- The Day 4 time point window has been extended to +/- 1 day (48 – 120 h) from the start of trial infusion, to ensure that this time point will not have to be completed during a weekend for patients who are recruited on a Wednesday or Thursday.
- The time point for CECT scanning remains at +/- 2 days but has been widened to +/- 7 days if there is clinical indication for CECT scanning during this latter wider time interval, to prevent unnecessary duplication of CECT scanning.
- The Day 90 time point window has been extended to +/- 7 days to allow patients flexibility in returning to the participating centre, as many are likely to have been discharged a number of weeks before this time point.
- Attention is drawn to the requirement for research teams to follow all relevant precautions to prevent spread of SARS-CoV-2 and COVID-19 infection, according to all national and local guidelines in force, including required use of personal preventive equipment (PPE), social distancing and local policies on testing.
- The requirement for an independent literate witness has been added for patients and/or legal representatives unable to read.
- All references to the previous ‘CTRC, Clinical Trials Research Centre’ have been replaced with ‘LCTC, Liverpool Clinical Trials Centre’. The Trial Unit logo on the front page has also been updated.
- The address of the CI (Institute of Systems, Molecular and Integrative Biology) and hospital Trust (Liverpool University Hospitals NHS Foundation Trust in place of the Royal Liverpool and Broadgreen University Hospitals NHS Trust) have been updated.
- Four references have been added to support the approach to COVID-19 and CECT scanning.

## 16.10 Version 10.0 (30/11/2021)

The following amendments were made to Version 9.0 (20/08/2020):

- The Sponsor signatory and address has been changed from Mr Alex Astor of the Research Support Office to Miss Karen Wilding of the Clinical Directorate at the University of Liverpool.
- The explanation for email approval for the protocol via email has been removed.
- The length of time that patients report typical continuous upper abdominal pain as one of three features to establish the diagnosis of acute pancreatitis as an inclusion criterion has been widened to two days prior to admission. This is to increase the potential recruitment of suitable patients, as some patients cannot remember the precise hour of onset of their abdominal pain.
- Two days prior to admission has been defined as from midnight at the beginning of two days prior to the day of admission; days rather than hours has been used to allow for the difficulties in recall of the exact time of onset.
- As a corollary, the maximum length of time from admission to the start of trial treatment has been specified at 24 hours, allowing 120 min within the 24 hour period for the preparation of trial medication. This is to ensure that the trial treatment is started promptly after admission, but to allow sufficient time for this to be undertaken.
- Day 7 (+/- 1 day) and Day 28 (+/- 2 days) trial blood testing and questionnaire administration have been removed to reduce the requirements for patient reattendance for trial assessments, as these have impeded adoption and conduct of the trial by many centres, impose additional burdens on patients, and are particularly difficult during the reduced outpatient capacity resulting from the SARS-CoV-2 pandemic. Infliximab levels are now measured on Day 4 (+/- 1 day, as before) and Day 14 (+/- 2 days, instead of Day 28) and infliximab antibody assay is now undertaken on Day 14 (+/- 2 days, instead of Day 28). Pregnancy testing and the HRUQ are now scheduled for Day 14 (+/- 2 days) instead of Day 28. AUC on Days 2, 4 and 14 is a robust measure of the primary outcome CRP, for which the sample size, power and interim analyses remain the same. It is anticipated that effect of this amendment on the AUC for the primary outcome will be modest, as there is a linear relationship between CRP on Days 4, 7 and 14, by when most of any treatment effect is anticipated, while the contribution of Day 28 CRP is insufficient to justify its inclusion.
- Secondary outcome recording of cumulative pain scores, opiate requirements, nutritional deficit and SOFA scores has been amended to continue until discharge (as before) or Day 14 for patients remaining in hospital with acute pancreatitis. Measurement of serum albumin, haematocrit and neutrophils on Day 28 (+/- 2 days) has been removed in line with the change to the measurement of CRP. Measurement up to Day 14 (+/- 2 days) allows for representative capture of the effect that infliximab may have on these parameters in acute pancreatitis.
- Systemic inflammatory response syndrome has been removed as a secondary outcome measure as this is used to predict severity not to determine the outcome of acute pancreatitis.
- The time point for CECT scanning has been widened to +/- 7 days as some patients require CECT scanning during this latter wider time interval, and early repetition of CECT scanning usually provides little extra information and is undesirable.
- Faecal elastase and HbA1_C_ measurements have been removed as these are imprecise and do not justify the additional collection burdens on trial patients and research staff.
- The follow up visit on Day 90 (+/- 7 days) may now be undertaken remotely by videolink or telephone if patients are unable or would prefer not to attend the research site in person, and no approval for this is now necessary from LCTC. No blood test results will now be recorded from this time point as it will make it easier for the trial to be completed, and the amount of information gained from such results will be limited and have little bearing on the trial outcome.
- Addition of information regarding SARS-CoV-2 vaccination following participation in RAPID-I.
- Addition of text to state that should a participant not attend Day 14, a pregnancy text will be posted out for completion.
- The number of patients with sufficiently complete follow up data to carry out the first interim analysis has been reduced to 20 in each group to reach an earlier conclusion about continuation of the trial, an adjustment that has had minimal effect on the power of the trial (reduced to 89.1% from 90%) and the likelihood of type 1 error (reduced from 0.025 to 0.024) but reduces the time taken to recruit sufficient participants and therefore reduces costs to achieve this milestone.
- Local research teams are instructed to securely email safety report forms to the LCTC Central Safety Team via [LCTCSafe@liverpool.ac.uk](mailto:LCTCSafe@liverpool.ac.uk), rather than the RAPID-I general inbox.
- Unblinding process for sites where IMP prepared by unblinded research team amended to mirror the process where IMP is prepared by pharmacy.
- Suggestions concerning the design from External Experts have been acknowledged, notably the utility of pulse oximetry in place of arterial oxygen measurement, as the trial design has been presented and shared internationally.
- Two references (48 and 49) have been added that suggest infliximab has beneficial effects on patients who develop COVID-19 infection in the presence of other inflammatory conditions.
- Three references (50-52) have been added that provide guidance on vaccination to prevent COVID-19 infection, drawing attention to standard of care for such vaccination. The current standard advice is for patients to delay first or second vaccination until four weeks or more after discharge from hospital to allow for recovery from acute pancreatitis and promote vaccination efficacy. The third reference confirms that by then the pharmacokinetics of infliximab are such that the trial treatment will have no effect on the response to vaccination.

## 16.11 Version 11.0 (18/03/2022)

The following amendments were made to Version 10.0 (30/11/2021):

- Correction in the Protocol Summary and in section 2.2.1 Benefits and Risks of the length of time permitted from hospital admission to start of the trial infusion to accord with the change in timings made in Version 10.0.

## 16.12 Version 12.0 (18/07/2023)

The following amendments were made to Version 11.0 (18/03/2022):

- Dr Sreedhar Subramanian’s address has been updated from Liverpool University Hospitals NHS Foundation Trust to Cambridge University Hospitals NHS Foundation Trust, as he has moved employment.
- The name of the NIHR has been updated from National Institute for Health Research to National Institute for Health and Care Research, now named.
- NIHR National Biosample Centre has been replaced by UK Biocentre, now named.
- The eligibility restriction of two days prior to the day of admission allowed for the onset of abdominal pain characteristic of acute pancreatitis has been removed. This restriction has previously excluded patients who have reported such pain for longer periods, despite having been found to have normal amylase levels during the earlier stages of their pain, when the pain would be more likely from another cause e.g. biliary colic prior to the development of acute pancreatitis. The removal of this restriction aligns the trial more to real-world clinical practice.
- The length of time during which trial medication can be started has been increased from 24 hours to 36 hours from admission to allow participation of patients for whom trial medication cannot otherwise be prepared within office hours. Some patients are admitted during daylight and/or diagnosed with AP some hours after admission, by which time it may be too late for Pharmacies to prepare trial medication on the day of admission; were trial medication to be prepared the following day, 24 hours would have already elapsed by the time it might be possible to start trial medication.
- Previous AP is no longer an exclusion criterion, provided there is a new diagnosis or a new attack of AP, to ensure that the trial is more representative of real-world clinical practice, as recommended by the IDSMC. Previous AP diagnosed within the last 30 days remains an exclusion criterion, so that patients readmitted with continuation of a previous attack are not offered participation.
- Clarification regarding adequate contraception in section 5.2 which now states that adequate contraception includes prior surgical sterilisation; birth control pill, patch or injection; intrauterine device; male or female condom (but not both); female cap with spermicide or total sexual abstinence; but not periodic abstinence, withdrawal or spermicides only.
- Information on the CATALYST trial that tested and found infliximab to be safe in COVID-19 pneumonia has been added to section 2.2.1 Benefits and Risks.
- Advice on COVID-19 vaccination in standard of care after AP has been added to section 2.2.1, to include vaccination after a first or second dose.
- The necessity for fasting to measure serum triglycerides has been removed from Table 3 Schedule of Assessments, as fasting is no longer considered optimal for this.
- Local CRP results may be requested if CRP for central analysis is unobtainable.
- The method of assessment of the CECT undertaken on Day 14 (+/- 7 days) is now specified in section 7.2 Efficacy as the modified CT severity index, which provides a score of up to 10 for inflammation, fluid collections, necrosis and extra-pancreatic complications.
- Sub-section 9.2.2 Do Not Include (in section 9.2 Notes on Adverse Event Inclusions and Exclusions) now specifies widely recognised and documented features of AP that should not be considered as AEs for the purposes of the RAPID-I trial. This is in response to previous reporting as AEs of typical complications of AP that are defined outcome measures in this trial.
- In the light of more comprehensive analyses of death rates from AP, the overall likelihood of death from AP in section 9.2.3 Notification of Deaths has been modified from 3-5% to 1-5%.
- Matt Smyth, the present Trial Manager, and Carrol Gamble, Professor of Medical Statistics, have been added to section 14.3 Protocol Contributors, as they have contributed to V11.0 and V12.0.
- The International Pancreatitis Study Group has been removed from section 15.2 Dissemination to Key Stakeholders, as this group has not been active during or following the COVID-19 pandemic.
- The list of references has been updated to support these changes.

## 16.13 Version 13.0 (28/09/2023)

The following amendments were made to v12.0 (18/07/2023)

- Total Sexual Abstinence has been defined as per the Clinical Trials Facilitation Group Guidance: Sexual abstinence is considered a highly effective method only if defined as refraining from heterosexual intercourse during the entire period of risk associated with the study treatments. The reliability of sexual abstinence needs to be evaluated in relation to the duration of the clinical trial and the preferred and usual lifestyle of the subject. Wording is included in Section 5.2 Exclusion Criteria.
- The number of patients with sufficiently complete follow up data to carry out the first interim analysis has been reduced further to 15 in each group to reach an earlier conclusion about continuation of the trial, an adjustment that has had minimal effect on the power of the trial (reduced to 86.9% from 89.1% [originally 90%]) and the likelihood of type 1 error (reduced from 0.024 to 0.023 [originally 0.025]) but reduces the time taken to recruit sufficient participants and therefore reduces costs to achieve this milestone. This is included in Section 1 Protocol Summary Schematic of Trial Design, Section 3 Trial Design and Section 10 Statistical Considerations.
- The wording around continuing recruitment during interim analyses in Section 10.4 Interim Monitoring and Analyses has been removed in line with previous discussions with the funder.

## 16.14 Version 14.0 (15/10/2024)

- The sample size was reduced to allow the study to be completed in a more feasible timeframe. The revised sample size is based on reduced statistical power (80%) but all oversight committees have agreed that this will still allow the question to be answered without detriment to the study. Changes are reflected in Section 1 Protocol Summary, Section 3 Trial Design and Section 10 Statistical Considerations.
- Alanine aminotransferase (ALT) five or more times the upper limit of normal in exclusion criterion 8 within Section 5 Study Population has been removed. Exclusion criterion 8 describes liver disease contraindications to infliximab whereas a patient with acute pancreatitis may present with an ALT five or more times the upper limit of normal, which is an accurate diagnostic marker of gallstones as the cause of acute pancreatitis. This value had been included in exclusion criterion 8 as liver injury may develop after treatment with infliximab and a value of ALT five or more times the upper limit of normal is a contraindication to continued treatment with infliximab, based on the assumption that elevation of ALT is caused by ongoing treatment with infliximab, which is not applicable to RAPID-I.
- The supply of infliximab has been changed from provision from MSD (through an Investigator Initiated Clinical Trial Research Agreement) to be sourced from existing hospital stock via usual NHS procurement procedures, as from 1 October 2024 MSD no longer hold a licence for infliximab (as Remicade®). This change is reflected in Section 8 Trial Treatment.

## 16.15 Version 15.0 (13/03/2025)

- Correction in the Schematic of Study Design which incorrectly stated, 'Eligible consenting patients randomised and trial treatment begun within 12 hours of admission'. This text has been amended to 'Eligible consenting patients randomised and trial treatment begun within 36 hours of admission’.

## 16.16 Version 16.0 (24/02/2026)

- To allow for greater than 10% loss of evaluable primary outcome data from 222 patients, which would compromise obtaining evaluable primary outcome data from 66 patients in each of the three arms (provided that no treatment arms are dropped prematurely), the possibility of recruitment of more than 222 patients has been incorporated into the trial design (10.3 Sample Size Calculation). This potential increase in recruitment is designed to ensure evaluable primary outcome data from 66 patients in each arm, which will maintain 80% power for determination of the primary outcome.
- Further details on the statistical methods planned for use in the analysis have been added (10.5 Analysis Plan), as has updated detail on funding (14.2 Funding and Support in Kind).
- The references for the International Association of Pancreatology guidelines and recommendations from the EQUATOR network (TRIPOD, SPIRIT, CONSORT, and CHEERS) have been updated.

# 17 References

1) Szatmary P, Grammatikopoulos T, Cai W et al. Acute pancreatitis: diagnosis and treatment. Drugs 2022; 82: 1251-1276.

2) Peery AF, Crockett SD, Barritt AS et al. Burden of gastrointestinal, liver, and pancreatic diseases in the United States. Gastroenterology 2015; 149: 1731-41.

3) Mounzer R, Langmead CJ, Wu BU et al. Comparison of existing clinical scoring systems to predict persistent organ failure in patients with acute pancreatitis. Gastroenterology 2012; 142: 1476-82.

4) Bakker OJ, Issa Y, van Santvoort HC et al. Treatment options for acute pancreatitis. Nat Rev Gastroenterol Hepatol 2014; 11: 462-9.

5) Moggia E, Koti R, Belgaumkar AP, Fazio F, Pereira SP, Davidson BR, Gurusamy KS. Pharmacological interventions for acute pancreatitis. Cochrane Database Syst Rev 2017; 4: CD011384.

6) Wen L, Voronina S, Javed MA et al. Inhibitors of ORAI1 prevent cytosolic calcium- associated injury of human pancreatic acinar cells and acute pancreatitis in 3 mouse models. Gastroenterology 2015; 149: 481-92.

7) Malleo G, Mazzon E, Siriwardena AK et al. Role of tumor necrosis factor-alpha in acute pancreatitis: from biological basis to clinical evidence. Shock 2007; 28: 130-40.

8) Bishehsari F, Sharma A, Stello K et al. TNF-alpha gene (TNFA) variants increase risk for multi-organ dysfunction syndrome (MODS) in acute pancreatitis. Pancreatology 2012; 12: 113-118.

9) Gukovskaya AS, Gukovsky I, Zaninovic V et al. Pancreatic acinar cells produce, release, and respond to tumor necrosis factor-alpha. Role in regulating cell death and pancreatitis. J Clin Invest 1997; 100: 1853-62.

10) Brenner D, Blaser H, Mak TW. Regulation of tumour necrosis factor signalling: live or let die. Nat Rev Immunol 2015; 15: 362-74.

11) Ho YP, Chiu CT, Sheen IS et al. Tumor necrosis factor-α and interleukin-10 contribute to immunoparalysis in patients with acute pancreatitis. Hum Immunol 2011; 72: 18-23.

12) Malmstrøm ML, Hansen MB, Andersen AM et al. Cytokines and organ failure in acute pancreatitis: inflammatory response in acute pancreatitis. Pancreas 2012; 41: 271-7.

13) Shen Y, Cui NQ. Clinical observation of immunity in patients with secondary infection from severe acute pancreatitis. Inflamm Res 2012; 61: 743-8.

14) Fisic E, Poropat G, Bilic-Zulle L et al. The role of IL-6, 8, and 10, sTNFr, CRP, and pancreatic elastase in the prediction of systemic complications in patients with acute pancreatitis. Gastroenterol Res Pract 2013; 2013: 282645.

15) Stobaugh DJ, Deepak P. Effect of tumor necrosis factor-α inhibitors on drug-induced pancreatitis in inflammatory bowel disease. Ann Pharmacother 2014; 48: 1282-7.

16) Clayton H, Flatz L, Vollenweider-Roten S et al. Anti-TNF therapy in the treatment of psoriasis in a patient with acute-on-chronic pancreatitis. Dermatology 2013; 227: 193-6.

17) Vege SS, Atwal T, Bi Y et al. Pentoxifylline treatment in severe acute pancreatitis: a pilot, double-blind, placebo-controlled, randomized trial. Gastroenterology 2015; 149: 318-20.

18) Magirr D, Jaki T, Whitehead J. A generalized Dunnett test for multi-arm multi-stage clinical studies with treatment selection. Biometrika 2012; 99: 494-501.

19) Magirr D, Stallard N, Jaki T. Flexible sequential designs for multi-arm clinical trials. Stat Med 2014; 33: 3269-79.

20) Micheel CM, Ball JR Eds Evaluation of Biomarkers and Surrogate Endpoints in Chronic Disease. Washington: National Academies Press. 2010.

21) Schütte K, Malfertheiner P. Markers for predicting severity and progression of acute pancreatitis. Best Pract Res Clin Gastroenterol 2008; 22: 75-90.

22) Ordás I, Mould DR, Feagan BG et al. Anti-TNF monoclonal antibodies in inflammatory bowel disease: pharmacokinetics-based dosing paradigms. Clin Pharmacol Ther 2012; 91: 635-46.

23) Brandse JF, Mathôt RA, van der Kleij D et al. Pharmacokinetic features and presence of antidrug antibodies associate with response to infliximab induction therapy in patients with moderate to severe ulcerative colitis. Clin Gastroenterol Hepatol 2016; 14: 251-8.

24) Rosen MJ, Minar P, Vinks AA. Review article: applying pharmacokinetics to optimise dosing of anti-TNF biologics in acute severe ulcerative colitis. Aliment Pharmacol Ther 2015; 41: 1094-103.

25) Regueiro M, Siemanowski B, Kip KE et al. Infliximab dose intensification in Crohn's disease. Inflamm Bowel Dis 2007; 13: 1093-9.

26) Vande Casteele N, Khanna R, Levesque BG et al. The relationship between infliximab concentrations, antibodies to infliximab and disease activity in Crohn's disease. Gut 2015; 64: 1539-45.

27) Steenholdt C, Bendtzen K, Brynskov J et al. Changes in serum trough levels of infliximab during treatment intensification but not in anti-infliximab antibody detection are associated with clinical outcomes after therapeutic failure in Crohn's disease. J Crohns Colitis 2015; 9: 238-45.

28) Yanai H, Lichtenstein L, Assa A et al. Levels of drug and antidrug antibodies are associated with outcome of interventions after loss of response to infliximab or adalimumab. Clin Gastroenterol Hepatol 2015; 13: 522-530.

29) Stern R, Wolfe F. Infliximab dose and clinical status: results of 2 studies in 1642 patients with rheumatoid arthritis. J Rheumatol 2004; 31: 1538-45.

30) Gibson DJ, Heetun ZS, Redmond CE et al. An accelerated infliximab induction regimen reduces the need for early colectomy in patients with acute severe ulcerative colitis. Clin Gastroenterol Hepatol 2015; 13: 330-335.

31) Data on file. Janssen Biotech, Inc.

32) Banks PA, Bollen TL, Dervenis C et al. Classification of acute pancreatitis 2012: revision of the Atlanta classification and definitions by international consensus. Gut 2013; 62: 102-11.

33) Dellinger EP, Forsmark CE, Layer P et al. Determinant-based classification of acute pancreatitis severity: an international multidisciplinary consultation. Ann Surg 2012; 256: 875-80.

34) Ueda T, Takeyama Y, Yasuda T et al. Immunosuppression in patients with severe acute pancreatitis. J Gastroenterol 2006; 41: 779-84.

35) Shen Y, Cui N, Miao B et al. Immune dysregulation in patients with severe acute pancreatitis. Inflammation 2011; 34: 36-42.

36) European Medicines Agency. Guideline on clinical investigation of medicinal products other than NSAIDs for treatment of rheumatoid arthiritis. CPMP/EWP/556/95 Rev. 2; 05 December 2011.

37) Afghani E, Pandol SJ, Shimosegawa T et al. Acute pancreatitis: progress and challenges: a report on an international symposium. Pancreas 2015; 44: 1195-210.

38) Collins GS, Moons KGM, Dhiman P, et al. TRIPOD+AI statement: updated guidance for reporting clinical prediction models that use regression or machine learning methods. BMJ 2024; 385: e078378.

39) Raraty MG, Halloran CM, Dodd S et al. Minimal access retroperitoneal pancreatic necrosectomy: improvement in morbidity and mortality with a less invasive approach. Ann Surg 2010; 251: 787-93.

40) IAP/APA/EPC/IPC/JPS Working Group. International Association of Pancreatology revised guidelines on acute pancreatitis 2025: supported and endorsed by the American Pancreatic Association, European Pancreatic Club, Indian Pancreas Club, and Japan Pancreas Society. Pancreatology 2025; 25: 770-814.

41) Corominas M, Gastaminza G, Lobera T. Hypersensitivity reactions to biological drugs. J Investig Allergol Clin Immunol 2014; 24: 212-25.

42) Lichtenstein L, Ron Y, Kivity S et al. Infliximab-related infusion reactions: systematic review. J Crohns Colitis. 2015; 9: 806-15.

43) Raychaudhuri SP, Nguyen CT, Raychaudhuri SK et al. Incidence and nature of infectious disease in patients treated with anti-TNF agents. Autoimmun Rev 2009; 9: 67-81.

44) Hewitt RJ, Francis M, Singanayagam A et al. Screening tests for tuberculosis before starting biological therapy. BMJ 2015; 350: h1060.

45) Gianfrancesco M, Hyrich KL, Al-Adely S et al. Characteristics associated with hospitalisation for COVID-19 in people with rheumatic disease: data from the COVID-19 Global Rheumatology Alliance physician-reported registry. Ann Rheum Dis 2020; 79: 859-66.

46) Khan N, Patel D, Xie D et al. Impact of Anti-TNF and thiopurines medications on the development of COVID-19 in patients with inflammatory bowel disease: A nationwide VA cohort study. Gastroenterology 2020; 159: 1545-6.

47) Feldmann M, Maini RN, Woody JN et al. Trials of anti-tumour necrosis factor therapy for COVID-19 are urgently needed. Lancet 2020; 395: 1407-9.

48) Fisher BA, Veenith T, Slade D, et al. Namilumab or infliximab compared with standard of care in hospitalised patients with COVID-19 (CATALYST): a randomised, multicentre, multi-arm, multistage, open-label, adaptive, phase 2, proof-of-concept trial. Lancet Respir Med 2022; 10: 255-266.

49) van de Veerdonk FL, Giamarellos-Bourboulis E, Pickkers P, et al. A guide to immunotherapy for

COVID-19. Nat Med 2022; 28: 39-50.

50) Brenner EJ, Ungaro RC, Colombel JF et al. SECURE-IBD Database Public Data Update. covidibd.org. Accessed on 03/12/21.

51) Gianfrancesco M, Hyrich KL, Al-Adely S et al. Characteristics associated with hospitalisation for COVID-19 in people with rheumatic disease: data from the COVID-19 Global Rheumatology Alliance physician-reported registry. Ann Rheum Dis 2020; 79: 859-66.

52) Public Health England. Immunisation against infectious disease – the Green Book. Chapter 14a – COVID-19 – SARS-CoV-2. 17 August 2022. [https://assets.publishing.service.gov.uk/government/uploads/system/uploads/attachment_data/file/1098808/Greenbook_chapter_14A_17August2022.pdf]. Accessed on 22/08/22.

53) Kroger A, Bahta L, Hunter P. General Best Practice Guidelines for Immunization. Best Practices Guidance of the Advisory Committee on Immunization Practices (ACIP). [www.cdc.gov/vaccines/hcp/acip-recs/general-recs/downloads/general-recs.pdf]. Accessed on 22/08/22.

54) Klotz U, Teml A, Schwab M. Clinical pharmacokinetics and use of infliximab. Clin Pharmacokinet 2007; 46: 645-660.

55) Ferreira FL, Bota DP, Bross A et al. Serial evaluation of the SOFA score to predict outcome in critically ill patients. JAMA 2001; 286: 1754-8.

56) van Reenen M, Janssen B. EQ-5D-5L User Guide Version 2.1. EuroQol Research Foundation: 2015.

57) Fleming TR. Identifying and Addressing Safety Signals in Clinical Trials. N Engl J Med 359; 13: 1400-2.

58) Chan AW, Boutron I, Hopewell S, et al. SPIRIT 2025 statement: updated guideline for protocols of randomised trials. BMJ 2025; 389: e081477.

59) Hopewell S, Chan AW, Collins GS, et al. CONSORT 2025 statement: updated guideline for reporting randomised trials. BMJ 2025; 389: e081123.

60) Husereau D, Drummond M, Augustovski F, et al. Consolidated Health Economic Evaluation Reporting Standards 2022 (CHEERS 2022) statement: updated reporting guidance for health economic evaluations. BMJ 2022; 376: e067975.

61) National Institute for Health and Care Excellence (NICE) (2018) Pancreatitis (NICE Guideline 104) Available at: <https://www.nice.org.uk/guidance/ng104/evidence/full-guideline-pdf-6535536157> [Accessed 23 May 2019]

62) McPherson SJ, O'Reilly DA, Sinclair MT et al. The use of imaging in acute pancreatitis in United Kingdom hospitals: findings from a national quality of care study. Br J Radiol 2017; 90: 20170224.

63) Mortele KJ, Wiesner W, Intriere L, et al. A modified CT severity index for evaluating acute pancreatitis: improved correlation with patient outcome. AJR Am J Roentgenol 2004; 183: 1261-1265.

18 Documents Supplementary to the protocol

Documents accompanying the protocol, separately updated and version controlled are:

Participant information sheets and consent forms

GP Letter

Questionnaires
